# Transcriptome analysis in osteoarthritis primary tissues identifies high-confidence effector genes

**DOI:** 10.1038/s41467-026-74993-y

**Published:** 2026-06-29

**Authors:** Georgia Katsoula, Ana Luiza Arruda, Mauro Tutino, Ene Reimann, Norbert Bittner, Peter Kreitmaier, Karan M. Shah, Diane Swift, Lorraine Southam, Siim Suutre, Galadriel Lucía Velázquez Silva, Kaspar Tootsi, Aare Märtson, Reedik Mägi, J. Mark Wilkinson, Eleftheria Zeggini

**Affiliations:** 1 Technical University of Munich (TUM), TUM University Hospital, TUM School of Medicine and Health, Munich, Germany; 2 Institute of Translational Genomics, Helmholtz Zentrum München–German Research Center for Environmental Health, Neuherberg, Germany; 3 Medical Research Council (MRC) Epidemiology Unit, University of Cambridge, Cambridge, UK; 4 Estonian Genome Centre, Institute of Genomics, University of Tartu, Tartu, Estonia; 5 School of Medicine and Population Health, University of Sheffield, Sheffield, UK; 6 Department of Anatomy, University of Tartu, Tartu, Estonia; 7 Department of Orthopaedics, University of Tartu, Tartu, Estonia; 8 Orthopaedics Clinic, Tartu University Hospital, Tartu, Estonia

**Keywords:** Osteoarthritis, Genetics research, Molecular medicine

## Abstract

Osteoarthritis, a whole-joint degenerative disorder, is a major public health burden that affects nearly 600 million individuals worldwide, with no disease-modifying treatment. Molecular profiling of relevant tissues is crucial for understanding the biology of disease development. Here, we generate a comprehensive map of *cis*- and *trans*- transcriptional regulation in disease-relevant primary tissues from knee osteoarthritis patients: macroscopically intact (low-grade, n = 261) and degenerated (high-grade, n = 212) cartilage, synovium (n = 277), and fat pad (n = 92). We identify 10,166 unique expression quantitative trait loci (eQTL)-associated genes, 61.1% of which have not been reported in previous osteoarthritis eQTL studies, and uncover cartilage grade-specific genetic regulation. Using the largest osteoarthritis genome-wide association study to date, we find colocalization evidence with 136 genes and prioritize 45 high-confidence effector genes. At colocalizing loci, osteoarthritis risk alleles are associated with increased expression of genes involved in chondrogenic and hypertrophic signaling and decreased expression of genes encoding regulatory and modulatory components of these pathways. By integrating the eQTL maps with functional data, we delineate regulatory architectures for osteoarthritis risk variants, including promoter-enhancer loops and transcription factor binding effects. Finally, we provide directional evidence highlighting drugs targeting *LGALS3* and *SMAD7* as repurposing opportunities for osteoarthritis treatment.

## Abstract (web summary)

Osteoarthritis is a major public health burden. The authors performed molecular profiling of four osteoarthritis-relevant tissues and identify key biological mechanisms underlying knee osteoarthritis, highlighting drug repurposing opportunities

## Introduction

Osteoarthritis, the most prevalent form of arthritis, is a chronic degenerative complex disease that is projected to affect over one billion individuals worldwide by 2050^1^. Osteoarthritis is characterized by progressive breakdown of articular cartilage and low-grade inflammation of the surrounding tissues^2,3^, leading to pain, stiffness, and reduced mobility^4^. It most commonly affects the knee, hand, and hip joints^5^. In addition to risk factors such as age, obesity, and joint injury, osteoarthritis also has a genetic risk component. Currently, there is no effective disease-modifying treatment for osteoarthritis, and strategies focus on pain management and joint replacement surgery. Therefore, there is an urgent need for an in-depth understanding of the processes underpinning osteoarthritis to foster the development of new therapies. Genome-wide association studies (GWAS) have identified over 960 independent risk variants for osteoarthritis^6^, the majority of which reside in non-coding regions of the genome. Thus, the key challenge is to identify the effector genes through which these associated variants affect osteoarthritis risk.

Osteoarthritis is a whole-joint disease, driven by a complex interplay of both shared and tissue-specific mechanisms^7^. Shared mechanisms involve inflammatory pathways, biomechanical stress, and metabolic imbalances that collectively contribute to the degeneration of articular cartilage, the primary hallmark of osteoarthritis^4^. This includes extensive changes in the extracellular matrix (ECM), including loss of proteoglycans and collagen through enzymatic cleavage, mediated by metalloproteinases and aggrecanases, and loss of chondrocyte ECM maintenance properties through senescence and apoptosis. In addition to cartilage degradation, osteoarthritis affects the synovium, subchondral bone, and fat pad tissues. The synovium commonly exhibits inflammation (synovitis) characterized by increased production of pro-inflammatory cytokines, contributing to cartilage degradation and joint inflammation^2^. Pro-inflammatory cytokines are also secreted by the adipose tissue located in the infrapatellar fat pad^8^. There is evidence that crosstalk between the synovium and fat pad could stimulate the infiltration of immune cells and the secretion of catabolic molecules across both tissues^9^. Subchondral bone undergoes abnormal remodeling and sclerosis, influenced by altered bone turnover and microdamage repair processes^10^. These interconnected pathophysiological changes underscore the importance of studying multiple primary tissues.

Expression quantitative trait loci (eQTLs) capture the association between genetic variation and tissue-specific gene expression (proximal or distal)^11^. The regulatory relationships captured by eQTLs can generate hypotheses for the function of disease-associated variants identified through GWAS^12^. Given the tissue- and context-specificity underlying gene regulatory patterns^13^, eQTL regulatory relationships are best captured in the context of the studied phenotype in the primary affected tissue(s).

There have been two *cis-*eQTL studies in osteoarthritis primary tissues to date. Steinberg et al.^14^, an earlier study from our group, identified *cis*-eQTLs in low-grade cartilage, high-grade cartilage, and synovium from up to 95 osteoarthritis patients of European ancestry. This study identified colocalization of eQTLs with osteoarthritis GWAS risk loci spanning five effector genes (*ALDH1A2*, *NPC1*, *SMAD3*, *FAM53A*, *SLC44A2*)^15^. A second study identified *cis-*eQTLs in the synovium of 202 Han Chinese individuals with osteoarthritis. The authors found evidence of colocalization with osteoarthritis GWAS loci for 34 genes with two, *FAM53A* and *SLC44A2*, overlapping with the European ancestry eQTL study^16^. These studies provide valuable insights into the proximal gene regulation mechanisms in osteoarthritis primary tissue. However, a well-powered eQTL map across multiple human joint tissues, including *trans-*acting variants, is still missing.

Here, we have enhanced the sample size of our previous study^14^ and generated *cis-* and *trans-*eQTL maps from knee joint primary tissues of 320 osteoarthritis patients of European ancestry, including macroscopically intact (low-grade) and degraded (high-grade) cartilage, synovium, and fat pad. Fat pad has not been included in previous osteoarthritis eQTL studies, and the sample size for the other three tissues is increased up to threefold compared to previous studies. We identify shared and distinct regulatory patterns across primary osteoarthritis joint tissues and integrate this resource of gene expression molecular data with knee-related osteoarthritis GWAS to gain insights into loci associated with disease risk.

## Results

### Osteoarthritis primary tissues *cis-*eQTL discovery

**Fig. 1:**
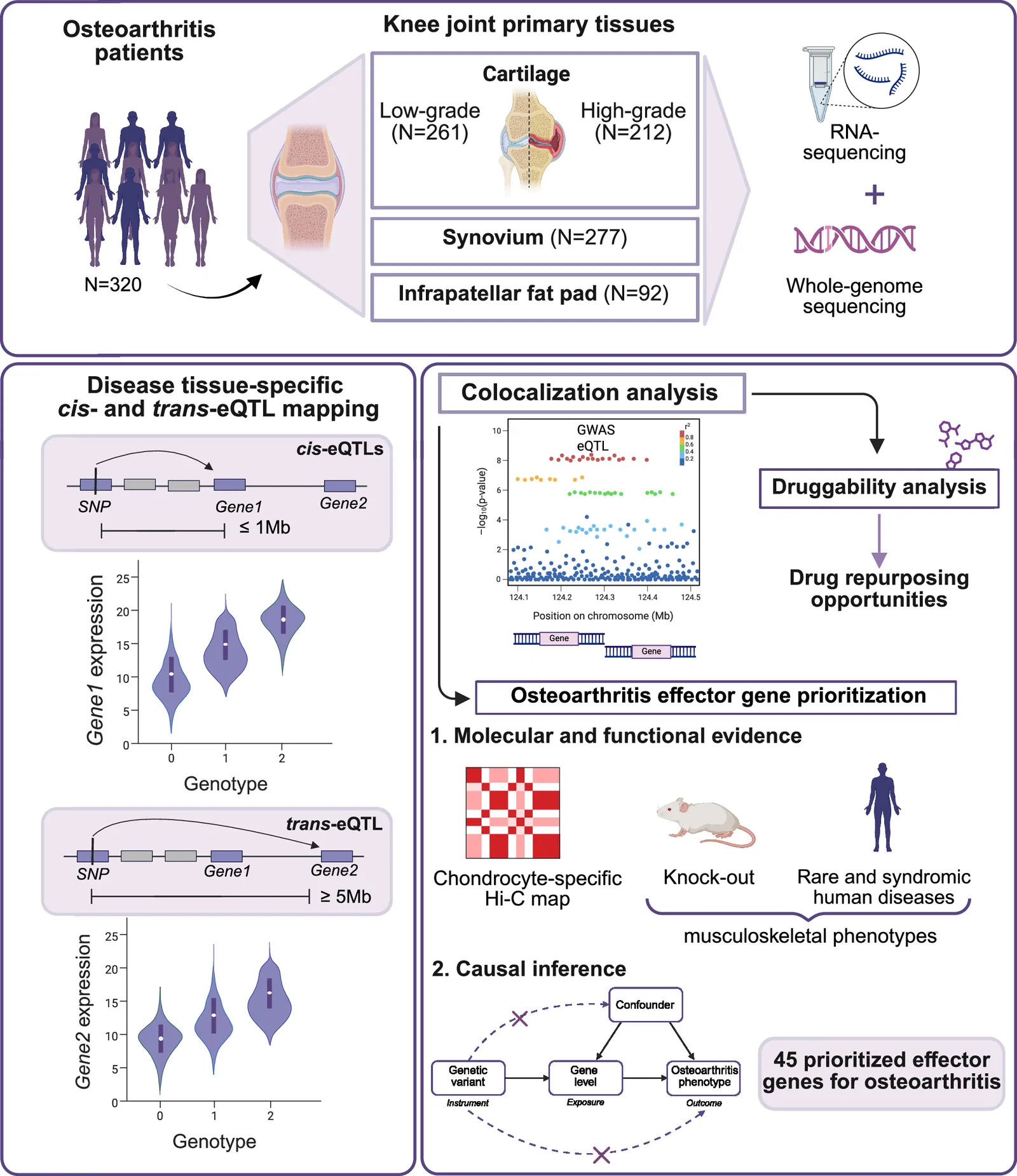
Study design. Violin plots and the regional association plot shown are schematic illustrations of *cis*-/*trans*-eQTL and colocalization analyses and do not represent real data. Created in BioRender. Katsoula, G. (2026) https://BioRender.com/e38p648.

**Fig. 2:**
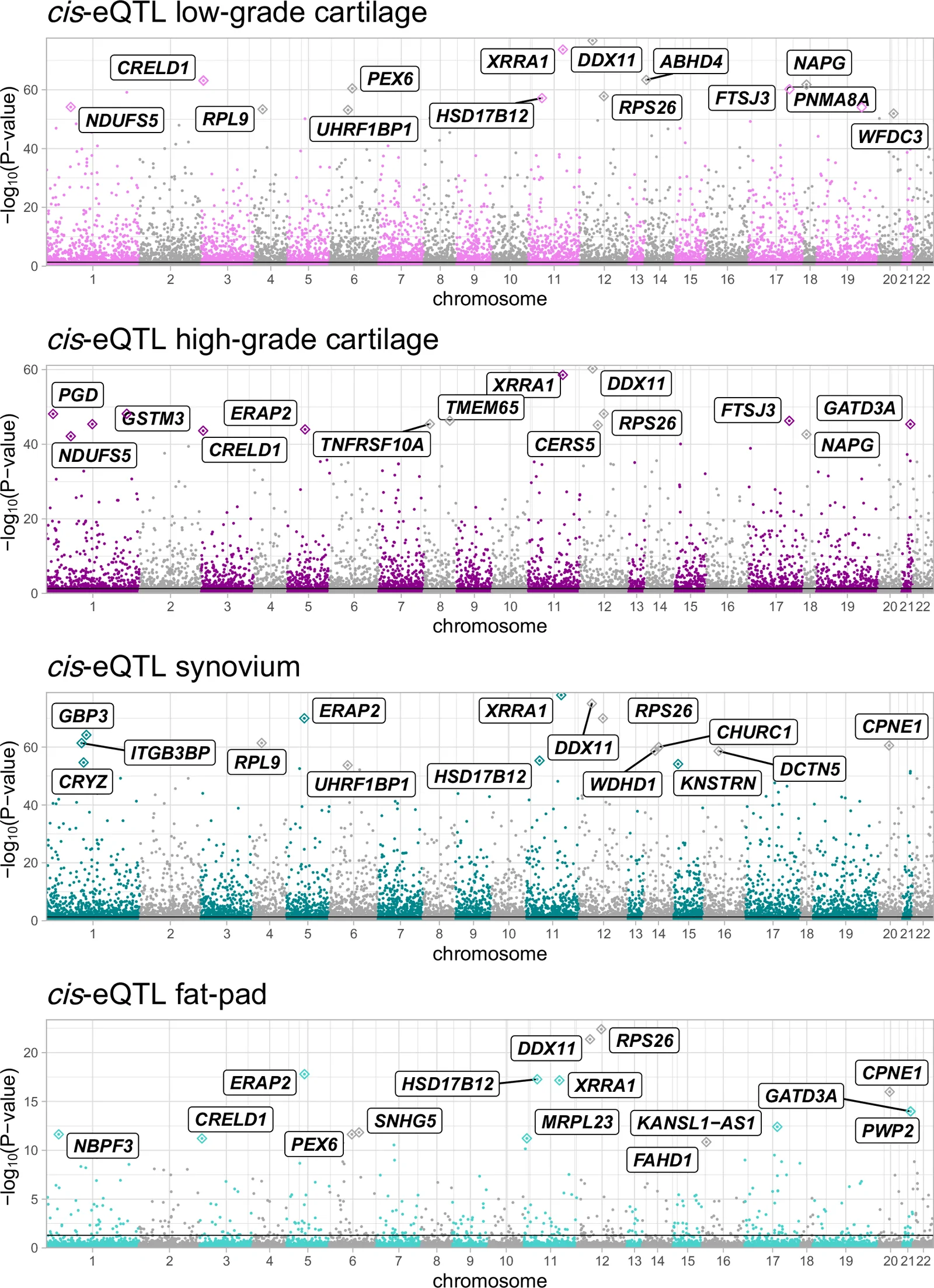
Genomic distribution of *cis-*eQTLs. Manhattan plots depicting the negative log_10_ of the permutation *p*-value (*q*-value) of the most significant association per gene across all variants within 1 Mb of the gene’s transcription start site for low-grade cartilage, high-grade cartilage, synovium, and fat pad tissues. Top *cis-*eGenes per disease tissue are labeled in black. *Cis*-eQTLs were mapped by linear regression (tensorQTL); significance was determined from gene-level permutation (1000 permutations) with Storey-Tibshirani *q*-value correction for multiple testing. Source data are provided as a Source Data file.

We performed *cis*-eQTL analyses in 320 knee osteoarthritis patients, representing a > threefold increase in sample size compared to our previous study^14^ (Fig. 1, Supplementary Fig. 1, and Supplementary Data 1). Consistent with this increase, power analyses indicated increased sensitivity to detect common variants with moderate-to-large *cis*-eQTL effects relative to earlier sample sizes (Supplementary Fig. 1). We replicate previously reported eQTL signals (~70% across tissues: 69.4% in synovium, 68.5% in low grade cartilage, and 68.6% in high-grade cartilage), providing continuity with prior findings (Supplementary Fig. 2). We identify 6874 *cis-*eGenes (genes targeted by at least one eQTL) in low-grade cartilage and 4809 in high-grade cartilage, including 8718 and 5560 conditionally independent *cis*-eQTLs, respectively (Fig. 2 and Supplementary Data 2). In synovium, we find 6938 *cis-*eGenes targeted by 8862 conditionally independent *cis*-eQTLs (Fig. 2 and Supplementary Data 2), and in fat pad osteoarthritis tissue, we identify 526 *cis-*eGenes regulated by 537 conditionally independent *cis-*eQTLs (Fig. 2 and Supplementary Data 2). The majority of eQTLs overlap non-coding intronic and intergenic regions in agreement with other studies^17^ (Supplementary Fig. 3).

### Distinct and shared eQTL effects in osteoarthritis tissues

**Fig. 3:**
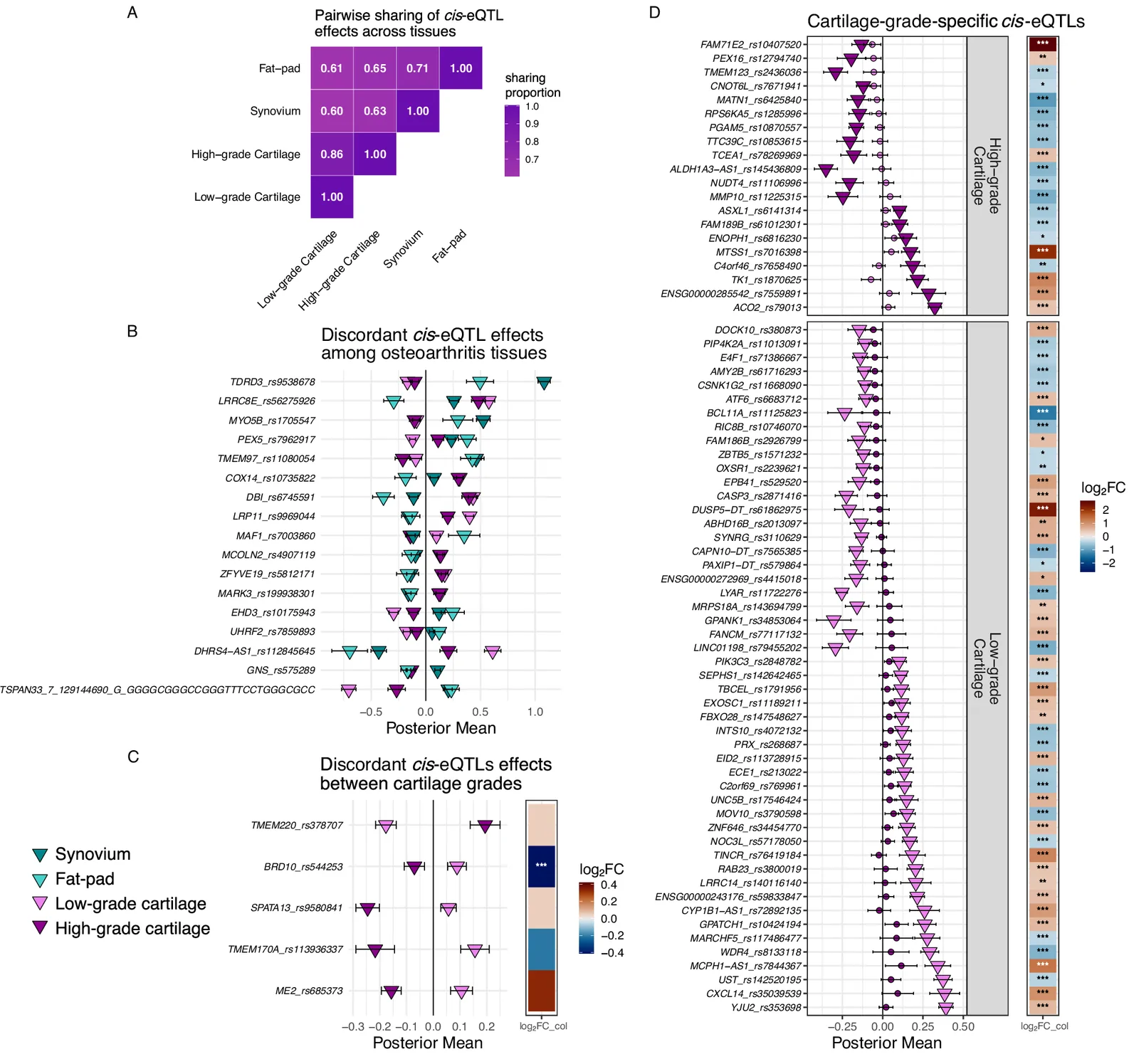
Patterns of shared and discordant eQTL effects among osteoarthritis primary tissues. **A** Heatmap showing pairwise sharing by magnitude of *cis*-eQTLs among tissues. **B** 17 eQTL-eGene pairs with opposite directions of effect between tissues; points show the mashr posterior mean and error bars the posterior standard deviation. **C** Five eQTL-eGene pairs with opposite directions of effect between low- and high-grade osteoarthritis cartilage, indicating stage-specific regulatory changes; points and error bars as in (**B**). **D** eQTL-eGene pairs unique to each cartilage grade that influence genes differentially expressed (DE) between low- and high-grade cartilage; points and error bars as in (**B**). In (**C**) and (**D**), the heatmap shows DE effect size (log_2_ fold change; reference group: low-grade cartilage); significance is from the DE analysis of ref. ^19^ (two-sided limma-voom, Benjamini-Hochberg FDR): *FDR < 0.05, **FDR < 0.01, ***FDR < 0.001. Estimates in (**B**–**D**) are from matched biological tissue samples from unrelated patients (low-grade cartilage *n* = 261, high-grade cartilage *n* = 212, synovium *n* = 277, fat pad *n* = 92). Source data are provided as a Source Data file.

We sought to identify distinct genetic regulatory effects present in individual disease tissues using two complementary approaches. Firstly, we investigated eQTLs regulating genes expressed exclusively in a single disease tissue and not in others based on a minimum gene expression threshold (see “Methods”), identifying 606 unique eGenes in cartilage, 74 in synovium, and 13 in fat pad (Supplementary Data 3). Secondly, to formally assess distinct genetic regulatory patterns across osteoarthritis tissues for commonly expressed genes, we applied mashr^18^, which models shared and distinct patterns of genetic regulation across all examined disease tissues (low-grade cartilage, high-grade cartilage, synovium and fat pad), and identify 62 additional eGenes with eQTL effects exclusive to low-grade cartilage, 23 to high-grade cartilage, 385 to synovium, and 70 to fat pad, with no effect in any other analyzed osteoarthritis tissue (Supplementary Fig. 4 and Supplementary Data 4). In line with GTEx findings^17^, we observe widespread sharing of eQTL effects across osteoarthritis-relevant tissues, reflecting common regulatory architecture. Specifically, 71% of *cis*-eQTLs are shared across all four tissues when considering both effect direction and magnitude (see “Methods”). Tissue pairs with closer biological similarity show even greater overlap, with 86% of *cis*-eQTLs shared between low- and high-grade cartilage, and 71% between synovium and fat pad (Fig. 3A).

Nevertheless, a subset of 17 eQTL-eGene pairs, though significant across all disease-relevant tissues, exhibit discordant effects (Supplementary Data 4 and 5), showing opposite directions of association in at least one tissue (Fig. 3B). In 12 out of 17 cases, the direction of effect was concordant between low- and high-grade cartilage, as well as between synovium and fat pad, reflecting greater similarity within these tissue pairs.

### Cartilage-grade dependent eQTLs reveal stage-specific genetic regulation

Cartilage was the only tissue that included both low- and high-grade osteoarthritis samples, enabling the investigation of stage-specific regulatory changes. We identified 97 eQTL-eGene pairs with effects exclusive to low-grade cartilage and 46 pairs with effects only in high-grade cartilage (Supplementary Data 6 and 7).

In order to understand if the differential regulation between high- and low-grade cartilage is associated with expression changes related to progression of cartilage degeneration, we further queried the differentially regulated eGenes against differentially expressed genes between high- and low-grade cartilage^19^, defining differentially expressed eGenes (DE-eGenes). We find that 70 out of the 143 differentially regulated eGenes are also differentially expressed between high- and low-grade cartilage, indicating coordinated changes in both genetic regulation and transcriptional output as osteoarthritis progresses. Despite no significant enrichment of these genes for a specific pathway top DE-eGenes for low-grade cartilage include RNA-processing and splicing regulation components (*YJU2*, *GPATCH1*) and several lncRNAs (*MCPH1-AS1*, *LINC01198*, *CAPN10-DT*), while for high-grade cartilage we find genes involved in mitochondrial and metabolic processes (*ACO2*, *PGAM5*, *ENOPH1*, *PEX16*), ECM remodelling (*MATN1*, *MMP10*) and cell-cycle regulation (*TK1*, *ASXL1*). Together, these findings suggest that regulatory variation in advanced-stage osteoarthritis increasingly impacts genes involved in cellular stress responses, matrix regulation, and transcriptional reprogramming.

Notably, we identified five eQTL-eGene pairs that exhibited significant associations in both low- and high-grade cartilage, but with opposite directions of effect (Fig. 3C and Supplementary Data 7). These included *SPATA13*, *TMEM220, TMEM17A, BRD10*, and *ME2*, out of which only *BRD10* is also differentially expressed between cartilage grades (downregulated in high-grade cartilage) (Fig. 3D). Such directionally discordant effects may reflect dynamic, stage-specific regulatory mechanisms, potentially altering gene function or cellular context as osteoarthritis advances.

### *Trans*-eQTLs in osteoarthritis primary tissues

We investigated distal gene regulation (≥5 Mb from transcription start sites) in osteoarthritis primary tissues. In low-grade cartilage, we identify 13 independent signals regulating 13 *trans*-eGenes; in high-grade cartilage, 4 independent *trans*-eQTLs associated with 4 genes; in synovium, 4 independent *trans-*eQTLs associated with 4 genes; and no *trans*-eQTLs in fat pad. Four *trans*-eGenes showed cross-tissue overlap. *UQCRHL* was shared across all tissues. *ORC6*, *MYLK3*, and *KANSL1-AS1* were common to low- and high-grade cartilage, while *CDK10* was shared between high-grade cartilage and synovium (Supplementary Data 8).

To identify putative regulatory relationships between *trans*-eGenes and *cis*-eGenes we assessed whether *trans*-eQTLs also exert *cis* effects for additional genes. We identify 129 such *trans*-eQTLs also acting in *cis* cases in low-grade cartilage, 93 in synovium, and 47 in high-grade cartilage. In all three tissues, *trans*-eQTL signals for *UQCRHL*, acted in *cis* for *UQCRH*, pointing to a shared mitochondrial regulatory axis. In synovium and high-grade cartilage, additional *cis* targets of *UQCRHL* (*trans*-eGene) include *FAAH* and *LURAP1* (synovium) and *TSPAN1* (high-grade cartilage), respectively (Supplementary Data 8).

In low-grade cartilage, we uniquely find that *trans*-eQTLs for *CBX3* act in *cis* for *CCDC32*. These two genes have been previously found in a gene fusion in healthy blood and bone marrow tissues^20^. In the synovium, we also uniquely identify that *FHAD1 trans*-eQTLs are also *cis*-eQTLs for *ZNF100*, suggesting a potential mediation relationship. Notably, *ZNF100* has been reported as a candidate effector gene for synoviopathy in Open Targets^21^. Complementarily, we also evaluated whether *trans*-eGenes were themselves also *cis*-regulated. We find that 11/13 in low-grade cartilage, 3/4 in high-grade cartilage, and 2/4 in synovium are also *cis*-eGenes in the same tissues. Out of these, notable examples include *CRHR1* and *KANSL1-AS1*, both located in the osteoarthritis-associated 17q21.31 locus, which also show elevated expression in knee osteoarthritis cartilage compared to non-osteoarthritis controls^22^.

To explore potential mechanisms underlying these distal regulatory effects, we next asked whether *trans*-eQTL variants overlap regulatory regions. We find 84 *trans*-eQTLs in synovium, 35 in low-grade cartilage, and 16 in high-grade cartilage overlapping regulatory regions (Supplementary Data 9 and Supplementary Fig. 3). Using this subset of *trans*-eQTLs we further assessed whether they might influence gene expression by affecting transcription factor binding sites using motifbreakR^23^. We restricted this analysis for the transcription factor coding genes expressed in each tissue. Across all tissues, we identified 17 unique variants predicted to affect transcription factor binding motifs (Supplementary Data 9). In synovium, two variants overlapping enhancer regions and associated with decreased *TRIM69* expression (rs73827374:C, rs76118907:G) are predicted to affect affinity to retinoic acid-related binding sites (rs73827374:C decreases affinity for RORA binding and rs76118907:G increasing affinity for RARA::RXRA binding).

### Resolution of osteoarthritis GWAS signals

We conducted statistical colocalization analysis between primary knee osteoarthritis tissue *cis*-eQTLs and GWAS risk loci from three knee-related osteoarthritis phenotypes: osteoarthritis at any site (ALLOA), knee osteoarthritis (KNEE), and total knee replacement (TKR) (Supplementary Data 10 and 11). Among the 211 osteoarthritis GWAS risk loci examined, 74 (34.1%) showed evidence of a shared association signal with *cis*-eQTLs from primary osteoarthritis tissues, spanning 136 unique genes (65 in low-grade cartilage, 59 in high-grade cartilage, 66 in synovium, and 5 in fat pad). Eighty-three out of the 136 (61.03%) colocalizing genes have not been previously prioritized as effector genes for osteoarthritis in the latest osteoarthritis GWAS meta-analysis^6^. Among these, we find nine genes that also have unique eQTL effects in cartilage tissue (low- or high-grade) (*LINC01770, COL9A1, COL10A1, CHRDL2, SSTR5-AS1, SSTR5, CLEC18A, CLEC18C, ENSG00000273113*), five in synovium (*ITIH3*, *HMGA2-AS1*, *SLC25A13*, *DOT1L*, *CRADD*), and one in fat pad (*TBX2-AS1*).

**Fig. 4:**
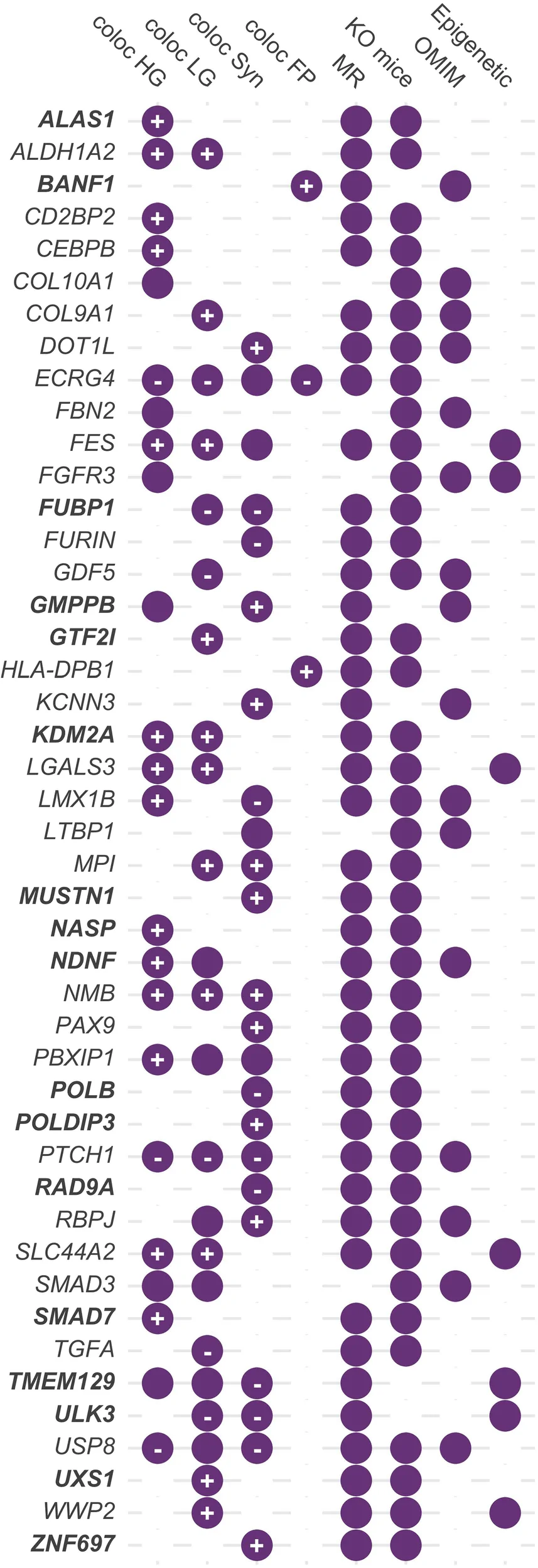
Prioritized colocalizing genes based on additional evidence of involvement in knee-related osteoarthritis. In addition to showing the osteoarthritis primary tissue of colocalization (HG high-grade cartilage, LG low-grade cartilage, Syn synovium, FP fat pad), the heat map visualizes the lines of evidence used to assess involvement in disease, including evidence of knockout mouse models (KO mice), rare and syndromic diseases (OMIM), causal inference analysis (MR Mendelian Randomization), overlap with active promoters or active promoter-enhancer loops from chondrocytes (Epigenetic). The signs (+/−) in the colocalization columns indicate the direction of putative causal effect identified by MR. The genes highlighted in bold indicate genes that were not previously prioritized as effector genes for osteoarthritis in the latest disease GWAS meta-analysis^6^. Source data are provided as a Source Data file.

To infer putative causal effects of the identified colocalizing eGenes on knee-related osteoarthritis phenotypes, we conducted tissue-specific causal inference analyses using Mendelian randomization (MR)^24^ (Supplementary Data 12). Out of the 136 colocalizing genes, 99 show evidence of a causal effect on knee-related osteoarthritis for matching disease phenotype and tissue^25^. In addition to causal inference evidence, we have scored the genes based on orthogonal lines of evidence, including knockout mouse phenotypes^26^, rare and syndromic human disease phenotypes^27^, and Hi-C information in cartilage^28^ (Supplementary Data 13). Out of the 136 colocalizing genes, 51 are associated with musculoskeletal-related phenotypes (see “Methods”) in knockout mouse models, and 16 are found to be involved in rare and syndromic human diseases. We prioritize 45 genes that showed at least two lines of evidence pointing to their involvement in osteoarthritis, in addition to colocalizing with GWAS signals (Fig. 4). Of these, 17 have not been defined as osteoarthritis effector genes in the latest GWAS meta-analysis^6^. For instance, we find evidence of a risk-increasing effect of *NDNF* expression on knee osteoarthritis risk in high-grade cartilage (OR = 1.11 (95% CI 1.08–1.13), *q*-value = 4.08 × 10^−14^). Knockout mice for *NDNF* show skeletal muscle atrophy. Mutations in this gene are linked to the rare human disease hypogonadotropic hypogonadism 25 with anosmia, which is characterized by delayed or absent puberty and is associated with delayed bone age. *NDNF* promotes neuron migration, growth, and survival, and may also be involved in regulating growth factor activity^29^. Neurotrophic factors can modulate inflammation and pain perception, an important symptom for osteoarthritis^30,31^.

To further understand the spatial context of the prioritized genes colocalizing with osteoarthritis, we evaluated the expression patterns of the genes using spatial transcriptomics data from degraded osteoarthritis cartilage (see “Methods”). We find that all 27 prioritized genes colocalizing with osteoarthritis in cartilage are expressed in at least one of the analyzed cartilage zones (Supplementary Data 14), with all being detected in the superficial layer of osteoarthritis cartilage. Seven genes (*ALDH1A2, FBN2, LMX1B*, *NDNF*, *NMB*, *SMAD7*, and *TMEM129*) are also expressed in the middle zone, while two genes (*ALDH1A2* and *RBPJ*) are expressed in the deep cartilage zone. This shows that the majority of prioritized genes are active in the superficial layer of osteoarthritis cartilage. This layer is the first point of mechanical loading in the joint and exhibits the earliest pathological changes in osteoarthritis. It contains specialised superficial chondrocytes that produce key homeostatic proteins, including lubricin and tenascin, which are essential for cartilage lubrication and surface integrity. The observed enrichment, therefore, highlights the central contribution of superficial-zone dysfunction to early disease pathogenesis^32^.

### Biological pathways and direction of effect at colocalizing loci

**Fig. 5:**
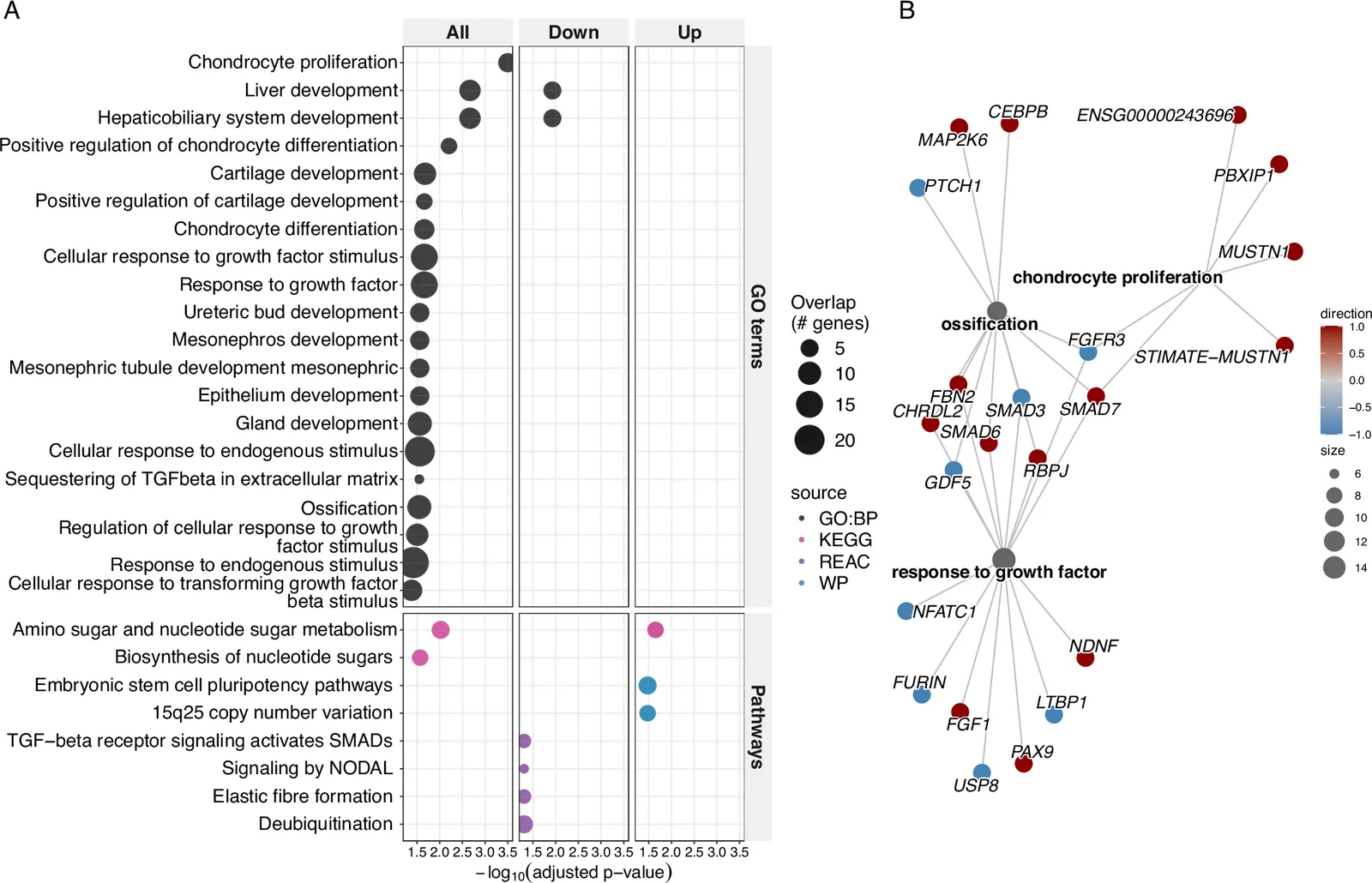
Pathway enrichment analysis of the colocalizing genes. **A** Results (*q*-value < 0.05) of pathway-based over-representation analysis on the 136 genes colocalizing with knee-related osteoarthritis phenotypes. All eGenes across all four primary osteoarthritis tissues studied here were used as the background. Down refers to genes that are downregulated relative to knee osteoarthritis’ risk allele, whereas up refers to genes that are upregulated. (GO:BP Gene Ontology biological processes, REAC Reactome, WP WikiPathways). Enrichment was assessed by one-sided over-representation analysis (g:Profiler2, gost) using a custom background of all eGenes, with Benjamini–Hochberg FDR correction; terms with FDR < 0.05 were considered significant. **B** Network of genes involved in the most strongly enriched processes of the pathway-based over-representation analysis. Red circles denote genes with a knee osteoarthritis risk-increasing effect when upregulated, whereas blue circles denote genes largely risk-increasing when downregulated. The grey circle size denotes the number of genes in the specific pathways. Source data are provided as a Source Data file.

To glean biological insights from the 136 colocalizing genes, we evaluated their enrichment among biological pathways using all eGenes across tissues as background (Supplementary Data 15). We find that colocalizing genes are enriched for chondrocyte proliferation (*q*-value = 3.2 × 10^−3^), cartilage development (*q*-value = 0.021), ossification (*q*-value = 0.028) as well as signaling through transforming growth factor beta (TGF-β) (*q*-value = 0.042) (Fig. 5A). These processes have been previously implicated in osteoarthritis^33–35^. We also performed enrichment analyses separately for each tissue, colocalizing genes implicating processes such as amino sugar and nucleotide sugar metabolism (*q*-value = 0.005) and osteoblast differentiation (*q*-value = 0.048) in low-grade cartilage, osteoarthritic chondrocyte hypertrophy (*q*-value = 0.012) and ossification (*q*-value = 0.045) in high-grade cartilage and chondrocyte proliferation (*q*-value = 0.002) in synovium (Supplementary Data 15).

To refine the biological interpretation of the colocalizing loci, we further examined the direction of allelic effects for each variant in the credible sets. For each colocalization, the osteoarthritis risk allele showed either consistently concordant effects on gene expression (risk allele increases expression) or consistently discordant effects (risk allele decreases expression). This enabled us to classify colocalizing genes into putative “risk-increasing when upregulated” and “risk-increasing when downregulated” groups. Only one gene (*LMX1B*) showed opposite classifications in high-grade cartilage and synovium relative to knee osteoarthritis risk. When evaluating pathways separately for the two groups, the enrichment patterns were modest (Supplementary Data 15), reflecting that osteoarthritis genetic risk perturbs both activating and inhibitory components of the same regulatory pathways (Fig. 5A).

Consistent with this, the most strongly enriched processes are represented by a connected network of TGF-β/BMP signaling components (*SMAD3*, *SMAD6*, *SMAD7*, *GDF5*), FGFR signaling (*FGFR3*), matrix modulators (*FBN2*, *CHRDL2*), and lineage regulators (*MUSTN1*, *PBXIP1*, *CEBPB*, *PAX9, NFATC1*). In this network, “risk-increasing when upregulated” genes largely correspond to pro-chondrogenic and pro-hypertrophic drivers, whereas “risk-increasing when downregulated” genes capture feedback inhibitors and ECM/growth-factor modulators, illustrating that osteoarthritis risk putatively acts by both amplifying chondrogenic drive and weakening its restraint (Fig. 5B).

Notable examples of genes within the aforementioned pathways that we implicate as osteoarthritis effector genes include *SMAD7* and *MUSTN1*. *SMAD7* encodes an intracellular inhibitory SMAD protein that acts as a negative regulator of the TGF-β signaling pathway. TGF-β promotes chondrocyte proliferation and ECM synthesis, preserving cartilage integrity^34^. Variants associated with the expression of *SMAD7* in high-grade cartilage colocalize with total knee replacement (PP4 = 0.76, PP4/PP3 = 3.13) and show evidence of a putative risk-increasing causal effect on total knee replacement risk (OR = 1.22 (95% CI 1.12–1.34), *q*-value = 1.6 × 10⁻⁵). *SMAD7* is the target of mongersen, a SMAD-7 mRNA antisense inhibitor in clinical trials for Crohn’s disease and ulcerative colitis. By inhibiting SMAD7, Mongersen aims to restore TGF-β activity, pointing to a potential repurposing opportunity of this drug for osteoarthritis. Knockout mouse models for *SMAD7* present abnormal bone marrow cell physiology^33^, which can be associated with bone marrow lesions common at late stage osteoarthritis.

Variants associated with the expression of *MUSTN1* in synovium colocalize with knee osteoarthritis GWAS (PP4 = 0.89). In the same tissue, we find evidence of a risk-increasing effect of *MUSTN1* expression on knee osteoarthritis (OR = 1.11 (95% CI 1.08–1.13), *q*-value = 7.45 × 10⁻¹⁸). *MUSTN1* has been implicated in critical processes for maintaining cartilage integrity and function, such as chondrogenesis, chondrocyte differentiation, and proliferation^36^. The protein encoded by *MUSTN1* is predominantly expressed in skeletal muscle and bone and plays a role in myoblast differentiation, bone regeneration, chondrogenesis, and muscle hypertrophy^37^. Knockout mouse models for this gene show skeletal muscle fibrosis^38^.

### Integration with functional data reveals mechanistic insights for osteoarthritis risk loci

We then investigated potential mechanisms of action of the identified eQTLs. For each colocalizing signal, we calculated a 95% credible set of variants, within which the shared causal variant lies with 95% probability. For low- and high-grade cartilage, we studied the location of these variants by overlaying them with publicly available high-throughput chromosome conformation capture data (Hi-C) from primary knee osteoarthritis chondrocytes^28^, ATAC-seq^39^ (on primary intact and degraded cartilage), and ChiP-seq data (from knee articular cartilage samples) in the setting of osteoarthritis as well as information from the ENCODE-SCREEN database^40^. Using these, we classified each credible set variant as overlapping “active promoter”, “active enhancer” and if either were in an active enhancer-promoter loop^28^ (Supplementary Data 16 and Supplementary Fig. 5). For synovium and fat pad, where no publicly available Hi-C, ATAC-seq, or ChIP-seq data are available, we used promoter-level annotations from the SCREEN database^40^ to identify variants overlapping active promoters, allowing us to capture promoter-proximal regulatory activity (Supplementary Data 16 and Supplementary Fig. 5).

In low-grade cartilage, we identify nine credible set variants overlapping active promoters, five of which are also connected to an enhancer in promoter–enhancer loops: rs1800801 (*MGP*), rs2290574 (*ULK3*), rs35346340 (*FES*), rs930392 (*CORO1A*), and rs2102066 (the *MIR140*/*WWP2* locus). We additionally find eight credible set variants overlapping active enhancers, six of which loop to active promoters: rs1894400 and rs4932373 (enhancers looping to the *FES*/*CRTC3-AS1* regulatory region; eGene: *FES*), rs10048090 (looping to *WWP2*/*NOB1*; eGenes: *WWP2*, *CLEC18A*, *EXOSC6*), rs7187586 (looping to *LMF1*; eGenes: *C1QTNF8*, *SSTR5, SSTR5-AS*1), and rs10405617 and rs10948 (looping to *SLC44A2*/*AP1M2*/*CDKN2D*/*KRI1*; eGene: *SLC44A2*) (Table 1 and Supplementary Data 16).

**Table 1:** Credible set variants overlapping regulatory (enhancer/promoter) regions

Locus	Risk	Top trait	GWAS lead	Effector gene	Tissue	Region	Risk effect on expression	TF motif
rs10048090 rs2102066	A A	KNEE	rs10048129	*WWP2* (rs10048090) *WWP2, CLEC18A* (rs2102066)	LG	Enh Pr	*WWP2* ↑ *WWP2* ↑ *CLEC18A* ↓	–
rs12904605	C	ALLOA	rs11633450	*NMB*, *ZNF592*, *ZSCAN2*, *SEC11A*	Syn	Pr	*NMB* ↑ *ZNF592* ↑ *ZSCAN2* ↑ *SEC11A* ↑	–
rs11731421	A	KNEE	rs11731421	*FGFR3*	HG	Enh	*FGFR3* ↓	KLF15
rs12129705	A	ALLOA	rs12129705	*ZNF697*	Syn	Pr	*ZNF697* ↑	–
rs12218394	C	TKR	rs12764568	*EEF1AKMT2*	Syn	Pr	*EEF1AKMT2* ↓	–
rs137080	G	ALLOA	rs137130	*RRP7A*	Syn	Pr	*RRP7A* ↓	–
rs7937	T	KNEE	rs146652543	*RAB4B*, *C19orf54*	Syn	Pr	*RAB4B* ↓ *C19orf54* ↓	ZNF530
rs11539637 rs35346340 rs4932178	T G C	ALLOA	rs1894401	*FES* *FES*, *FURIN* *FES*, *FURIN*	Syn LG, HG, Syn Syn	Pr Pr Pr	*FES* ↑ *FES* ↑ *FURIN* ↓ *FES* ↑ *FURIN* ↓	ZNF343 (rs11539637) KLF15;TWIST1;ZNF281;ZNF320;ZNF530 (rs35346340) −(rs4932178)
rs10405617 rs10948	A G	KNEE	rs3843750	*SLC44A2* *SLC44A2*	LG, HG LG, HG	Enh Enh	*SLC44A2* ↑ *SLC44A2* ↑	−(rs10405617) ATF2;JUN;JUND (rs10948)
rs12910841 rs2290574	T C	TKR	rs4886649	*RPP25*, *ULK3*, *CYP1A1* (rs12910841) *ULK3,RPP25* (rs2290574)	Syn LG, Syn	Pr Pr	*RPP25* ↓ *ULK3* ↓ *CYP1A1* ↓ *ULK3* ↓ *RPP25* ↓	RFX1;RFX5 (rs12910841) ZBTB7B; ZNF675 (rs2290574)
rs17196808	C	ALLOA	rs6012893	*LINC01270*, *PELATON*	Syn	Pr	*LINC01270* ↑ *PELATON* ↑	NFE2L2
rs397891	C	ALLOA	rs645019	*CDK10*, *SPATA2L*	HG	Pr	*CDK10* ↑ *SPATA2L* ↓	RFX2
rs3742566 rs61210954	T T	ALLOA	rs7147106	*LGALS3* (rs3742566) *ATG14*, *WDHD1*, *SOCS4* (rs61210954)	LG, HG Syn	Pr Pr	*LGALS3* ↑ *ATG14* ↑ *WDHD1* ↑ *SOCS4* ↑	−(rs3742566) ESR1 (rs61210954)
rs28529403 rs61764202	T C	KNEE	rs79379134	*MAPK3* *MAPK3*	LG LG	Pr Pr	*MAPK3* ↓	ZNF423 (rs28529403) KLF15;SP1;SP2 (rs61764202)
rs55853698	T	KNEE	rs931794	*PSMA4*, *CHRNA5*	Syn	Pr	*PSMA4* ↓ *CHRNA5* ↑	ZNF93

Regulatory regions were defined using chondrocyte ATAC-seq^39^ and ChIP-seq^86^ data complemented with SCREEN (ENCODE) regulatory annotations^40^. Variant-gene assignments were filtered to include only eGenes supported by independent regulatory evidence, specifically genes listed in the SCREEN regulatory region annotations or genes connected to the variant through chondrocyte Hi-C interactions. Gene names are displayed in italic.

*TF* transcription factor, *Enh* Enhancer, *Pr* Promoter, *ALLOA* Osteoarthritis at any site, *TKR* total knee replacement, *KNEE* knee osteoarthritis, *LG* low-grade cartilage, *HG* high-grade cartilage, *Syn* synovium).

In high-grade cartilage, we identify seven credible set variants overlapping active promoters, three of which are part of promoter-enhancer loops: rs35346340 (*FES*; looping to *FES*/*FURIN*; eGene: FES), rs146098127 (*SMG1P5*; looping to *SMG1P5*/*ENSG00000273724*), and rs397891 (*LINC02166*; looping to *CDK10*/*VPS9D1-AS1*/*SPATA2L*; eGenes include *CDK10* and *SPATA2L*). We further identify eleven variants located in active enhancers, six of which are also connected to promoters through enhancer–promoter loops: rs7187586 (looping to *LMF1;* eGenes: *C1QTNF8, SSTR5, SSTR5-AS1*), rs10405617 and rs10948 (looping to the *SLC44A2*/*AP1M2*/*CDKN2D*/*KRI1* regulatory region; eGene: *SLC44A2*), rs11720984 and rs76439043 (looping to the *ITGA9*/*MYD88*/*OXSR1* region; eGenes: *CTDSPL*, *VILL*), and rs11731421 (looping to *FGFR3*; eGene: *FGFR3*) (Table 1 and Supplementary Data 16).

In synovium, we identify 13 credible set variants overlapping promoters from the SCREEN database^40^. Four of these map to promoters where the SCREEN-annotated gene matches the corresponding eGene: rs12129705 (*ZNF697*), rs137080 (*RRP7A*), rs4932178 (*FES*), and rs55853698 (*PSMA4*). Ten of the 13 promoter variants are predicted to alter transcription factor motifs, potentially modulating promoter regulatory activity (Table 1 and Supplementary Data 16). In fat pad no credible set variant overlapped a promoter region.

For those variants overlapping regulatory regions, we further examined if they are predicted to change the affinity of known transcription factor binding sites^23^ and the concordance of their effect allele with the GWAS risk-increasing allele, enabling us to point to putative mechanisms. In total, we find 19 credible set variants across tissues affecting the binding motif of 38 transcription factors (Supplementary Data 16).

Among notable examples, we find rs10948, a variant overlapping an active enhancer regulating the *SLC44A2* gene, which has been previously described by Bittner et al.^28^. The T allele of rs10948 is associated with decreased *SLC44A2* expression in both low- and high-grade cartilage (eQTL) and it is also predicted to decrease the affinity of ATF2/JUN/JUND (AP-1 complex) transcription factor binding. The T allele is additionally protective against knee osteoarthritis risk. Collectively, these findings support a model in which the T allele attenuates AP-1-dependent enhancer activity, resulting in reduced *SLC44A2* transcription. The protective effect of this allele implies that lower *SLC44A2* expression exerts a functional benefit in cartilage, thereby decreasing osteoarthritis susceptibility.

We further find two credible set variants (rs61764202, rs28529403) overlapping the active promoter of *MAPK3* and affecting affinity of several transcription factor binding (including: KLF15, SP1, SP2, and ZNF423, respectively). For rs61764202 the risk allele (C) for knee osteoarthritis decreases expression of *MAPK3* is predicted to decrease the affinity for KLF15, SP1, and SP2 transcription factor binding, while for rs28529403, the risk allele (T) decreases expression of *MAPK3* and is predicted to increase the affinity for ZNF423 (which has been shown to act as both activator and repressor of transcription). These results indicate that *MAPK3* sits in a promoter hub where distinct risk alleles at nearby variants differentially affect transcription factor binding and gene expression.

We further obtained mechanistic insight for rs35346340, which overlaps an active promoter in synovium and a promoter-enhancer loop in both low- and high-grade cartilage. The osteoarthritis risk allele (G) is associated with increased *FES* expression in all tissues and additionally with decreased expression of *FURIN* in synovium. This variant overlaps several transcription factor motifs, including sites for TWIST1, KLF15, ZNF281, ZNF320, and ZNF530. Motif analyses indicated that the protective allele (C) is predicted to decrease binding affinity for several of these TFs, particularly TWIST1, KLF15, ZNF281, and ZNF320, consistent with reduced promoter activity and lower *FES* transcription. The looped region in cartilage also contacts the neighboring *FURIN*, indicating that rs35346340 sits within a broader regulatory region.

Lastly, we find two credible set variants overlapping regulatory regions for *WWP2* (rs10048090 and rs2102066). *WWP2* is an established effector gene for osteoarthritis^6^ with GWAS signals colocalizing with both methylation and splicing QTLs affecting *WWP2*^22,41,42^. Rs10048090 lies in an active enhancer that forms a Hi-C loop to the *WWP2* promoter, while rs2102066 resides in its active promoter that is also involved in a HiC loop. In low-grade cartilage, the osteoarthritis risk allele (A) of rs10048090 and the risk allele (A) of rs2102066 are associated with increased *WWP2* expression. Although none of these variants are predicted to disrupt any identifiable transcription factor motifs, their location within looped enhancers and promoters that regulate *WWP2* suggests a potential mechanism of action of this signal and *WWP2* on osteoarthritis.

### Translational drug repurposing opportunities for the colocalizing genes

Considering the translational potential of drug targets with genetic evidence supporting their effect on disease^43^, we conducted a druggability search on the 136 colocalizing genes using the Open Targets database^21^. A total of 14/136 genes are linked to drugs with at least phase II clinical trials completed (Supplementary Data 17). *TGFA* is the only colocalizing gene targeted by a drug with phase II clinical trials for osteoarthritis completed. *TGFA* is the target of fepixnebart, an antibody that inhibits TGFA interaction with its receptor, thereby hindering downstream signaling pathways. In addition to osteoarthritis, fepixnebart is under clinical trial with phase II completed for neuropathic pain and low back pain.

By overlaying the mechanism of action of drugs targeting colocalizing genes with the direction of causal effect on osteoarthritis risk identified through the MR analysis, we highlight potential drug repurposing opportunities. Seven colocalizing genes, targeted by drugs with at least phase II clinical trials completed but have not been investigated for osteoarthritis, show mechanisms of action that align with the genetically inferred direction of effect: *ALAS1, FRK, LGALS3, PIK3R3, SMAD7, SSTR5*, and *KCNK3* (Supplementary Data 17). We have shown genetic evidence linking *SMAD7* and osteoarthritis risk. Antisense oligonucleotide therapies targeting *SMAD7*, such as mongersen, have been developed to enhance TGF-β signaling in inflammatory disorders like Crohn’s disease, and could offer a pharmacological precedent for exploring this pathway in osteoarthritis.

*LGALS3*, encoding galectin-3, is involved in inflammation, fibrosis and tissue remodeling and has been previously implicated as an effector gene for osteoarthritis^6^. Our results show evidence of a risk-increasing effect of increased *LGALS3* levels in cartilage on osteoarthritis at any site (high-grade: OR = 1.04, 95% CI 1.03–1.05, *q*-value = 1.19 × 10⁻¹³; low-grade: OR = 1.03, 95% CI 1.03–1.04, *q*-value = 2.05 × 10⁻¹³). Galectin-3 is implicated in knee osteoarthritis progression with patients exhibiting higher plasma and synovial fluid galectin-3, especially with advanced disease^44^. Pharmacological galectin-3 inhibitors such as belapectin, currently in clinical trials for fibrotic diseases such as nonalcoholic steatohepatitis^45^, align with the direction of our genetic findings and could potentially represent actionable candidates for osteoarthritis repurposing.

*FRK* encodes a non-receptor tyrosine kinase implicated in cellular proliferation and differentiation. We identify a risk-increasing effect of elevated *FRK* expression in high-grade cartilage on osteoarthritis (OR = 1.05, 95% CI 1.02–1.08, *q*-value = 6.84 × 10⁻⁴). Several multi-kinase inhibitors approved for cancers, including dasatinib and regorafenib, exhibit inhibitory activity against *FRK*, providing a potential pharmacological framework for therapeutic repurposing. Mechanistically, tyrosine kinase signaling has been implicated in chondrocyte hypertrophy, ECM degradation, and cartilage catabolism^46^.

Two colocalizing genes within the fibroblast growth factor signaling pathways, *FGF1* and *FGFR3*, are targeted by drugs that have completed phase II clinical trials. *FGF1* has been associated with osteoarthritis through its role in cartilage degradation and repair mechanisms. Studies suggest that inhibiting *FGF1* may contribute to cartilage repair by promoting chondrocyte proliferation and ECM synthesis while also modulating inflammation^47,48^. Notably, both suramin and muparfostat, both approved for cancer indications, inhibit *FGF1* activity and may have potential in modulating osteoarthritis-related mechanisms. In contrast, *FGFR3* encodes a receptor tyrosine kinase that mediates cell growth, differentiation, and maintenance of bone and cartilage tissues via ossification^47^. Experimental studies in mouse models show that loss of *FGFR3* function in chondrocytes leads to worsened cartilage degeneration and increased osteoarthritis severity^49^. Multiple FGFR3-targeting drugs, including ferdafitinib and pemigatinib, are approved for cancers. However, inhibition runs counter to the therapeutic strategy suggested by recent findings, which indicate that FGFR3 activation is potentially protective against osteoarthritis progression by promoting cartilage homeostasis^47^.

## Discussion

Large efforts have been made to identify genetic risk signals for osteoarthritis. However, elucidating the function and tissue of action of these variants is challenging. Here, we have generated the largest *cis*- and *trans*-eQTL maps in low-grade cartilage (*n* = 261), high-grade cartilage (*n* = 212), synovium (*n* = 277) and fat pad (*n* = 92) knee osteoarthritis joint tissues. By focusing on primary tissues directly affected by osteoarthritis, the identified *cis-*eQTLs provide a more precise and context-specific understanding of the genetic architecture underlying the disease. This resource enables the dissection of genetic regulation in key disease relevant tissues and highlights the distinct genetic regulatory mechanisms operating in osteoarthritis-affected tissues.

Building on this foundation, we integrated the newly-generated eQTL maps with osteoarthritis GWAS and functional data from a range of disease-tissue-specific and publicly available resources, we prioritize effector genes and highlight the putative mechanism of action of underlying risk loci. For these effector genes, we provide tissue- and stage-resolved direction of effect, informing whether risk alleles are associated with increased or decreased expression of the implicated genes. Overlaying credible set variants with joint tissue regulatory annotations, chromatin interaction maps, and predicted effects on transcription factor binding motifs further strengthens the identified variant to gene regulation connections.

To benchmark our maps against prior work, we compared our results with the first *cis-*eQTL study in cartilage and synovium^14^, which constitutes a subset of our samples. We recapitulate ~70% of *cis-*eQTL-gene pairs across tissues with largely concordant effect sizes. The comparison of our study with the synovium *cis-*eQTL map in 201 osteoarthritis patients of Han Chinese ancestry^16^ yielded a 54% overlap of eGenes. This may be attributed to population-specific effects, including differences in allele frequencies and linkage disequilibrium patterns, and/or to analysis approach differences.

We identify genetic regulatory effects that vary with cartilage degeneration and nominate genes for which these grade-specific eQTLs are likely to contribute to expression differences between disease stages. These context-dependent effects, which would not have been captured by stage agnostic analyses, indicate that genetic regulation of transcription can shift as cartilage pathology progresses and may therefore point to mechanisms relevant to disease progression. However, inferring true disease progression eQTLs remains challenging, given the complex, heterogeneous nature of osteoarthritis and the fact that disease progression is also driven by a combination of environmental and genetic factors.

Colocalization between the *cis*-eQTL maps and knee-related osteoarthritis GWAS finds evidence of a shared signal for 34.1% of the tested loci, yielding 136 unique colocalizing genes. The partial elucidation of GWAS signals by colocalization with eQTL data underscores the increasing evidence that eQTLs and GWAS identify partially different sets of associated variants partly due to natural selection^50^.

We prioritized 45 colocalizing genes supported by multiple lines of evidence of involvement in osteoarthritis, with 40 being causally linked to knee-related osteoarthritis risk. Considering that genetic evidence support can accelerate drug discovery^50^, the prioritized genes represent appealing candidates for clinical translation, including drug repurposing opportunities. For instance, we highlight the galectin-3 inhibitor drugs belapectin, targeting *LGALS3*, and mongersen, targeting *SMAD7*, as a potential drug repurposing opportunity for osteoarthritis treatment. Both drugs are indicated for chronic diseases, being safe for long-term use.

Despite the gain in power to elucidate GWAS signals presented in this study, the generated data are restricted to samples from individuals of European ancestry. This reflects a well-recognized gap in the availability of molecular data from global populations^51^. Going forward, it is important that efforts focus on closing this gap. We also note substantial differences in sample size across tissues, particularly for fat pad, which limits direct comparability of discovery rates - a limitation we partially mitigated using multi-tissue comparison methods such as mashr^18^ which models shared effects across tissues. Our tissue panel does not exhaust all osteoarthritis-relevant tissues, so regulatory effects operating in other joint tissues may be missed. In relation to that, our data do not establish tissue specificity in a strict sense, as comparisons with external resources such as GTEx are confounded by differences in disease state, and no comparable osteoarthritis eQTL datasets are available across a broader panel of primary tissues. In addition, transcriptomes are dynamic, therefore some regulatory effects may be time-specific and not captured in the tissues and conditions analysed here. Future work complemented by time-course or perturbation experiments in relevant joint cell models, will help resolve these effects. The prioritized effector genes and their associated risk loci provide testable hypotheses that should be refined through targeted functional assays such as CRISPRi or CRISPRa and allele-specific reporter experiments. Recent single-cell RNA-seq (scRNA-seq) studies in osteoarthritis cartilage^52,53^, synovium^54^, and fat pad^55,56^ have implicated specific cell types involved in osteoarthritis. Furthermore, *trans-*eQTLs have been shown to capture cell-type-specific effects^57^. Combining genomic data with scRNA-seq in larger sample sizes is needed to distinguish true regulatory effects from differences in cell composition.

Robust molecular data from primary tissues can be valuable in enhancing the translation of genetic findings for all complex diseases. Here, we have generated the largest molecular eQTL maps for four osteoarthritis-relevant tissues and two cartilage disease stages. Leveraging these powerful data, we point to key biological mechanisms underlying knee osteoarthritis. Our findings identify candidate effector genes for this disease along with tissue of action and direction of effect, pointing to drug repurposing opportunities.

## Methods

We hereby confirm that this study complies with the relevant ethical regulations, which are the following:

- Tissue from patients undergoing total knee replacement collected at the University of Sheffield for RNA Sequencing and Whole-Genome Sequencing: Human Tissue Authority license 12182 and National Research Ethics Service approval 15/SC/0132 and 20/SC/0144, South Yorkshire and North Derbyshire Musculoskeletal Biobank, University of Sheffield, UK.
- Tissue from patients undergoing joint replacement collected at the Tartu University Hospital for Spatial Transcriptomics: Research Ethics Committee of the University of Tartu (379/M-10, 12th of June 2023)

We also confirm that the study design and conduct complied with all relevant regulations regarding the use of human study participants and was conducted in accordance to the criteria set by the Declaration of Helsinki (National Research Ethics Service, South Yorkshire and North Derbyshire Musculoskeletal Biobank, University of Sheffield, UK and Research Ethics Committee of the University of Tartu) and that the use of the datasets was approved by the local ethics committee at the Technical University Munich.

### Sample selection

In this study, we included osteoarthritis patients who had undergone total knee replacement surgery for osteoarthritis with no history of significant knee surgery, infection, fracture, or malignancy within the previous 5 years. Additionally, we ensured that no patient had been treated with corticosteroids (systemic or intra-articular) within the previous 6 months or any other drug associated with immune modulation. We isolated matched cartilage samples from weight-bearing regions of the joint, along with synovium and fat pad tissue from the same patients. Cartilage samples were also scored macroscopically using the International Cartilage Repair Society (ICRS) scoring system^58^. From each patient, we collected one sample of ICRS grade 0 or 1, representing low-grade osteoarthritis degradation, and one sample of ICRS grade 3 or 4, representing high-grade osteoarthritis degradation. All study participants provided informed consent and samples were collected under Human Tissue Authority license 12182 and National Research Ethics Service approval 15/SC/0132 and 20/SC/0144, South Yorkshire and North Derbyshire Musculoskeletal Biobank, University of Sheffield, UK.

Of the 320 patients included in the final eQTL analyses, 176 (55.0%) were female, and 144 (45.0%) were male, with a mean age of 70.5 years (SD 8.86, range 38–93) (Supplementary Data 1). Biological sex was recorded from clinical records and verified genetically during whole-genome sequencing quality control. Sex was included as a covariate in all eQTL models, and eQTL mapping was restricted to autosomes; no sex-stratified analyses were performed, as the per-tissue sample sizes were not suited to well-powered sex-stratified discovery. Gender was not separately recorded. Study participants were not compensated for their participation.

### Chondrocyte, synoviocyte and adipocyte isolation

Primary tissues were transported from surgery in PBS and processed immediately into serum-free medium consisting of DMEM/F-12 (1:1; Life Technologies) supplemented with 2 mM glutamine (Life Technologies), 100 U/ml penicillin and 100 μg/ml streptomycin (Life Technologies), 2.5 μg/ml amphotericin B (Sigma-Aldrich), and 50 μg/ml ascorbic acid (Sigma-Aldrich).

For chondrocyte isolation, cartilage was separated from the underlying bone, dissected, and washed twice in 1× PBS, then digested overnight at 37 °C in serum-free medium containing 3 mg/ml collagenase type I (Sigma-Aldrich) on a shaker. The cell suspension was filtered through a 70 μm cell strainer (Fisher Scientific) and centrifuged at 400 × *g* for 10 min. The pellet was washed twice in serum-free medium with centrifugation at 400 × *g* for 10 min between washes.

For synoviocyte isolation, the synovial membrane was dissected from the underlying tissue, trypsinised for 1 h, then digested overnight at 37 °C in serum-free medium containing 1 mg/ml Collagenase Blend H (Sigma-Aldrich) on an orbital shaker on a gentle setting. The cell suspension was filtered through a 100 μm cell strainer (Fisher Scientific) and centrifuged at 400 × *g* for 10 min. The pellet was washed twice in serum-free medium with centrifugation at 400 × *g* for 10 min between washes.

Fat pad tissue was processed in the same way as synovium, with centrifugation at 400 × *g*.

For all tissues, cell number was determined using a haemocytometer and viability assessed by trypan blue exclusion (Invitrogen).

### Library preparation

#### RNA-sequencing

We extracted RNA using Qiagen AllPrep RNA Mini Kit according to the manufacturer’s instructions^14,19^. We isolated and sequenced samples in five batches that were later uniformly processed. We isolated Poly-A-tailed RNA from total RNA using Illumina’s TruSeq RNA Sample Prep v2 for batches 1–4, while for the 5th batch, we used SMART-Seq® Ultra® Low Input RNA Kit. The libraries were then sequenced on the Illumina HiSeq 2000 and HiSeq 4000 (75 bp paired-ends) (batches 1–4) and Novaseq 6000 (batch 5), recovering a median of 80.8 million reads per sample.

#### Whole-genome sequencing (WGS)

WGS data were generated from patients’ DNA^59^. The libraries were prepared from DNA samples using the standard Illumina paired-end DNA protocol as per manufacturers’ instructions. Then, they were amplified and sequenced in two batches on the NovaSeq platform.

### Preprocessing and quality control (QC) of RNA-sequencing data

We performed alignment of the fastq files to the reference genome Ensembl GRCh38 release 105 (cDNA and non-coding) using STAR version 2.7.9a^60^. We summarized the reads using FeatureCounts (Subread 2.0.3) software and called duplicates using PicardTools (v2.18.25) [https://broadinstitute.github.io/picard/]. We further used RSeqQC (version 4.0.0)^61^ to summarize alignment metrics. We first performed sample-level quality control and excluded samples that did not pass the following criteria: RNA integrity number >5, percentage of uniquely mapped reads >70%, reads mapping to genomic features >40%, reads mapping to intronic regions or rRNA <30%, reads that failed RseQC strandness check <30% and including >10 million reads. Using the aforementioned criteria, we excluded 42 low-grade cartilage samples, 35 high-grade cartilage samples, 14 synovium samples, and 13 fat pad adipocyte samples. This way, we ensured the inclusion of only high-quality samples.

We further excluded outlier samples in a tissue-specific manner. Outliers were identified using robust principal component analysis, considering the first three principal components (PcaGrid method rrcov R package)^62^. We excluded samples from individuals that had bilateral knee replacement, where low- and high-grade disease cartilage samples were pooled (seven individuals). For individuals with bilateral knee replacement, we excluded one pair of matched cartilage samples each (three samples), keeping only the sample pair with the best quality. For samples with technical replicates, we also kept the one with the best quality (two samples). The final number of samples after the exclusions across tissue was 881, derived from 331 patients (low-grade cartilage: 272, high-grade cartilage: 223, synovium: 288, fat pad: 98). For each tissue, we filtered lowly expressed genes using the *filterByExpr* function from edgeR (v3.38.4)^63^ using default parameters, retaining genes that were expressed with more than 10 counts for 70% of tissue samples. The CPM cutoff for each tissue under default parameters was 0.292 for cartilage, 0.267 for synovium, and 0.279 for infrapatellar fat pad. After QC, we performed principal component analysis (PCA) to find main drivers of variation and evaluate the presence of batch effects (Supplementary Fig. 6). The principal component 1 (PC1), explaining 35.64% of the variation in the data, corresponded to the different tissues, while PC2, explaining 12.12% of the variation, corresponded to the sequencing batches.

### Preprocessing and QC of whole-genome sequencing (WGS) data

For WGS preprocessing and QC CRAM files were converted to BAM format using samtools (version samtools conda version 1.14). The latter were further converted to fastq format using *bedtools bamtofastq* function (bedtools conda version 2.30.0). We performed variant calling per batch and using Sarek nf-core pipeline (version 2.7.1, https://nf-co.re/sarek/2.7.1) with the additional options “*–tools HaplotypeCaller, –generate_gvcf”*. We further performed joint variant calling by adapting the GATK4 pipeline (https://github.com/IARCbioinfo/gatk4-GenotypeGVCFs-nf) to be used with GATK (docker container broadinstitute/gatk:4.2.5.0)^64^. For all variant calling purposes GRCh38 reference was specified.

For variant-level quality control, we used Variant Quality Score Recalibration method specifying a tranche threshold of 99.5% for SNPs and 99% for INDELS. The expected false positive rate for SNPs was 2.5% and the expected sensitivity was 97%.

We excluded samples with high heterozygosity rate (two samples), non-reference allele concordance with the directly typed genotype data using variants MAF > 0.01 (one sample—also showing heterozygosity rate), outliers in sequencing depth (one sample), sex mismatched (two samples), relatedness > 0.2 (two samples), ethnicity outliers (two samples). Ethnicity outliers (three individuals) were identified using the Ancestry and kinship toolkit (based on 1000 G data from phase three; https://github.com/Illumina/akt/tree/master) (akt, v0.3.3)^65^. Nine samples were excluded in total.

We further evaluated the concordance between genotypes and RNA-seq using VerifyBamID (v1.13). We assessed sample mislabeling, swaps, and cross-sample contamination. For each RNA-seq BAM, VerifyBamID^66^ was run against the joint WGS genotype VCF to infer the most likely matching donor (BEST) and to estimate mixture/contamination metrics (FREEMIX and CHIPMIX). Expected donor identities were specified a priori via a sample-to-donor mapping file, and concordance was evaluated by comparing BEST to the expected donor. Based on this, we corrected two mislabeled samples and one complete swap between two donors’ synovium samples. We also excluded three redundant samples where the same donors were already represented by correctly assigned tissues. To control for contamination, samples with FREEMIX and/or CHIPMIX > 0.2 after swap/mislabel correction were excluded (six samples), consistent with thresholds used in prior studies^67^.

After sample-level QC, we performed additional filtering and excluded variants with MAF < 0.01, Hardy–Weinberg equilibrium *P* < 10^−5^, and call rate ≤ 0.99. After selecting individuals matching WGS and RNA-seq data per tissue we performed further variant filtering to keep only bi-allelic and common variants (MAF > 0.05). The final WGS data sets per tissue after filtering were as follows: low-grade cartilage (261 individuals, 6,291,967 variants), high-grade cartilage (212 individuals, 6,291,969 variants), synovium: (277 individuals, 6,338,438 variants), fat pad (92 individuals, 6,258,186 variants).

To characterize population structure and ensure consistency across datasets, we next performed principal component analysis (PCA) on the filtered genotype data using bcftools (v1.21)^68^, PLINK (v1.9)^69^, and the 1000 Genomes Project reference panel^65^. Genotype data in VCF format were filtered to retain only unique samples across all tissues using *bcftools view* and updating (MAF ≥ 0.05), call rate (CR ≥ 99%), and biallelic status as described above. The resulting VCF contained 6,347,758 variants across 320 samples. The resulting VCF was indexed and converted to PLINK binary format. To assess population structure, we downloaded phased genotype data from the 1000 Genomes Project (GRCh38), normalized and converted it to PLINK format, and LD-pruned each chromosome. We merged the pruned reference data across chromosomes and harmonized variant IDs with our dataset to identify common variants with 1000 Genomes Project reference panel (205,409 variants). PLINK was used to generate a merged dataset and compute principal components, which were used as covariates in downstream analyses. To generate PCA plots, we read eigenvectors and eigenvalues from PLINK output in R and calculated the proportion and cumulative variance explained by each principal component (PC). We annotated samples by merging with 1000 Genomes population metadata and labeled study samples accordingly (Supplementary Fig. 7). We also evaluated genetic structure and potential genotype outliers by also performing PCA separately on the eQTL cohort WGS genotypes using PLINK^69^. PC1 was collinear with the WGS sequencing batch so it was excluded to avoid introducing multicollinearity. PC2–PC5 were therefore included as genotype-structure covariates in the eQTL analyses.

### Power analysis

To contextualize the eQTL results and to inform future study design, we performed a power analysis to estimate the expected power to detect *cis*-eQTLs given the available sample sizes. Power analysis for the eQTL analysis was performed using the R package powerEQTL (v0.3.4)^70^ (*powerEQTL.SLR*) across different MAF, slopes, and sample sizes (Supplementary Fig. 1).

### Mapping *cis*-eQTL

For the eQTL analysis, we included WGS data and matched RNA-seq tissue-specific expression data from the same patients (*n* = 320 osteoarthritis patients across all tissues). For eQTL analyses, the gene expression count matrix was normalized using the trimmed mean of *M*-values normalization^71^ (between samples normalization) and then inverse-normal transformed (across genes normalization). This procedure was performed separately per tissue (cartilage, synovium, and fat pad). For eQTL analyses, we included protein-coding and long non-coding RNA (lncRNA) genes, excluding other gene biotypes due to higher quantification uncertainty. We further included genes on the autosomes. The total number of genes per tissue used in eQTL testing was formed as follows: cartilage (both low-grade and high-grade): 15,476, synovium: 14,482, fat pad: 14,635. We further defined the transcription start-site (TSS) for each gene based on its most abundant transcript across the tested tissues (transcript abundance quantified using RSEM (v1.3.3)^72^). The resulting matrices were then used to estimate PEER factors^73^ using peer (v1.0) representing hidden data variation.

To maximize *cis*-eGene discovery, we optimized the number of PEER factors separately for each tissue while accounting for known covariates (sex, age, and RNA-seq sequencing batch) by providing them to the PEER model via PEER_setCovariates (Stegle et al., Nature Protocols, 2012)^74^. For each tissue, we performed *cis*-eQTL mapping across a grid of PEER factor counts and quantified the number of significant *cis*-eGenes detected at each setting. We selected the tissue-specific optimum as the first value at which eGene discovery plateaued or began to decline in agreement with GTEx^75^ recommendations (Supplementary Fig. 8). The selected number of PEER factors included per tissue in the eQTL model is as follows: low-grade cartilage: 55, high-grade cartilage: 40, synovium: 70, fat pad: 12) We further verified that the inferred latent PEER factors were not strongly associated with the known covariates (ANOVA for categorical covariates and linear regression for continuous covariates).

We conducted *cis*-eQTL analysis per tissue using tensorQTL (version 1.0.9)^76^ following the GTEx pipeline [https://github.com/broadinstitute/gtex-pipeline/tree/master/qtl]. This method uses linear regression to test for associations between variant–gene pairs in a specified window (1-Mb specified for *cis-*eQTL) within the transcription start site of a gene (nominal pass). The nominal *p*-values were estimated for every variant–gene pair with the following model:

$${Expression}\, 	 \sim \, {genotype}\,+\,{age}\,+\,{sex}\,+\,{WGS}{\_}{sequencing}{\_}{batch}\, \\ 	+{Genotype}[{PC}2-{PC}5]\,+{\#}{PEER}{\_}\,{factors}$$

Out of 320 WGS samples, 246 were extracted in the first batch and 74 in the second sequencing batch. The number (#PEER_factors) was determined per tissue as described above.

To test for association between gene expression and the lead variant (top in -*cis*), we used tensorQTL with the implemented permutation scheme specifying 1000 permutations per gene. This way, we calculated the nominal *p*-value threshold per gene and the gene-level *q*-values corrected for multiple testing (Storey-Tibshirani). To identify eGenes, we performed *q*-value correction of the permutation *p*-values for the lead-associated variant per gene at a threshold of 0.05. To identify significant eQTL-gene pairs, we filtered the nominal output for the pairs below the gene-specific *p*-value threshold. We further identified conditionally independent eQTLs using a forward-backward stepwise regression procedure. In brief, for each eGene, the lead variant was added as a covariate and the *cis*-window re-tested to detect additional independent signals, iterating until no further variant passed the gene-level significance threshold; a backward step then re-tested each signal conditional on the others to confirm independence. This procedure follows the approach implemented in GTEx^17^.

### Mapping *trans*-eQTL

We performed *trans-*eQTL analysis per tissue using MatrixeQTL (v2.3)^77^. Expression and genotype input data were prepared as described for the *cis-*eQTL analysis. The same covariates and number of PEER factors were included in the final model. We performed *trans-*eQTL analysis on all variants whose distance to the genes’s TSS was more than 5 Mb. Given the large number of tests, only gene-SNP pairs with *p*-value < 10^−6^ were retained. Similar to the *cis*-eQTL analysis, and as previously reported^17^, we calculated gene-level significance as follows:

- For each gene, we selected the most significant *trans*-eQTL (i.e., SNP-gene pair with the lowest nominal *p*-value) across chromosomes 1–22.
- The nominal *p-*values were multiplied by 10^6^ to correct for the number of expected independent tests for a genome-wide association analysis at MAF 5% in a European population. Corrected *p*-values were capped at 1.
- FDR correction on the list of corrected *p*-values was performed using the *q*-value package (v2.28.0). *Trans*-eQTLs were defined as those with *q*-value < 0.05.
- To identify per-gene *trans*-eQTLs, a genome-wide significance threshold was calculated. We identified the largest *p*-value among significant *trans*-eQTLs (*q*-value ≤ 0.05) and the smallest *p*-value among non-significant *trans*-eQTLs (*q*-value > 0.05). We then took the midpoint between these two values to define a conservative genome-wide *p*-value threshold for significance at FDR 5%.

Finally, we removed potential false positive *trans*-eQTLs caused by read cross-mapping. To this end, we utilized publicly available cross-mappability resources for the GRCh38 reference genome and excluded all *trans-*eQTL-gene pairs that had a mappability score less than 0.8^78^. We identified conditionally independent *trans*-eQTLs for each analyzed tissue by clumping the significant *trans*-eQTLs based on LD. We used PLINK^71^ to perform clumping on a window of 1 Mb and a strict LD threshold *r*^2^ < 0.001.

### Comparison with existing *cis-*eQTL maps

To find newly reported associations, we compared the significant eQTL-gene pairs with published eQTL studies from refs. ^14,16^ Both of these studies report eQTL associations on reference genome build 37, while our eQTL maps are on build 38. To establish a fair comparison, we converted the variant positions to the corresponding reference SNP cluster ID (rsID) as rsIDs do not depend on a reference genome. We mapped variants’ genomic positions to rsIDs using dbSNP database^79^ available through Bioconductor package SNPlocs.Hsapiens.dbSNP144.GRCh37. Replication was assessed by intersecting the significant variant to gene pairs reported in each external study with the significant variant to gene pairs identified in our dataset, and quantifying the proportion that overlapped.

We further evaluated the association of effect sizes (slopes) for *cis-*eQTLs by conducting a linear regression analysis of common eQTL effect sizes between our study and that of ref. ^14^, which was performed in a subset of individuals from our study, all of European ancestry (Supplementary Fig. 2). Specifically, we analyzed 50,788 common *cis-*eQTLs with rsIDs in synovium, 63,274 in low-grade cartilage, and 80,625 in high-grade cartilage. For each tissue type, we fit a linear regression model with the effect sizes from our study as the predictor variable and those from ref. ^14^ as the outcome variable. From this model, we extracted the R-squared value to quantify the strength of association and the *p*-value to evaluate statistical significance. To visualize these associations, we used ggplot2 (v3.5.0)^80^ to generate scatter plots with fitted regression lines and confidence intervals for each tissue type.

### Identification of disease tissue-sharing and single-tissue eQTLs

To assess eQTL patterns that are unique or shared between the tested disease tissues, we first explored eQTL effects detection in only one tissue due to being expressed only in a single disease tissue (i.e., eQTLs in genes not common across tissues). To formally assess the presence of unique eQTL effects for genes that are commonly expressed in all disease tissues, we applied multivariate adaptive shrinkage using mashr (v0.2.79)^18^ on the top *cis*-eQTLs for each gene across four tissues, focusing on genes expressed in all tissues (*n* = 13,181). We input the estimated effect sizes and their associated standard errors, along with 59,310 randomly selected SNP-gene pairs tested across all tissues, to fit the Mash model as previously done (https://github.com/stephenslab/gtex-eqtls). mashr’s output of effect size estimates and local false sign rates (LFSR) were used as measures of *cis*-eQTL magnitude and significance, respectively. We set an LFSR threshold of <0.05 to identify significant eQTLs within each tissue. Shared *cis*-eQTLs were defined as those with an LFSR < 0.05 and an effect size within a twofold range of the strongest effect observed across tissues. To identify *cis*-eQTLs that are enriched for a single disease tissue compared to the rest of tested disease tissues, we implemented a filtering procedure that required: (1) LFSR < 0.05 in a single disease tissue; (2) LFSR ≥ 0.1 in all other tissues; (3) *a* ≥ twofold larger absolute effect size in the single tissue compared to all others; and (4) a minimum absolute effect size of 0.1. This ensured eQTLs with unique effects in one osteoarthritis tissue.

### Identification of cartilage-grade specific eQTLs

To identify cartilage grade specific eQTLs, we applied the same mashr^18^ based framework as for tissue sharing, but restricted to genes expressed in both low- and high-grade cartilage (*n* = 15,476), and defined an eQTL as grade-specific if it met LFSR < 0.05 in one grade, LFSR ≥ 0.1 in the other grade, had at least a twofold larger absolute effect size in the specific grade, and an absolute effect size of at least 0.1. To relate grade-specific genetic regulation to cartilage degeneration, we additionally queried the eGenes that were differentially regulated against genes differentially expressed^19^ between low and high-grade cartilage, defining differentially expressed eGenes (DE-eGenes).

### eQTL variant annotation

We annotated all variants tested in eQTL analyses for functional impact on known genes using the ANNOVAR tool (v2025-07-29) (https://annovar.openbioinformatics.org/en/latest/)^81^. Regulatory annotations were drawn from the Ensembl Regulatory Build^82^, covering features such as enhancers, promoters, promoter-flanking regions, transcription factor binding sites, CTCF binding sites, and open chromatin regions. For low- and high-grade cartilage tissue, we further utilized enhancer-promoter loop coordinates specific to osteoarthritis cartilage, as mapped by chromatin accessibility Hi-C data from ref. ^28^. From this Hi-C map (hg38 build), we extracted genomic coordinates for: (1) chondrocyte-specific CTCF binding sites, marking the most active regions within topologically associated domains; and (2) active chondrocyte promoters and enhancers within Hi-C loops. Active loops were identified using ATAC-seq open chromatin data combined with ChIP-seq peaks for H3K4me1 (enhancers), H3K4me3 (promoters), and H3K27ac (activity signal for both) signals. Supplementary Fig. 3 shows the distribution of functional annotations for *cis* and *trans*-eQTL variants, summarizing their overlap with different genomic categories.

### Genetic colocalization of *cis*-eQTL with GWAS signals

We conducted regional genetic colocalization analyses between eQTL from osteoarthritis primary tissues (low-grade and high-grade cartilage, synovium, and fat pad) and three knee-related osteoarthritis GWAS (knee osteoarthritis - KNEEOA, total knee replacement - TKR, and osteoarthritis at any site - ALLOA)^6^. Regions to run genetic colocalization were defined as 1 Mb windows on either side of the phenotype-specific osteoarthritis GWAS risk signals defined in ref. ^6^. Colocalization analysis was run in the GWAS risk regions that had at least one study-wise significant eQTL. To perform the analyses, we have used the *coloc.abf* function of the *coloc* R package (version 5.2.2)^83^. To overcome possible LD challenges due to for instance unmatched population structure, evidence of colocalization was set at a posterior probability of a shared causal variant (PP4) ≥ 0.8 or PP4 > 0.6 and PP4/PP3 > 2, where PP3 denotes the posterior probability of both traits having a distinct causal variant in the genomic region. For each colocalized genomic locus, we calculated a 95% credible set for the shared causal variant by taking the cumulative sum of the variants’ posterior probabilities to be causal conditional on H4 being true. LD between the single-nucleotide polymorphisms (SNPs) was calculated using PLINK (v.2.0 alpha) based on the UK biobank and was used for visualizing the results in regional association plots^84^.

In regions where no evidence of colocalization but slight evidence of shared causal association between both traits (PP4 > 0.25) was found, we conducted additional analyses using the *coloc.susie* function, which relaxes the assumption of a single shared causal variant by fine-mapping each trait and defining multiple credible sets to run colocalization^85^. For this additional analysis, an LD reference panel is required for each investigated trait. For the eQTL dataset, we used the in-sample LD map, and for the osteoarthritis GWAS, we used the UK Biobank genotype files to derive an LD reference map^84^. We defined evidence of colocalization as for colo.abf(): PP4 ≥ 0.8 or PP4 > 0.6 and PP4/PP3 > 2. Given the lack of credible set information available in coloc.susie, we defined the lead signals from the GWAS and the eQTL as candidates for the lead colocalizing variant.

### Overlap between variants in the 95% credible set of the colocalization with functional data (Hi-C, ATAC-seq, SCREEN)

To prioritize colocalized variants from our analysis, we integrated the 95% credible set variants from coloc.abf() results as well as all lead signals colocalizing in coloc.susie() with data capturing putative regulatory relationships. In particular, for low-and high-grade cartilage, we used 3-dimensional spatial organization data of chondrocytes generated in an earlier study^28^, ATAC-seq^39^, and Histone mark data (H3K4me1, H3K4me3, and H3K27ac)^86^ to identify variants which reside in active enhancers and promoters in chondrocytes that show physical interaction. For synovium and fat pad, given the lack of publicly available matching datasets, we restricted this step to variants overlapping promoters, as annotated in the SCREEN database^40^.

Enhancers and promoters were selected based on annotations from the latest ENCODE SCREEN data release (V4). First, all regions labelled as enhancers (pELS for proximal enhancers and dELS for distal enhancers) and promoters (pELS) were extracted and overlaid with ATAC-seq regions for low-grade (outer region of lateral tibial plateau -oLT region) or high-grade cartilage (inner region of medial tibial plateau -imT region). Valid ATAC-seq peaks used in our analysis were defined as peak regions identified in six out of eight patient samples. These regions in an open chromatin state were further overlaid with regions binding to a combination of H3K4me1 and H3K27ac for enhancers and H3K4me3 and H3K27ac for promoters, respectively. Only regions found in two out of three replicates with these histone marks were labelled as active.

For promoters with colocalized variants, the gene which is annotated in the SCREEN^40^ database was assigned as the target gene. For variants in active enhancer regions, those genes with active promoters in the loop anchor region being in contact with the loop anchor region of the active enhancer were annotated as target genes.

### Transcription factor binding motif analysis

To assess whether *trans*-eQTLs or 95% credible set variants affect transcription factor binding motifs (TFBMs), we used the Bioconductor package motifbreakR^23^ (version 2.20). We filtered the TF motif database to include only human motifs (organism = “Hsapiens”) and restricted it to high-confidence data sources: HOCOMOCOv11-core-A, jaspar2022, and SwissRegulon. We ran motifbreakR() using the following parameters: filterp = TRUE, method = “ic” (information content), threshold = 1e-5, and a uniform background nucleotide distribution bkg = c(A = 0.25, C = 0.25, G = 0.25, T = 0.25). *P*-values for reference and alternative alleles were computed using calculatePvalue() with granularity = 1e-4. We further filtered the result to only include motifs of transcription factor genes expressed in each tested tissue and focused on variants overlapping regulatory regions. For each variant, we note the affected TF motif and the associated target eGene to generate candidate TF–eGene regulatory links.

### Mendelian randomization analyses between *cis*-eQTL and knee-related osteoarthritis

To evaluate putative causal effects of gene expression levels of colocalizing genes on knee-related osteoarthritis, we conducted Mendelian randomization analyses^24^. As exposures, we used the generated *cis*-eQTL data from the colocalizing genes derived from osteoarthritis primary tissues (low-grade and high-grade cartilage, synovium, and fat pad), and as the outcome, we employed GWAS summary statistics from knee osteoarthritis, osteoarthritis at any site, and total knee replacement^6^. Instrumental variables for the analyses were identified for each gene and tissue combination from the exposure summary statistics as independent variants significantly associated with gene expression. We defined independence through clumping of all significantly associated variants based on LD using the European reference LD panel from the 1000 Genomes project^65^ within a 10 Mb window on either side of the index variants, and with a strict threshold of *R*^2^ = 0.001. In cases where an instrumental variable was not present in the outcome data, we conducted an LD-based proxy search using *R*^2^ > 0.7 in the same LD reference panel as in the clumping step. For the proxy search, we used the *LDlinkR* R package (version 1.3.0). We used the *TwoSampleMR* R package (version 0.5.7) curated by MR-Base^87^ to run the causal inference analysis based on the inverse variance weighted method (IVW) and the Wald ratio if only one instrumental variable was available.

Mendelian randomization relies on certain assumptions, which can partially be validated. Firstly, we ensured that the selected instrumental variables were strongly associated with the exposure by filtering out variants with an F-statistic <10. The F-statistic was calculated as the ratio between the squared effect size estimates and its squared standard error (beta^2^/se^2^)^24^. In cases where multiple instrumental variables were available, we reanalyzed using the inverse variance weighted method with Steiger-filtered exposure data to assess reverse causation by comparing the direction of causal effect estimates. Additionally, if more than two instrumental variables were available, weighted median and MR-Egger analyses were conducted to assess the direction of effect concordance among different estimates of causal effects. To indicate a lack of evidence of pleiotropy, we required the MR-Egger intercept to be equal to zero (FDR-adjusted *p*-value > 0.05). To assess heterogeneity, we examined the FDR-adjusted *p*-value of the Cochran’s Q-statistic, requiring it to be larger than 0.05 for no evidence of heterogeneity. Finally, we have adjusted the *p*-values of the causal estimates for multiple testing burdens using FDR correction. Hence, evidence for a causal effect was defined based on the following criteria:

1. FDR-adjusted *p*-value of the Wald ratio or IVW method <0.05
2. If applicable, concordant direction of effect across different tested methods
3. Lack of evidence of pleiotropy (FDR-adjusted MR-Egger intercept *p*-value > 0.05)
4. Lack of evidence of heterogeneity (FDR-adjusted Q-statistic *p*-value > 0.05)

### Pathway enrichment analysis

We have conducted over-representation analysis for different gene sets using gprofiler2 (v0.2.4)^88^ using all the genes measured in our study as background. For the genes showing evidence of colocalization with knee-related osteoarthritis phenotypes, we used all genes that are eQTL as the background. We queried KEGG^89^, Reactome^90^, WikiPathways^91^, and Gene Ontology^92^ Biological Process terms. We required a minimum overlap of two genes to report enrichment. The significance threshold was set at false discovery rate (FDR) < 0.05.

### Lookup of prioritized colocalizing genes

To gain further insights into the biology and function of the prioritized genes, we searched these genes in knockout mice and rare and syndromic human diseases databases. Firstly, to look at rare and syndromic diseases linked to the prioritized genes, we extracted data from the Online Mendelian Inheritance in Man (OMIM^27^) (https://omim.org/) via its API. We defined association with musculoskeletal phenotypes if the disease showed any skeletal or muscle soft tissue phenotype. Secondly, we used the batch query functionality of the Mouse Genome Informatics^26^ database (http://www.informatics.jax.org/) to retrieve knockout mice phenotypes for all prioritized genes. We then screened the output for the following musculoskeletal phenotypes: bone, muscle, skeleton, osteo, arthritis, muscular, joint, body size, growth, skeletal, stature, height, limb, appendage, cartilage, chondrocyte, body length, brachypodia, brachydactyly, brachyphalangia, femur, tibia, ulna, fibula, humerus, radial, spine curvature, posture, vertebral arch, and syndactyly.

### Drug targets analysis

We have queried the druggability of the colocalizing genes using the OpenTargets^21^ database (version 23.09), which includes information about 61,264 unique drug targets. Open Targets defines drugs as any bioactive molecule with drug-like properties, based on the EMBL-EBI ChEMBL database^93^. This definition excludes vaccines, blood products, cell therapies, and multi-ingredient drugs. We considered drugs with information on the indication that had completed at least a Phase 2 trial.

### Spatial transcriptomics study participants

The spatial transcriptomics project has been evaluated and approved by the Research Ethics Committee of the University of Tartu (379/M-10, 12th of June 2023). All study participants have voluntarily signed the informed consent form of the study. For spatial transcriptomics analyses, three knee osteoarthritis patients from the Orthopaedics Clinic at the Tartu University Hospital were recruited. They were all female with average age 65 (standard deviation 6.78) years and with European ethnicity. They all have been diagnosed with knee osteoarthritis with Kellgren and Lawrence grade 4 (severe) and were hospitalized for the joint replacement surgery due to osteoarthritis. They did not have post-traumatic osteoarthritis, treatment with bisphosphonates, dysplasia, inflammatory arthritis (such as ankylosing spondylitis, rheumatoid arthritis, psoriatic arthritis), gout, or lupus.

### Sample collection and preparation

Punch biopsies were taken from the tibial plateau (surgical waste from arthroplasty surgery) immediately after removal from the joint. Biopsies were collected from macroscopically degraded regions. All biopsies contained cartilage and subchondral bone tissue layers. Tissues were transferred into 4% formaldehyde solution at room temperature (RT). Within 2 h, the tissues were cut into smaller pieces (smallest dimension ~2 mm) and incubated in fresh 4% formaldehyde solution at RT for at least 72 h with mild agitation. After this, tissues were washed with nuclease-free water for 15 min at RT with mild agitation three times and then transferred into 14% EDTA solution with pH at least 8 for the decalcification. Tissues were incubated in EDTA solution at RT with mild agitation and solution changed daily for altogether 11 days. After that, the tissues were washed with nuclease-free water for 15 min at RT with mild agitation three times and delivered to Tartu University Hospital Pathology Service laboratory for FFPE block preparation. The FFPE blocks were stored at 4 °C until further use. For conducting the spatial transcriptomics sequencing, the tissues were sectioned (5 µm) and placed onto Leica Bond plus slides (Leica Biosystems GmbH, Germany). In parallel, the sections were graded based on the OARSI grading system and all tissues were at OARSI grade 3.

### Spatial transcriptomics

Spatial transcriptomics analysis was conducted with Nanostring GeoMx Digital Spatial Profiler platform, applying Human Whole Transcriptome Atlas panel (Bruker Spatial Biology Inc, USA) and following sequencing on Illumina platform (Illumina Inc, USA). For selecting the areas of illumination (AOIs), the DNA dye signals were used. The AOIs included in the study were selected from the following tissue areas: (1) cartilage superficial zone, (2) cartilage middle zone, (3) cartilage deep zone, (4) calcified cartilage (below tidemark).

### Evaluating the zone-level specificity of colocalizing eGenes

To evaluate in which osteochondral tissue zones the colocalizing genes are expressed, the following workflow was applied. (1) We looked, which colocalizing genes were detected by Nanostring GeoMX platform in the samples. 45 out of the 136 colocalizing genes were expressed in at least one zone. (2) We considered the genes not expressed if the probe count was below 6 in at least two samples out of three per zone group^94^. The probe counts can be found in Supplementary Data 14.

### Reporting summary

Further information on research design is available in the Nature Portfolio Reporting Summary linked to this article.

## Supplementary information

Supplementary Information

Description of Additional Supplementary Files

Supplementary Data 1

Supplementary Data 2

Supplementary Data 3

Supplementary Data 4

Supplementary Data 5

Supplementary Data 6

Supplementary Data 7

Supplementary Data 8

Supplementary Data 9

Supplementary Data 10

Supplementary Data 11

Supplementary Data 12

Supplementary Data 13

Supplementary Data 14

Supplementary Data 15

Supplementary Data 16

Supplementary Data 17

Reporting Summary

Transparent Peer Review file

## Source data

Source Data

## Data Availability

The knee-related osteoarthritis GWAS summary statistics used for colocalization analysis (knee osteoarthritis, total knee replacement, osteoarthritis at any site) can be obtained from ref. ^6^ (https://doi.org/10.1038/s41586-025-08771-z) and through Musculoskeletal Knowledge Portal^95^ (mskkp.org) (https://msk.hugeamp.org/downloads.html). The summary statistics for the significant *cis*- and *trans*-eQTLs and the results from colocalization are provided in the Supplementary Data. The full eQTL summary statistics are available for download from the Musculoskeletal Knowledge Portal^95^ (https://msk.hugeamp.org/downloads.html). Source Data are provided with this paper. Other publicly available datasets used in this study: 1000 Genomes Project (Phase 3, GRCh38) (https://www.internationalgenome.org/); ENCODE-SCREEN candidate *cis*-regulatory elements (V4) (https://screen.encodeproject.org/); Primary osteoarthritis chondrocyte Hi-C data: Bittner et al.^28^ (https://doi.org/10.1136/ard-2023-224945); Knee cartilage ATAC-seq data: Liu et al.^39^ (https://doi.org/10.1038/s41598-018-33779-z); Skeletal histone-mark ChIP-seq data: Ferguson et al.^86^ (https://doi.org/10.1038/s41467-018-05573-y). Other resources: Ensembl Regulatory Build^82^, Open Targets Platform^21^, OMIM^27^, Mouse Genome Informatics^26^. Source data are provided with this paper.

## Code availability

No custom software or original code was developed for this study; all analyses used publicly available tools and packages, cited in the “Methods” with version numbers. Code for reproducing the main manuscript figures can be found at GitHub (https://github.com/georgia-katsoula/OA-eQTL-figures)^96^. Additional scripts may be found in our group repositories at https://github.com/hmgu-itg.
