# Supplementary material for: Transcriptome analysis in osteoarthritis primary tissues identifies high-confidence effector genes
Source: Nat Commun. 2026 Jun 29;17:8128. doi: 10.1038/s41467-026-74993-y (PMC13458226; doi:10.1038/s41467-026-74993-y)
Supplement: Supplementary file 1 [file 41467_2026_74993_MOESM1_ESM.pdf]

Transcriptome analysis in osteoarthritis primary tissues identifies high-confidence effector genes

Supplementary Information

Supplementary Figures

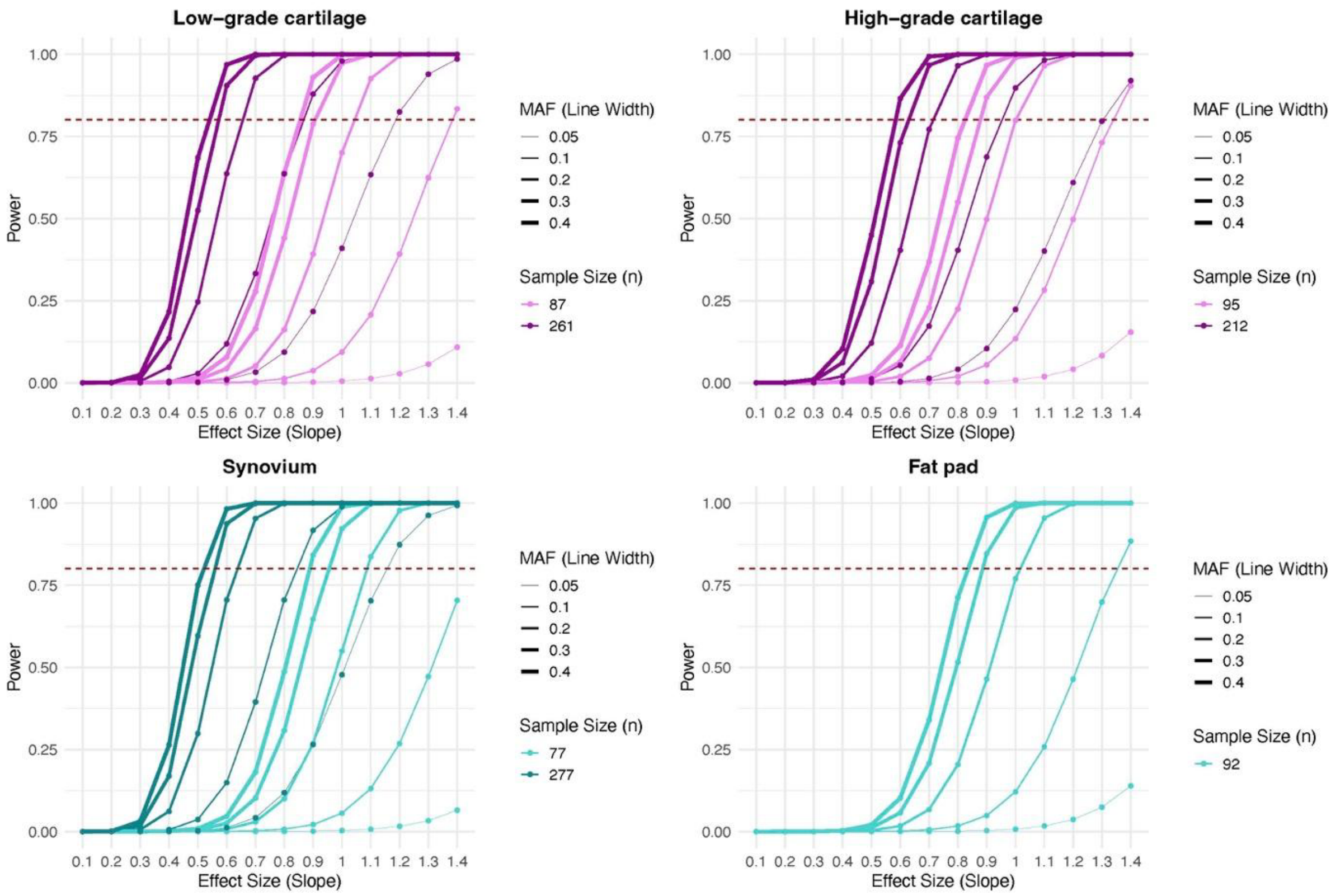

Supplementary Figure 1: Estimated statistical power to detect cis-eQTLs as a function of effect size (slope), minor allele frequency (MAF), and sample size, shown separately for each osteoarthritis primary tissue (low-grade cartilage, high-grade cartilage, synovium, fat pad). Within each panel, colour shade denotes sample size, corresponding to the cohorts of Katsoula & Arruda *et al.* and Steinberg *et al.*[14], and line width denotes MAF (0.05, 0.1, 0.2, 0.3, 0.4). The horizontal dashed line marks the conventional 80% power threshold. Source data are provided as a Source Data file.

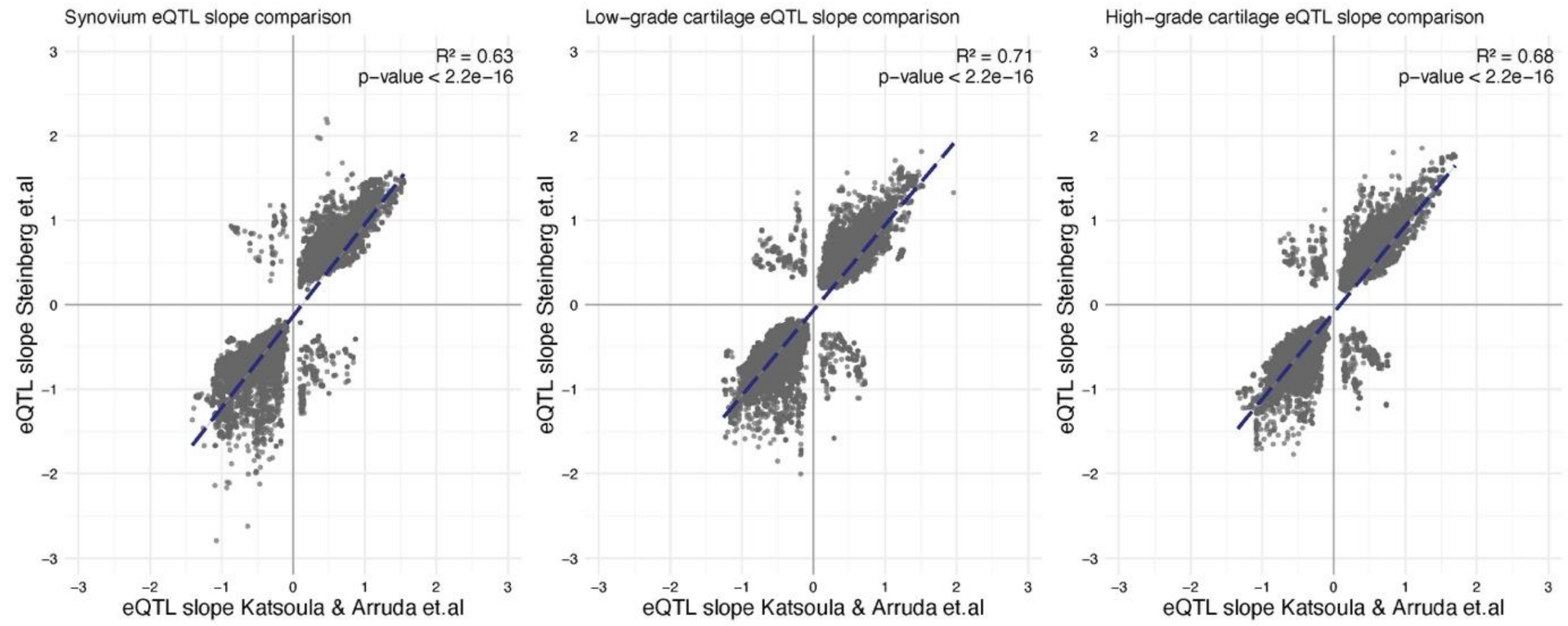

Supplementary Figure 2: Comparison of *cis*-eQTL effect sizes between our study and Steinberg et al.[14]. Scatter plots of allelic effect sizes (slopes) for *cis*-eQTLs detected in common between the two studies, shown separately for synovium, low-grade cartilage, and high-grade cartilage. Each point represents one variant-gene pair; the x-axis shows the effect size estimated in our study (Katsoula & Arruda et al.) and the y-axis the corresponding estimate from Steinberg et al.[14]. The dashed blue line is the linear regression fit, with the coefficient of determination (R^2) and regression p-value indicated in each panel. Source data are provided as a Source Data file.

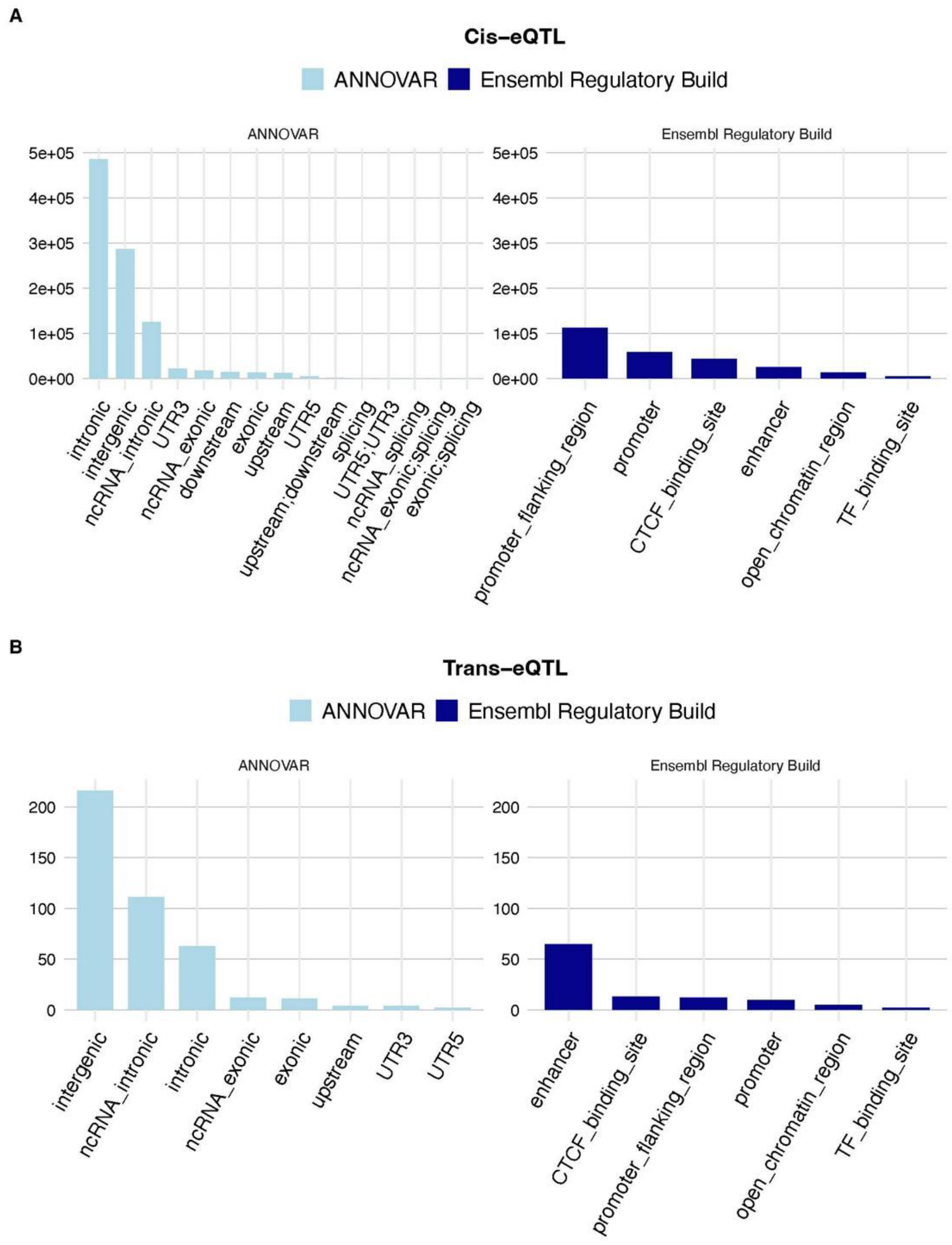

Supplementary Figure 3: Genomic and regulatory annotation of *cis*- and *trans*-eQTLs. Distribution of significant eQTL variants across functional categories. (**A**) *cis*-eQTLs and (**B**) *trans*-eQTLs, pooled across all four osteoarthritis primary tissues. Within each panel, variants are annotated by genomic context using ANNOVAR[82] (light blue) and by overlap with regulatory elements from the Ensembl Regulatory Build[83] (dark blue); bar height is the number of variants in each category. Source data are provided as a Source Data file.

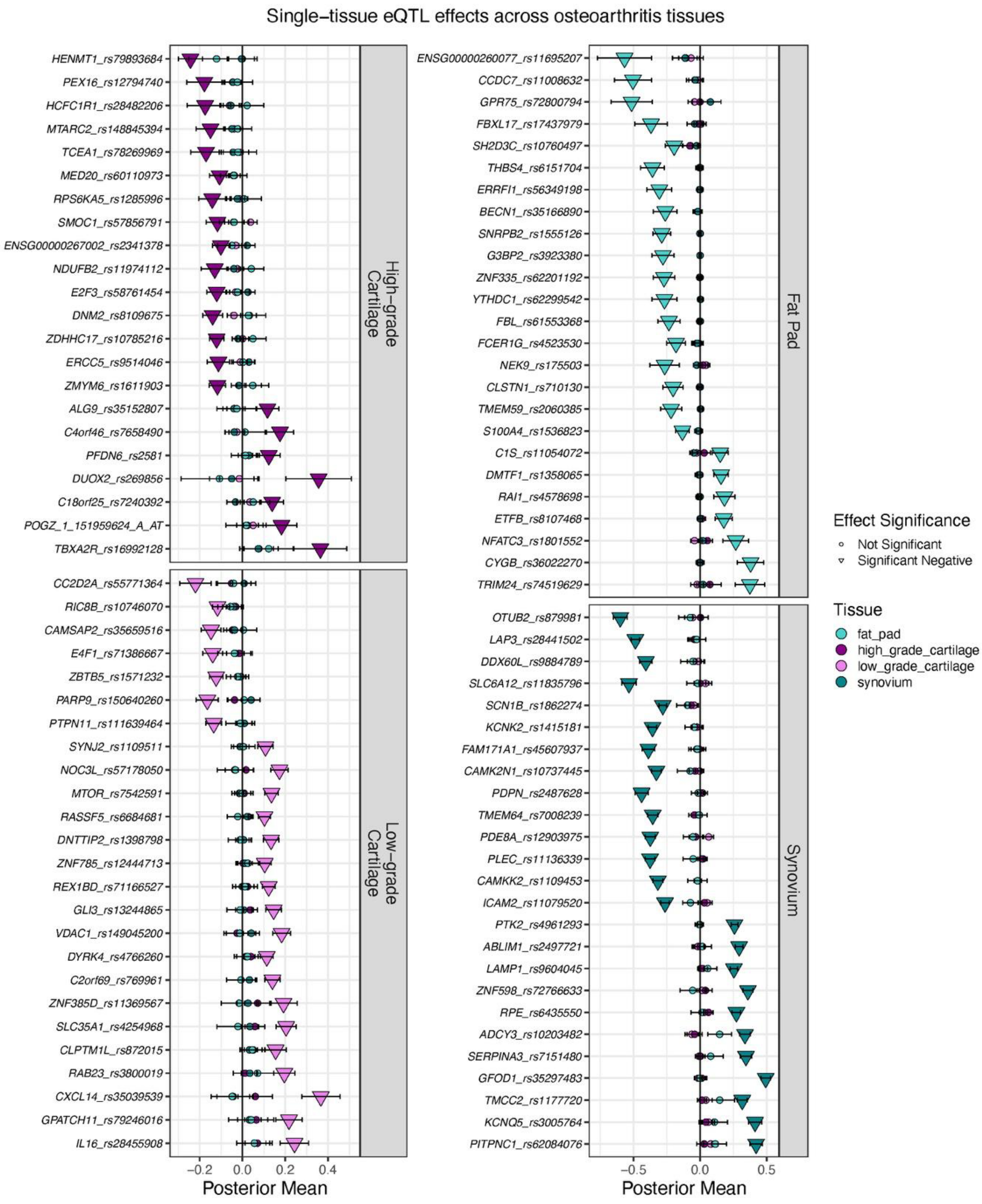

Supplementary Figure 4: eQTL-eGene pairs with effects detected in a single primary osteoarthritis tissue, identified through mashr[18]. Mashr was applied to genes expressed in all four tissues (n=13,181 genes; low-grade cartilage n=261, high-grade cartilage n=212, synovium n=277, fat pad n=92). Pairs were retained if they reached LFSR < 0.05 in one tissue while LFSR ≥ 0.1 in all others, showed an effect size at least two-fold larger in magnitude than in the remaining tissues, and had an absolute posterior mean ≥ 0.1. The top 25 pairs per tissue, ranked by LFSR in the tissue of effect, are shown. The x-axis indicates the posterior mean effect size; error bars represent the posterior standard deviation. Significant associations (LFSR < 0.05) are shown as triangles, non-significant associations as circles. Fill colour indicates the tissue. Source data are provided as a Source Data file.

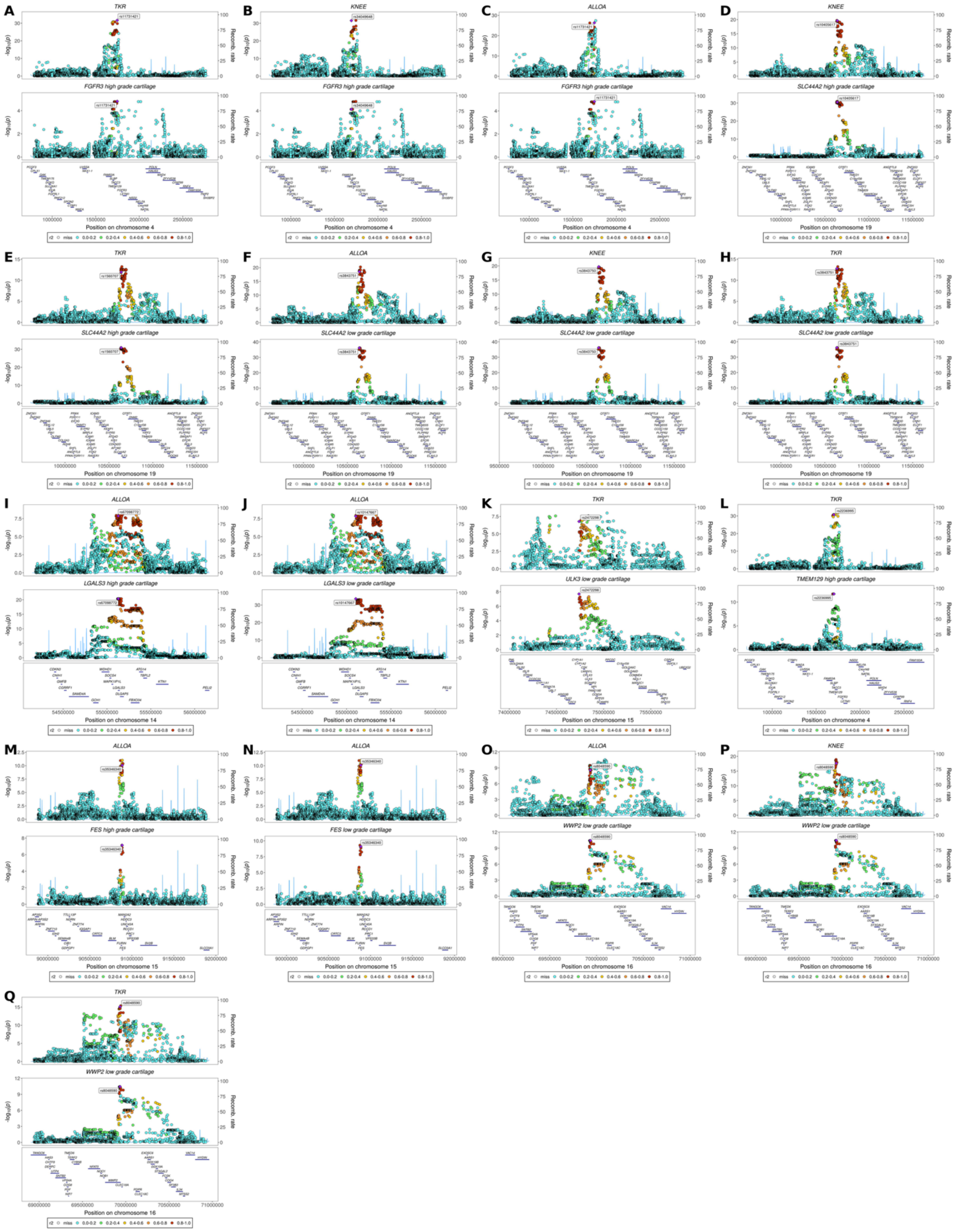

Supplementary Figure 5: Regional association plots at loci where a colocalizing eQTL resides within an enhancer of an active promoter-enhancer loop.

(A–Q) Each panel shows stacked regional association plots for a colocalizing osteoarthritis GWAS-eQTL pair. These loci were selected because the colocalizing variant - included in the

95% credible set for the shared causal signal from colocalization of the eQTL with the osteoarthritis GWAS[6] lies within an enhancer that is engaged in an active promoter-enhancer loop with the target gene, derived from Hi-C data of primary knee osteoarthritis chondrocytes[28]. In each panel the upper plot shows the osteoarthritis GWAS association and the lower plot the corresponding *cis*-eQTL for the gene and tissue indicated. The x-axis corresponds to the genomic position (GRCh38), the left y-axis to the association $-\log_{10}(P)$, and the right y-axis to the recombination rate (cM/Mb; blue line). Variants are coloured by linkage disequilibrium (r^2), with the lead variant, from blue (r^2=0) to red (r^2=1). Gene models are shown beneath each plot pair. Osteoarthritis phenotypes: KNEE = knee osteoarthritis; ALLOA = osteoarthritis at any site; TKR = total knee replacement surgery. Source data are provided as a Source Data file.

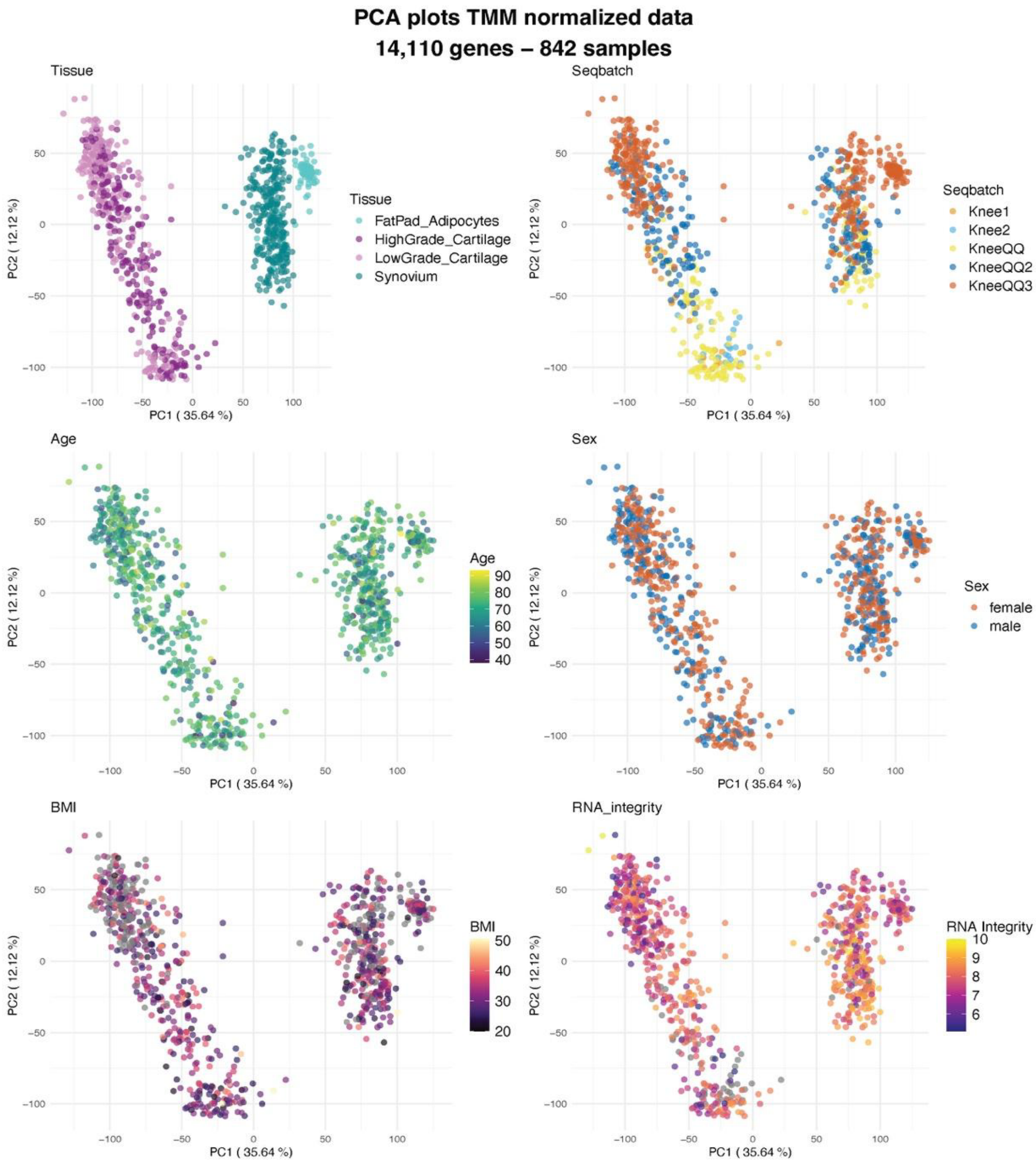

Supplementary Figure 6: Principal component analysis of gene expression across all samples. Principal component analysis (PCA) of gene expression across all samples. PCA was performed on normalised expression of 14,110 genes in 842 samples. All six panels show the same projection of PC1 (35.64% of variance) against PC2 (12.12%), with each point representing one sample coloured by a different covariate: tissue, sequencing batch, age, sex, BMI, and RNA integrity number. Source data are provided as a Source Data file.

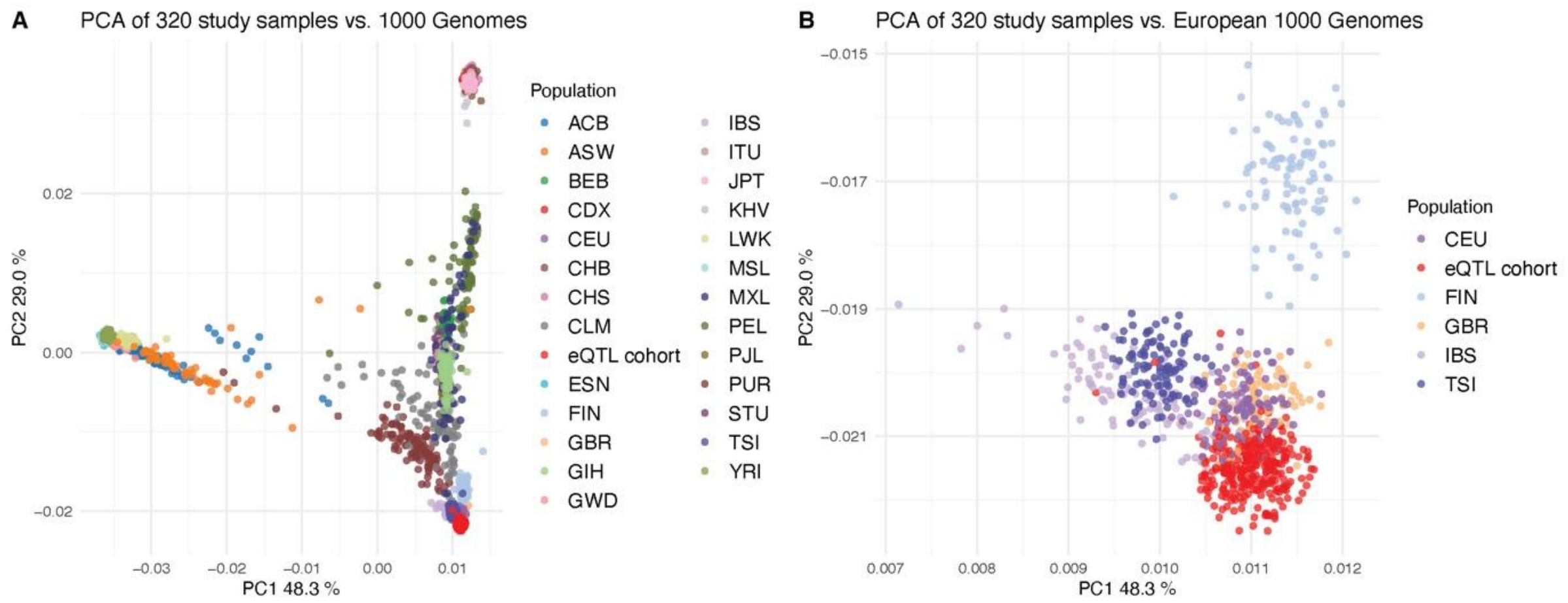

Supplementary Figure 7: Genetic ancestry of the eQTL cohort relative to 1000 Genomes reference populations. Principal component analysis of genotypes from the 320 eQTL cohort samples projected alongside the 1000 Genomes Project Phase 3 reference panel. Each point is an individual, coloured by population; PC1 and PC2 explain 48.3% and 29.0% of variance, respectively. (**A**) All 1000 Genomes populations together with the eQTL cohort (red). The cohort overlaps the European reference samples, consistent with predominantly European ancestry. (**B**) Zoomed view restricted to the European 1000 Genomes populations (CEU, FIN, GBR, IBS, TSI) and the eQTL cohort (red), showing the cohort's position relative to finer-scale European structure.

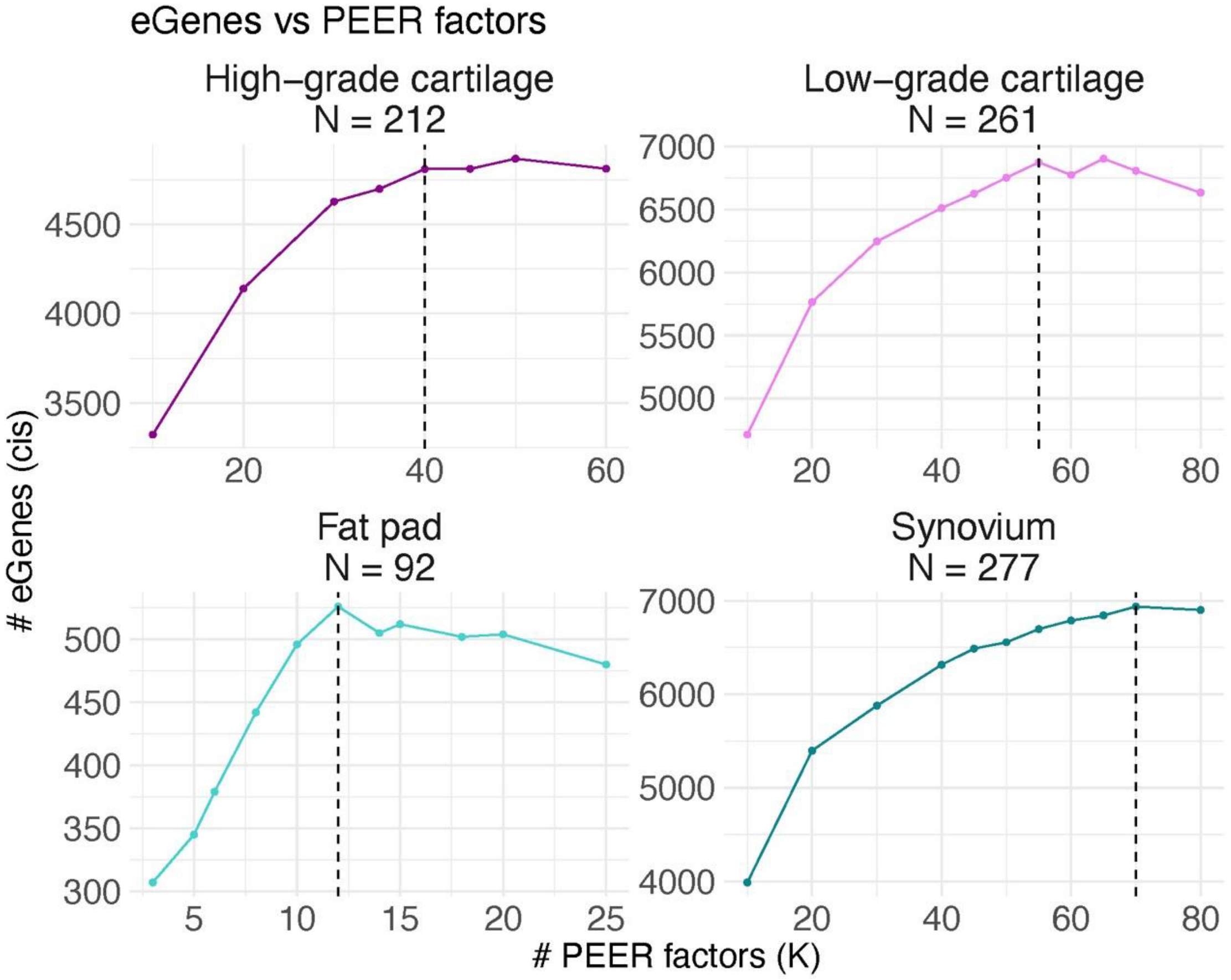

Supplementary Figures 8: Optimization of PEER covariates for cis eQTL discovery. For each tissue, we varied the number of PEER factors, K, included as covariates in the *cis*-eQTL mapping model and recorded the resulting number of significant *cis*-eGenes. Curves show the number of detected *cis*-eGenes as a function of K in high grade cartilage (n=212), low grade cartilage (n=261), fat pad (n=92), and synovium (n=277). Vertical dashed lines indicate the selected K values used in downstream analyses, chosen near the point where eGene discovery reaches a plateau, to maximize discovery while avoiding over correction, high grade cartilage K=40, low grade cartilage K=55, fat pad K =12, synovium K=70. Source data are provided as a Source Data file.
