# Supplementary material for: Transcriptome analysis in osteoarthritis primary tissues identifies high-confidence effector genes
Source: Nat Commun. 2026 Jun 29;17:8128. doi: 10.1038/s41467-026-74993-y (PMC13458226; doi:10.1038/s41467-026-74993-y)
Supplement: Supplementary file 2 [file 41467_2026_74993_MOESM20_ESM.pdf]

nature portfolio

Corresponding author(s): Eleftheria Zeggini

Last updated by author(s): 16.06.2026

Reporting Summary

Nature Portfolio wishes to improve the reproducibility of the work that we publish. This form provides structure for consistency and transparency in reporting. For further information on Nature Portfolio policies, see our Editorial Policies and the Editorial Policy Checklist.

Statistics

For all statistical analyses, confirm that the following items are present in the figure legend, table legend, main text, or Methods section.

n/a	Confirmed	
☐	☒	The exact sample size (*n*) for each experimental group/condition, given as a discrete number and unit of measurement
☐	☒	A statement on whether measurements were taken from distinct samples or whether the same sample was measured repeatedly
☐	☒	The statistical test(s) used AND whether they are one- or two-sided<br>*Only common tests should be described solely by name; describe more complex techniques in the Methods section.*
☐	☒	A description of all covariates tested
☐	☒	A description of any assumptions or corrections, such as tests of normality and adjustment for multiple comparisons
☐	☒	A full description of the statistical parameters including central tendency (e.g. means) or other basic estimates (e.g. regression coefficient) AND variation (e.g. standard deviation) or associated estimates of uncertainty (e.g. confidence intervals)
☐	☒	For null hypothesis testing, the test statistic (e.g. *F*, *t*, *r*) with confidence intervals, effect sizes, degrees of freedom and *P* value noted<br>*Give P values as exact values whenever suitable.*
☐	☒	For Bayesian analysis, information on the choice of priors and Markov chain Monte Carlo settings
☒	☐	For hierarchical and complex designs, identification of the appropriate level for tests and full reporting of outcomes
☐	☒	Estimates of effect sizes (e.g. Cohen's *d*, Pearson's *r*), indicating how they were calculated

Our web collection on statistics for biologists contains articles on many of the points above.

Software and code

Policy information about availability of computer code

Data collection	RNA sequencing data were generated using standard Illumina protocols. RNA was extracted using the Qiagen AllPrep RNA Mini Kit. Poly(A)-selected RNA libraries were prepared using Illumina TruSeq RNA Sample Prep v2 kits for batches 1-4 and the SMART-Seq Ultra Low Input RNA Kit for batch 5, and sequenced on Illumina HiSeq 2000, HiSeq 4000 (75 bp paired-end), and NovaSeq 6000 platforms. Whole-genome sequencing libraries were prepared using standard Illumina paired-end DNA protocols and sequenced on the NovaSeq platform. Spatial transcriptomics data were generated using the NanoString GeoMx Digital Spatial Profiler with the Human Whole Transcriptome Atlas panel, followed by sequencing on an Illumina platform. No custom software was used for data generation.
Data analysis	RNA-seq reads were aligned to the Ensembl GRCh38 reference genome (release 105) using STAR v2.7.9a. Gene-level quantification and quality control were performed using featureCounts (Subread v2.0.3), Picard Tools v2.18.25, RSeQC v4.0.0, the rrcov R package, and RSEM v1.3.3. Whole-genome sequencing data were processed using samtools v1.14, bedtools v2.30.0, the nf-core/sarek pipeline v2.7.1, and GATK v4.2.5.0. Variant quality control and population structure analyses were performed using bcftools v1.21, PLINK v1.9 and v2.0 (alpha), VerifyBamID v1.13, and the Ancestry and Kinship Toolkit (akt) v0.3.3. Variants were annotated using ANNOVAR (v2025-07-29). Power analysis was performed using powerEQTL v0.3.4. The cis- and trans-eQTL analyses were conducted using tensorQTL v1.0.9 and MatrixEQTL v2.3, with hidden confounders inferred using the PEER framework (peer v1.0) and multiple-testing correction using the qvalue package v2.28.0. eQTL effect sizes across tissues were modelled using mashr v0.2.79. Cross-study comparison used the Bioconductor annotation package SNPlocs.Hsapiens.dbSNP144.GRCh37. Genetic colocalization analyses were performed using the coloc R package v5.2.2 (coloc.abf and coloc.susie). Transcription factor binding motif analysis used motifbreakR v2.20. Mendelian randomization analyses were conducted using TwoSampleMR v0.5.7 and LDlinkR v1.3.0. Differential expression filtering used edgeR v3.38.4, pathway enrichment used gprofiler2 v0.2.4, and figures were generated using ggplot2 v3.5.0 All statistical analyses were performed in R v4.1.3 and v4.4.3.

No custom software or original code were developed for this study; all analyses used publicly available tools and packages, cited in the Methods with version numbers. Code for reproducing the main manuscript figures is available at https://github.com/georgia-katsoula/OA-eQTL-figures/ . Additional scripts may be found in our GitHub repositories (https://github.com/hmgu-itg).

For manuscripts utilizing custom algorithms or software that are central to the research but not yet described in published literature, software must be made available to editors and reviewers. We strongly encourage code deposition in a community repository (e.g. GitHub). See the Nature Portfolio guidelines for submitting code & software for further information.

Data

Policy information about availability of data

All manuscripts must include a data availability statement. This statement should provide the following information, where applicable:

- Accession codes, unique identifiers, or web links for publicly available datasets
- A description of any restrictions on data availability
- For clinical datasets or third party data, please ensure that the statement adheres to our policy

The knee-related osteoarthritis GWAS summary statistics used for colocalization analysis (knee osteoarthritis, total knee replacement, osteoarthritis at any site) can be obtained from Hatzikotoulas et al. (https://doi.org/10.1038/s41586-025-08771-z) and through Musculoskeletal Knowledge Portal (mskkp.org) (https://msk.hugeamp.org/downloads.html).

The summary statistics for the significant cis- and trans-eQTLs and the results from colocalization are provided in the Supplementary Data.
The full eQTL summary statistics are available for download from the Musculoskeletal Knowledge Portal (https://msk.hugeamp.org/downloads.html). Source Data are provided with this paper.

Other publicly available datasets used in this study:

1000 Genomes Project (Phase 3, GRCh38) (https://www.internationalgenome.org/);
ENCODE-SCREEN candidate cis-regulatory elements (V4) (https://screen.encodeproject.org/);

Primary osteoarthritis chondrocyte Hi-C data: Bittner et al. (https://doi.org/10.1136/ard-2023-224945);
Knee cartilage ATAC-seq data: Liu et al. (https://doi.org/10.1038/s41598-018-33779-z);
Skeletal histone-mark ChIP-seq data: Ferguson et al. (https://doi.org/10.1038/s41467-018-05573-y).
Other resources: Ensembl Regulatory Build, Open Targets Platform, OMIM, Mouse Genome Informatics.

Research involving human participants, their data, or biological material

Policy information about studies with human participants or human data. See also policy information about sex, gender (identity/presentation), and sexual orientation and race, ethnicity and racism.

Reporting on sex and gender	Self-reported sex information was recorded at recruitment and verified through genetics. All the follow up analyses are based on sex adjusted information. Out of the 320 individuals 176 were females; 55%.
Reporting on race, ethnicity, or other socially relevant groupings	All the study participants included in this study are of European descent as determined by genetics.
Population characteristics	Participants comprised men and women with symptomatic knee osteoarthritis undergoing total knee replacement surgery. After quality control, the final study population consisted of 320 individuals. The mean age was 70.5 years (SD 8.86), with a median age of 72.0 years (range 38-93). The cohort included 176 females (55.0%) and 144 males (45.0%). Mean body mass index (BMI) was 32.0 kg/m^2 (SD 5.83; median 31.6, range 20.0–50.5), with BMI data missing for 22.8% of participants. Mean body weight was 88.0 kg (SD 17.3), and mean height was 1.65 m (SD 0.10). All participants met predefined clinical inclusion criteria and provided matched joint tissue samples for downstream analyses.
Recruitment	Participants were recruited at the point of total knee replacement surgery through the South Yorkshire and North Derbyshire Musculoskeletal Biobank (University of Sheffield). The study focused on recruiting participants of European ancestry, which was subsequently confirmed by genotype-based analysis, and therefore the results may not be generalizable to populations of other ancestral backgrounds. For knee osteoarthritis patients, inclusion criteria required the absence of inflammatory arthropathies and of medications known to affect bone or cartilage metabolism or disease-modifying therapies for inflammatory conditions. As participants were ascertained at surgery, the cohort represents individuals with end-stage disease electing joint replacement, which limits generalisability to earlier disease stages. Participation in the study did not involve any actual or perceived secondary health benefits.
Ethics oversight	This work was approved by Oxford NHS REC C (10/H0606/20 and 15/SC/0132). Samples were collected under Human Tissue Authority license 12182, Sheffield Musculoskeletal Biobank, University of Sheffield, UK; and under National Research Ethics approval reference 11/EE/0011, Cambridge Biomedical Research Centre Human Research Tissue Bank, Cambridge University Hospitals, UK. All patients provided written, informed consent prior to participation in the study.

Note that full information on the approval of the study protocol must also be provided in the manuscript.

Field-specific reporting

Please select the one below that is the best fit for your research. If you are not sure, read the appropriate sections before making your selection.

- [x] Life sciences
- [ ] Behavioural & social sciences
- [ ] Ecological, evolutionary & environmental sciences

For a reference copy of the document with all sections, see nature.com/documents/nr-reporting-summary-flat.pdf

Life sciences study design

All studies must disclose on these points even when the disclosure is negative.

Sample size	Sample size was not predetermined by statistical methods. We included all available osteoarthritis patients with matched whole-genome sequencing and tissue-specific RNA-sequencing data passing quality control (n = 320; low-grade cartilage n = 261, high-grade cartilage n = 212, synovium n = 277, fat pad n = 92). A post hoc power analysis using the powerEQTL R package (powerEQTL.SLR) across a range of minor allele frequencies, effect sizes, and sample sizes (Supplementary Figure 1) indicated sufficient power to detect common variants with moderate-to-large cis-eQTL effects.
Data exclusions	RNA-sequencing samples were excluded based on predefined quality control criteria, including low RNA integrity, poor mapping quality, insufficient read depth, abnormal read composition, or failure of strand-specificity checks, resulting in the removal of 42 low-grade cartilage samples, 35 high-grade cartilage samples, 14 synovium samples, and 13 fat pad samples. Additional exclusions included tissue-specific outliers identified by principal component analysis, pooled samples from bilateral knee replacements, redundant cartilage sample pairs, and lowerquality<br>technical replicates. Whole-genome sequencing samples were excluded due to excessive heterozygosity, sequencing depth outliers, genotype discordance, sex mismatches, relatedness, or ancestry outliers identified by principal component analysis (n = 9 individuals). Samples affected by mislabeling, swaps, redundancy, or cross-sample contamination were also removed. Following quality control, highquality RNA-sequencing and whole-genome sequencing data were available for 320 individuals.
Replication	Reproducibility of the cis-eQTL findings was assessed by replication against two independent, previously published eQTL datasets. First, we compared our cis-eQTL signals with those from an earlier study by our group in a subset of the same cohort (Steinberg et al., 2021): approximately 70% of previously reported signals replicated across tissues (69.4% synovium, 68.5% low-grade cartilage, 68.6% high-grade cartilage), with concordant effect directions and highly significant correlation of effect sizes across all tissues ($R^2 = 0.63–0.71$, $p < 2.2 \times 10^{-16}$). Second, we compared our synovium cis-eQTL map with an independent map from a Han Chinese population (Jiang et al., 2024), which showed a lower eGene overlap (~54%). This lower overlap was expected and is attributable to population-specific genetic effects (differences in allele frequencies and linkage disequilibrium) and methodological differences between studies, rather than a failure of replication. All replication attempts were therefore successful and consistent with expectation.
Randomization	Randomization was not applicable. This is an observational study of genetic variants associated with gene expression levels; participants were not allocated to experimental groups, so randomization does not apply.
Blinding	Blinding was not applicable to this study. This is an observational analysis of associations between germline genetic variants and gene expression in primary tissues, with no experimental group allocation or intervention. Genotypes are fixed and assayed independently of expression, so investigator knowledge could not influence the measured associations, and blinding to group allocation is therefore not relevant.

Reporting for specific materials, systems and methods

We require information from authors about some types of materials, experimental systems and methods used in many studies. Here, indicate whether each material, system or method listed is relevant to your study. If you are not sure if a list item applies to your research, read the appropriate section before selecting a response.

Materials & experimental systems

n/a	Involved in the study	
x		Antibodies
x		Eukaryotic cell lines
x		Palaeontology and archaeology
x		Animals and other organisms
x		Clinical data
x		Dual use research of concern
x		Plants

Methods

n/a	Involved in the study	
x		ChIP-seq
x		Flow cytometry
x		MRI-based neuroimaging

Plants

Seed stocks	*Report on the source of all seed stocks or other plant material used. If applicable, state the seed stock centre and catalogue number. If plant specimens were collected from the field, describe the collection location, date and sampling procedures.*
Novel plant genotypes	*Describe the methods by which all novel plant genotypes were produced. This includes those generated by transgenic approaches, gene editing, chemical/radiation-based mutagenesis and hybridization. For transgenic lines, describe the transformation method, the number of independent lines analyzed and the generation upon which experiments were performed. For gene-edited lines, describe the editor used, the endogenous sequence targeted for editing, the targeting guide RNA sequence (if applicable) and how the editor was applied.*
Authentication	*Describe any authentication procedures for each seed stock used or novel genotype generated. Describe any experiments used to assess the effect of a mutation and, where applicable, how potential secondary effects (e.g. second site T-DNA insertions, mosiacism, off-target gene editing) were examined.*
