# Supplementary material for: Transcriptome analysis in osteoarthritis primary tissues identifies high-confidence effector genes
Source: Nat Commun. 2026 Jun 29;17:8128. doi: 10.1038/s41467-026-74993-y (PMC13458226; doi:10.1038/s41467-026-74993-y)
Supplement: Supplementary file 3 [file 41467_2026_74993_MOESM21_ESM.pdf]

nature portfolio

Peer Review File

Transcriptome analysis in osteoarthritis primary tissues identifies high-confidence effector genes

Corresponding Author: Dr Eleftheria Zeggini

This file contains all reviewer reports in order by version, followed by all author rebuttals in order by version.

Version 0:

Reviewer comments:

Reviewer #1

(Remarks to the Author)
The paper by Katsoula, et al., describes the eQTL analysis of four knee tissues of 321 osteoarthritis patients. This is an expansion from a previous study by this group in 95 patients that included 3 of the 4 same tissues (Steinberg, et al, 2021). From the current expanded data, nearly 10,000 eQTL were defined, of which 117 eGenes were co-localized with osteoarthritis GWAS signals. These analyses potentially offer a significant number of new hypotheses for how genetic variation affecting gene transcription is contributing to osteoarthritis. There are some concerns with this study as currently reported. These concerns have been divided into those related to how the analysis was performed and how the results were interpreted.

Analysis concerns.
1) Reads were aligned using star, which has an option to perform WASP to correct for increased reference allele alignments compared to non-reference alternate allele alignments. With genotypes for each sample, this can and should be performed. Was this option used?
2) Mis-identified samples can be problematic in studies this large, as seen even here were two WGS datasets were excluded due to sex mismatches. To determine whether RNA-seq data matches the corresponding genotype data, a program such as verifyBamID (https://genome.sph.umich.edu/wiki/VerifyBamID) should be used to provide confidence that WGS and RNA-seq data were generated from the same individual.
3) The number of PEER factors to include is dependent on the sample cohort being studied. It cannot be inferred from a different study where the type and number of confounding factors can vary. Thus, it is not correct to simply use the number of factors used by GTEx for a similar sample size for this study. As the GTEx paper indicates, “The number of PEER factors was selected to maximize cis-eGene discovery, and this optimization was performed for three sample size bins: tissues with fewer than 150 samples, tissues with ≥150 and <250 samples, and tissues with ≥250 samples.” This should be done to determine the correct number of PEER factors to include.
4) When the PEER analysis was performed, were the other known covariates included in the QTL model (sex, age, batch) first removed from the data? If not, then these factors were likely also identified as PEER factors and these would be accounted for multiple times when correcting the data for the QTL analysis.
5) Why was a power analysis performed? It was described in the methods and included in the supplement, but it was not described in the results and does not appear to have been used in any of the other analyses. Nor was the relevance of this explained in the methods.
6) When comparing current cis-eQTL with previous studies, were only the lead eQTL variants used for the comparison? Or were eQTL from different studies deemed the same if the lead variants were in high LD? Why not use coloc to determine whether signals were similar in both eQTL studies?
7) When determining “distinct eQTL effects in osteoarthritis tissues”, what test was used to determine that “genes (were) expressed exclusively in a single disease tissue”? Simply lacking an eQTL does not mean a gene is not expressed in a particular tissue, so was there a minimum expression level used to deem a gene expressed in a tissue?
8) Was the transcription factor disruption analysis performed on all identified trans-eQTLs? Or just the lead variant? Was there any additional information suggesting which trans-eQTLs were in regulatory elements and thus more likely to bind a TF? Why was this only done on trans-eQTLs and not cis-eQTLs, where likewise many variants could be disrupting TF binding?
9) In the Variant Annotation of eQTL methods section, it mentions Fig S20 which has “distributions of eQTL annotations among both cis- and trans-eQTLs”. This is not mentioned anywhere else and its purpose is not explained. Nor is it explained

what “evaluation” was done.

Interpretation concerns/Text suggestions.
1) eQTL provide evidence that genetic variation affects the transcriptional regulation of the target gene. They do not “reveal the function of disease associated variants”, but rather provide hypotheses for how GWAS variants may contribute to disease. Simply being colocalized with a GWAS signal does not confirm causality.
2) It’s not clear why the authors are treating their prior study (Steinberg, et al) as a seemingly independent study in the Introduction when the current study is a direct extension of that study with overlapping patient samples.
3) eQTL being found only in a single tissue does suggest that regulation of that gene in that tissue is different from the other tissues, but that is all. It does not provide evidence that the genetic variation shown to affect this regulation is related to disease, unless there is more direct evidence for this, i.e. through GWAS. It is not clear why, then, unique eQTL in tissues for genes previously associated with osteoarthritis are that significant other than it supports that the gene is differentially regulated in that tissue. Do you also see significant differential expression? If yes, then this differential regulation could point to potential regulatory changes that lead to this differential expression. Similarly, identifying common eQTL across tissues simply shows that the regulatory mechanism affected by genetic variation is common across tissues. It likewise does not support a role for that genetic variation in disease, so the point of this analysis needs to be clarified.
4) Related to the above, the idea of a “disease progression eQTL” is not well-defined. Again, eQTL found in one of low-grade vs high-grade samples simply suggests that the regulation of that gene changes. If the gene is also differentially expressed between the two, then this points to potential regulatory changes driving this change in expression. The fact that this regulation is affected by genetic variation does not, in itself, suggest that the genetic variation is related to disease progression. The significance of these findings need to be better explained if there is something I am missing.
5) Hi-C data showing links between enhancers that contain an eQTL and the promoter of a target gene provide support for specific variant in the enhancer being the casual variant of the eQTL effect. It also gives a hypothesis for the mechanism of the variant. Why do you assume that this “alters enhancer-promoter interactions”? This may be true, but more specifically, is there evidence for altering transcription factor binding in the enhancer? This would provide evidence for the variant altering TF binding which then changes how the enhancer regulates that target gene. In theory, this could lead to altered enhancer/promoter interactions, but that would be better seen in Hi-C data. Similarly, why not also look for TF disruption for the variants in promoters (related to the question of why TF disruption only done on trans-eQTLs)?
6) In the initial GWAS colocalization discussion, it would be helpful to indicate the different knee phenotypes that were studied with GWAS and make up the 211 loci. This would help when in the prioritization section, these specific phenotypes are mentioned.
7) The spatial transcriptomics data is only for cartilage. What is the significance of a prioritized effector gene that was not colocalized in cartilage being expressed in a cartilage layer (i.e. for HLA-DPB1, KCNN3, LTBP1, etc)? What is the significance of these being found in the superficial layer? More generally, is there other evidence pointing to the increased importance of this layer in arthritis that would support the importance of most of these genes being expressed in that layer?
8) Since most of the prioritized genes were eQTL in cartilage, is it really surprising that pathway enrichment points to chondrocyte related pathways? This seems more like an analysis confirming the function of the prioritized genes than something novel.
9) Druggability search – while it is interesting that some of the genes identified already have drugs that target them specifically for use in arthritis (confirmation of role of that gene in disease), the more interesting are the ones whose drugs were not developed for arthritis. I would have thought more of this section would be focused on discussing these and their potential. What are the drugs currently being developed for? Is the action of the drug in the right direction (i.e inhibitory/excititory) given the direction of effect of the eQTL compared to the GWAS association? Is the drug suitable for long-term use, given this is a chronic condition? I feel a lot more should be explored here.
10) MUSTN1 was highlighted in the abstract, but it’s not clear why. While there was a paragraph devoted to it in the Prioritized Genes results section, the discussion of this gene was similar to many other genes highlighted in the paper. It was not mentioned in the Introduction nor was it a further focus within the Discussion section. Thus why it was chosen for special recognition in the abstract should be made more clear.

Reviewer #2

(Remarks to the Author)
This manuscript interesting and very relevant by Katsoula and colleagues, addresses a significant gap for osteoarthritis genomics research, the identification of OA genes and pathways. Specifically, the strengths of this manuscript are in the use large data sets for eQTL analysis and the investigation of multiple OA relevant tissues, this is cartilage (high- grade, and low-grade), synovium and fat pad. Although, the data itself is highly relevant for the OA field, the manuscript itself lacks depth for the data itself to truly contribute to the field of OA genomics and possible future drug development.

There are three OA relevant tissues examined for eQTLs (High grade/low grade Cartilage, synovium and fat pad), as OA is a disease of the whole joint. Some of these eQTLs are deemed to be tissue specific. How truly tissue specific are these eQTLs? Or how OA specific are they truly? Considering that only 3/4 tissues were examined, with also differences in detection power. Fat pad has a vastly lower sample size then the other examined tissues. It is of course impossible to examine all possible tissues (time/data availability/scope), but there are some already existing eQTL data sets which the authors could use to bring more validity and robustness to their claims of tissue specificity; There have been a few publications from the GeFos consortium (PMID: 37579195, PMID: 36416764) where bone (osteoclast) eQTL’s have been investigated, also in the context of OA specific eQTLs. Or, using the GTEX database to validate that the found “tissue specific”QTLs are indeed tissue specific to the OA tissues.

For each tissue examined one or two “tissue specific” eQTLs are descrined int eh results with their relation to OA

biology/pathology based from literature. The selection of the gene to described seem very arbitrary, also as their functions in OA pathology are then somewhat extrapolated to the full set of eQTL's identified in those tissues. However, multiple tissue eQTL's have been were found (597 unique eGenes in cartilage, 70 in synovium, and 14 in fat pad). Could the authors not perform a formal pathway analysis to describe the identified genes and link them to OA biology in a more structured way? This is also present in the section on differences between high grade and low grade cartilage.

The strengths and important elements of eQTL research for GWAS effector gene identification, is not only the identification of a possible effector gene, but equally so the direction of effect. What is the effect of the OA risk allele on which gene, and the direction part is under developed in this manuscript, a part from the very interesting section on discordant eQTLs. For drug development purposes (or experimental validation) it is vital to understand if the OA risk allele cause for an increase or decrease of the gene expression (i.e. does a OA drug need to increase of decrease the gene expression or pathway). Within the tables the effect/modelled allele is either not stated or not clearly stated – Examples table 2 has the Allele Fequency (AF) but does not state the alleles this AF belongs to or if it represents the effect allele frequency. Table 2: does report the alternate (minor) allele frequency and the allele. However, it is unclear if this is then also the effect or modelled allele. Also the figures do not clearly report or indicate what the effect allele is for the eQTL variant. (figure 3, figure 4)

In addition, to the direction of effect of the OA risk alleles, this new eQTL information can also be used in the pathway analysis, i.e. are the found pathways up or down regulated for OA risk? It would be very valuable to the field to not just have another pathway analysis with more genes, but to have one which also takes into account whether or not according to the GWAS findings the enriched pathways/cellular processes are up or down regulated.

Multiple trans eQTLs were identified, and the authors report that most of the trans-eGenes were also cis-regulated. However, it would be more interesting to know which trans eQTL variant also is a cis eQTL variant. This as most trans eQTLs are throught to represent downstream targets/pathways. Example: snp1 regulates cis-gene Y, snp1 also has trans-eQTL gene z. Suggesting gene y may regulate gene z (or gene z might be downstream of gene Y in the biological pathway). This could greatly strengthen and inform the pathway analysis or even indicate "novel" pathways or doenstream targets for OA genes.

The limitations section in the discussion misses several important limitations of this study and it's findings; notably the strong difference in samples size, i.e., statistical power between the tissues investigated particularly for fat pad. The possibility tissue specific eQTLs may not be present in other not investigated tissues and that Transcriptomics is highly dynamic and that always eQTLs may be missed. (minor: an acknowledgement that all the identified effector genes, would still need (wet-lab) validation).

Minor comments:
Figure 3 and figure 4 seem to depict very similar results, as the high grade and low grade cartilage are both also depicted in figure 3. Could these two figures not be combined into one? Or at least an A and B panel?

Identification of trans eQTLs – The reporting of the numbers of trans eQTLs is confusing and somewhat misleading, example: "363 transeQTLs (12 indepdendent signals)". As similar with GWAS signals many genetic variants are in high LD, leading to a high absolute number of genetic variants in association but due to the LD they just represent one GWAS signal. Thus here the more appropriate reporting is we identified 12 independent trans eQTLs. This si also how earlier in the text the cis eQTLs are presented. And it is actually already quite amazing that with this sample size 12 tras eQTLs were found, as the robust identification of them needs a huge sample size. So no need to make the number sound bigger.

Figure 7 – would be more informative if also the corresponding OA GWAS lead variant is/OA signal is added, Next, to what the variant is to which the direction symbol is referring to in the coloc columns.

Version 1:

Reviewer comments:

Reviewer #1

(Remarks to the Author)
The authors have done well to address my previous concerns. I do not have any additional concerns to raise. This study provides a robust analysis of eQTL in three primary tissues, one at two distinct stages, relevant for osteoarthritis. I believe it significantly adds to the interpretation of the effects of variants with GWAS loci for OA.

Reviewer #3

(Remarks to the Author)
The authors have generally addressed the major issues raised in the original review. I still have the remaining minor concerns that the authors should address:
1. The potential mechanisms of underlying trans-eQTLs (e.g, using motifBreakR) are not convincing; disruption of a transition factor binding site does not explain the mechanism of action of trans-eQTLs. If anything, such an analysis should be added for the cis-eQTLs.
2. It is unfortunate that the authors were not able to overlap their results with tissue-specific data from GTEx or GeFOS leaving the issue of tissue-specific eQTLs unresolved. If there truly is no such possible analysis, the authors should make

sure to strike claims of specificity and note the shortcoming in the discussion.

Open Access This Peer Review File is licensed under a Creative Commons Attribution 4.0 International License, which permits use, sharing, adaptation, distribution and reproduction in any medium or format, as long as you give appropriate credit to the original author(s) and the source, provide a link to the Creative Commons license, and indicate if changes were made.
In cases where reviewers are anonymous, credit should be given to 'Anonymous Referee' and the source.
The images or other third party material in this Peer Review File are included in the article's Creative Commons license, unless indicated otherwise in a credit line to the material. If material is not included in the article's Creative Commons license and your intended use is not permitted by statutory regulation or exceeds the permitted use, you will need to obtain permission directly from the copyright holder.
To view a copy of this license, visit https://creativecommons.org/licenses/by/4.0/

Reviewer’s Comments:

Reviewer #1 (Remarks to the Author)

The paper by Katsoula, et al., describes the eQTL analysis of four knee tissues of 321 osteoarthritis patients. This is an expansion from a previous study by this group in 95 patients that included 3 of the 4 same tissues (Steinberg, et al, 2021). From the current expanded data, nearly 10,000 eQTL were defined, of which 117 eGenes were co-localized with osteoarthritis GWAS signals. These analyses potentially offer a significant number of new hypotheses for how genetic variation affecting gene transcription is contributing to osteoarthritis. There are some concerns with this study as currently reported. These concerns have been divided into those related to how the analysis was performed and how the results were interpreted.
We thank the reviewer for taking the time to review our work and for their comments, which have substantially improved the paper.

Analysis concerns.
1) Reads were aligned using star, which has an option to perform WASP to correct for increased reference allele alignments compared to non-reference alternate allele alignments. With genotypes for each sample, this can and should be performed. Was this option used?
We appreciate the reviewer's suggestion. In our study, RNA-seq reads were aligned with STAR using a standard two-pass alignment strategy (e.g., --twopassMode Basic, --sjdbOverhang 74, --quantMode TranscriptomeSAM GeneCounts). We did not enable --waspOutputMode, as WASP correction is primarily relevant for allele-specific expression (ASE) and splicing QTL analyses (van de Geijn et al., Nat Methods 2015), and the recent STAR+WASP implementation (Asiimwe & Dobin, bioRxiv 2024) is benchmarked with focus on ASE rather than conventional eQTL mapping. Although we acknowledge that WASP could potentially also help in standard eQTL studies, it is primarily used for allele-specific expression (ASE) and splicing QTL analyses. This distinction is also reflected in the GTEx v8 pipeline (GTEx Consortium, Science 2020), where STAR with WASP correction was used solely for ASE and sQTLs, while standard STAR alignment was used for eQTL mapping. Consistent with this, several recent large-scale eQTL studies also do not apply WASP (Kerimov et al., Nat Genet 2021; Panousis et al., Nat Comm 2025; Zhang et al., Nat Comm 2025; Kramer et al., Cell Genom 2024).

2) Mis-identified samples can be problematic in studies this large, as seen even here were two WGS datasets were excluded due to sex mismatches. To determine whether RNA-seq data matches the corresponding genotype data, a program such as verifyBamID (https://genome.sph.umich.edu/wiki/VerifyBamID) should be used to provide confidence that WGS and RNA-seq data were generated from the same individual.
We thank the reviewer for highlighting this point. In the updated version of the manuscript, we have now used verifyBamID (v.1.13), as suggested, to systematically confirm concordance between RNA-seq and WGS data. This analysis identified a small number of discrepancies: two mislabeled samples, one perfect swap between two donors’ synovium samples and three

redundant samples (excluded, as the same donors were already represented with correctly assigned tissues). In addition, we have now further evaluated contamination using the same tool and excluded six samples with contamination estimates above 0.2, consistent with thresholds used in prior studies (Kramer et al., Cell Genomics 2024).

In total, this has led to nine exclusions, two mislabeling corrections, and one sample swap correction. We have now re-ran all analyses after these adjustments, ensuring that only high-quality, correctly matched samples were included in the final dataset. All additional analyses' descriptions have been added to the manuscript's methods section.

See updated Methods paragraph: Preprocessing and QC of Whole Genome Sequencing (WGS) data. Lines: 673-683

Addition to the Methods section:
"We further evaluated the concordance between genotypes and RNA-seq using VerifyBamID (v1.13). We assessed sample mislabeling, swaps, and cross-sample contamination. For each RNA-seq BAM, VerifyBamID was run against the joint WGS genotype VCF to infer the most likely matching donor (BEST) and to estimate mixture/contamination metrics (FREEMIX and CHIPMIX;). Expected donor identities were specified a priori via a sample-to-donor mapping file, and concordance was evaluated by comparing BEST to the expected donor. Based on this, we corrected two mislabeled samples and one complete swap between two donors' synovium samples. We also excluded three redundant samples where the same donors were already represented by correctly assigned tissues. To control for contamination, samples with FREEMIX and/or CHIPMIX > 0.2 after swap/mislabel correction were excluded (six samples), consistent with thresholds used in prior studies[66]."

3) The number of PEER factors to include is dependent on the sample cohort being studied. It cannot be inferred from a different study where the type and number of confounding factors can vary. Thus, it is not correct to simply use the number of factors used by GTEx for a similar sample size for this study. As the GTEx paper indicates, "The number of PEER factors was selected to maximize cis-eGene discovery, and this optimization was performed for three sample size bins: tissues with fewer than 150 samples, tissues with ≥150 and <250 samples, and tissues with ≥250 samples." This should be done to determine the correct number of PEER factors to include.
We thank the reviewer for this comment. In the initial submission, we had selected the number of PEER factors based on the GTEx recommendations for sample size bins. Following the reviewer's suggestion, we have now performed a data-driven optimization within our own cohort. Specifically, for each tissue we have now ran *cis*-eQTL mapping across a grid of PEER factors and examined the number of *cis*-eGenes discovered. As shown in the new figure (see below), the number of eGenes increases with additional PEER factors and then plateaus or decreases, consistent with overfitting at higher values.
Based on these curves, we have now selected the optimal number of PEER factors separately for each tissue (indicated by the vertical dashed lines) and have now updated all downstream analyses accordingly. This ensures that the choice of covariates is tailored to the characteristics of our dataset.

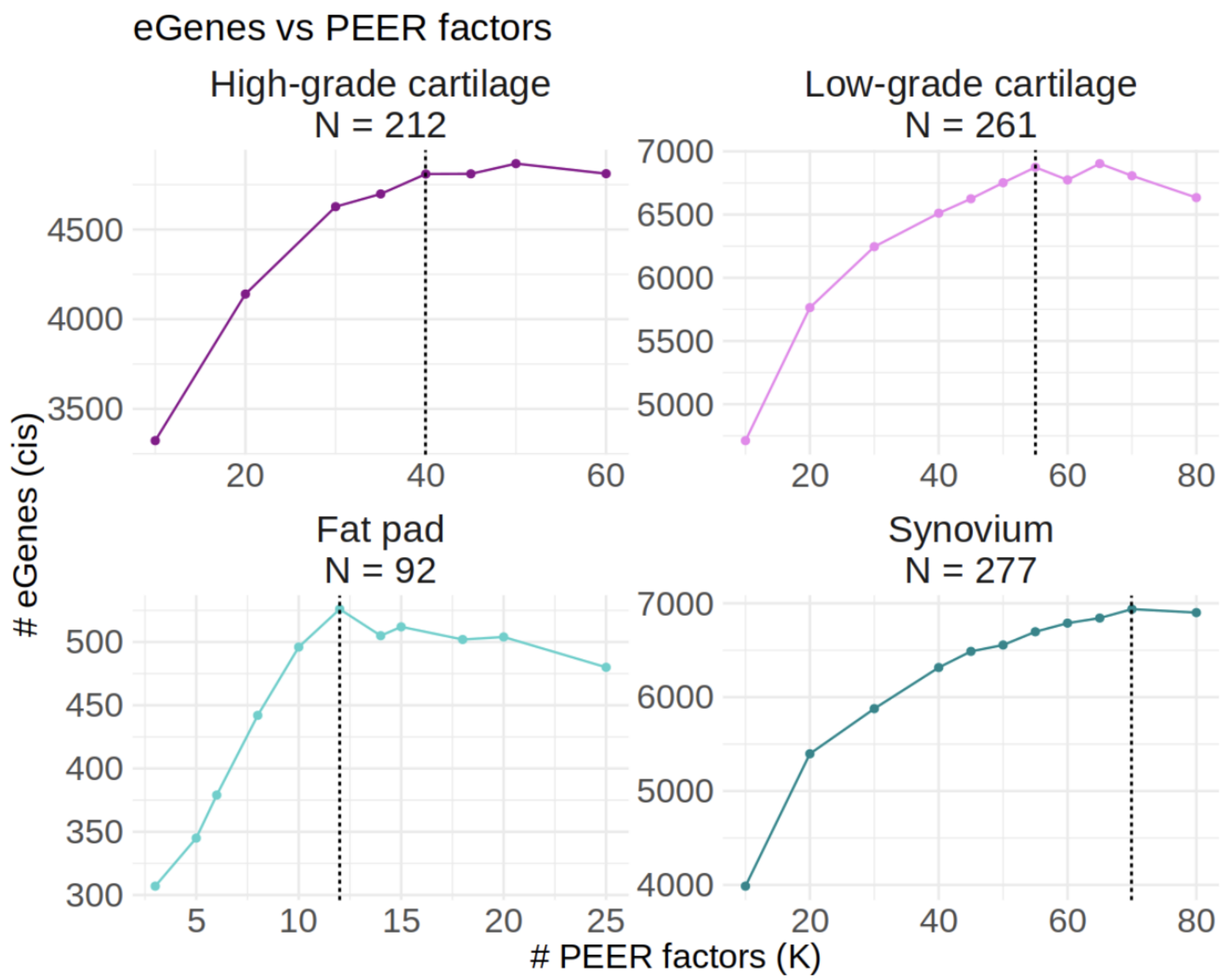

See updated Methods paragraph: Mapping cis-eQTL. Lines: 734-744

Addition to the Methods section:

"To maximize cis-eGene discovery, we optimized the number of PEER factors separately for each tissue while accounting for known covariates (sex, age, and RNA-seq sequencing batch) by providing them to the PEER model via PEER_setCovariates (Stegle et al., Nature Protocols, 2012). For each tissue, we performed cis-eQTL mapping across a grid of PEER factor counts and quantified the number of significant cis-eGenes detected at each setting. We selected the tissue-specific optimum as the first value at which eGene discovery plateaued or began to decline in agreement with GTEx[73] recommendations (Figure S8). The selected number of PEER factors included per tissue in the eQTL model is as follows: low-grade cartilage: 50, high-grade cartilage: 40, synovium: 70, fat pad: 12) We further verified that the inferred latent PEER factors were not strongly associated with the known covariates (ANOVA for categorical covariates and linear regression for continuous covariates)."

4) When the PEER analysis was performed, were the other known covariates included in the QTL model (sex, age, batch) first removed from the data? If not, then these factors were likely also identified as PEER factors and these would be accounted for multiple times when correcting the data for the QTL analysis.

We thank the reviewer for raising this point. In our initial analysis, we had not included the known covariates (sex, age, RNA-seq sequencing batch) when fitting the PEER model. Following the reviewer's suggestion, we have now re-run PEER analysis while explicitly providing these

covariates as known factors. This ensures that they are accounted for only once in the eQTL model and prevents them from being redundantly captured as latent PEER factors. Specifically, we have now passed the known covariates to the PEER model using the *PEER_setCovariates* function (as described in Stegle et al., Nature Protocols 2012, "*Using probabilistic estimation of expression residuals (PEER) to obtain increased power and interpretability of gene expression analyses*"), and have retained only the latent factors. After selecting the optimal number of PEER factors for each tissue, we have now further verified that the resulting latent factors are not strongly correlated with the known covariates, confirming that these effects are handled appropriately. All downstream eQTL analyses have now been repeated with this updated approach.

See updated Methods paragraph: Mapping cis-eQTL.
Lines: 734-744
See updated manuscript paragraph: Osteoarthritis primary tissues cis-eQTL discovery.
Lines: 107-120

5) Why was a power analysis performed? It was described in the methods and included in the supplement, but it was not described in the results and does not appear to have been used in any of the other analyses. Nor was the relevance of this explained in the methods.
The power analysis has been performed to quantify the expected power to detect eQTLs given our available sample sizes, and to compare these expectations with those reported in previous studies. The power analysis provides useful context for interpreting our results and for assessing whether additional samples would likely be needed to increase power in the future. We have now clarified this rationale in the Methods and briefly noted the main conclusions in the Results. The power analysis was not intended for downstream inference, but rather to place our study design and findings in context.

See Methods paragraph: Power analysis (lines: 714-718)
See updated Results paragraph: Osteoarthritis primary tissues cis-eQTL discovery (lines: 108-114)
Addition to Results section:
"We performed cis-eQTL analyses in 320 knee osteoarthritis patients, representing a > 3-fold increase in sample size compared to our previous study[14] by (Figure 1, Figure S1, Table S1). Consistent with this increase, power analyses indicated increased sensitivity to detect common variants with moderate-to-large *cis*-eQTL effects relative to earlier sample sizes (Figure S1)."

Addition to the Methods section:
"To contextualize the eQTL results and to inform future study design, we performed a power analysis to estimate the expected power to detect *cis*-eQTLs given the available sample sizes."

6) When comparing current cis-eQTL with previous studies, were only the lead eQTL variants used for the comparison? Or were eQTL from different studies deemed the same if the lead variants were in high LD? Why not use coloc to determine whether signals were similar in both eQTL studies?

We thank the reviewer for highlighting the need to better explain our methodology. For the comparison with previous *cis*-eQTL studies, we did not restrict only to lead variants but considered all significant variant-gene pairs available from each study. This approach is commonly used in the field for cross-study comparisons of eQTLs (e.g. GTEx Consortium 2020; Võsa et al. 2018), as it provides a straightforward and inclusive way to assess replication across resources.This approach allows for the inclusion of a larger number of variant-gene pairs providing a more conservative assessment of novelty. We have now updated the manuscript to reflect this.

See updated Methods paragraph: Comparison with existing cis-eQTL maps (lines: 793-802).
Addition to Methods section:
“Replication was assessed by intersecting the significant variant to gene pairs reported in each external study with the significant variant to gene pairs identified in our dataset, and quantifying the proportion that overlapped.”

7) When determining “distinct eQTL effects in osteoarthritis tissues”, what test was used to determine that “genes (were) expressed exclusively in a single disease tissue”? Simply lacking an eQTL does not mean a gene is not expressed in a particular tissue, so was there a minimum expression level used to deem a gene expressed in a tissue?

We agree with the reviewer that absence of an eQTL signal does not imply lack of gene expression. To determine distinct eQTL effects across osteoarthritis tissues we did not infer tissue expression from the presence/absence of an eQTL signal.
We have applied a minimum counts per million (CPM) expression filter within each tissue using the standard edgeR’s *filterByExpr* function with default parameters as part of RNA-seq quality control. This retains genes expressed above a CPM threshold, computed as CPM_cutoff = (10 / median(library size)) × 10^6, in a sufficient number of samples; for sample sizes >10 this corresponds to requiring expression in at least 70% of tissue samples. Under these default settings, the CPM cutoff was 0.292 for cartilage, 0.267 for synovium, and 0.279 for infrapatellar fat pad. Genes that did not pass this threshold were excluded from eQTL testing. Thus, the step of defining expression in a single tissue was performed prior to conducting the eQTL mapping and is based on tissue expression.

In addition, to distinguish true distinct effects among tissues from apparent absence due to limited power (due to varying sample sizes) for genes expressed across tissues, we have assessed cross-tissue sharing using mashR (Urbut et al., 2019), which models effect-size sharing while accounting for differences in statistical power. This approach allowed us to distinguish between genes expressed exclusively in a single tissue (as defined by the expression filter) and cases where the absence of an eQTL could reflect limited power. All analyses have now been repeated after incorporating the reviewer’s earlier suggestions (QC with VerifyBamID, contamination filtering, and PEER re-estimation with known covariates), ensuring that the results are based on high-quality, appropriately controlled data. We have amended the manuscript text to make this clear.

We have now updated the manuscript to include additional details that clarify the methodology.

See updated Methods paragraphs:
Preprocessing and quality control (QC) of RNA-sequencing data (Lines: 621-649)
Identification of tissue-sharing and single-tissue eQTLs (Lines:813-829)

See updated Results paragraph:
Distinct and shared eQTL effects in osteoarthritis tissues (Lines: 129-150)

Addition to Results section:
"Firstly, we investigated eQTLs regulating genes expressed exclusively in a single disease tissue and not in others based on a minimum gene expression threshold (see Methods), identifying 606 unique eGenes in cartilage, 74 in synovium, and 13 in fat pad (Table S3). Secondly, to formally assess distinct genetic regulatory patterns across osteoarthritis tissues for commonly expressed genes, we applied mashR[18], which models shared and distinct patterns of genetic regulation across all examined disease tissues (low-grade cartilage, high-grade cartilage, synovium and fat pad), and identify 62 additional eGenes with eQTL effects exclusive to low-grade cartilage, 23 to high-grade cartilage, 385 to synovium, and 70 to fat pad, with no effect in any other analyzed osteoarthritis tissue (Figure S4 , Tables S4)."

Addition to the Methods section:
"For each tissue we filtered lowly expressed genes using the *filterByExpr* function from edgeR[64] using default parameters as previously described[19], retaining genes that were expressed with more than 10 counts for the 70% of tissue samples. The CPM cutoff for each tissue under default parameters was 0.292 for cartilage, 0.267 for synovium, and 0.279 for infrapatellar fat pad."

8) Was the transcription factor disruption analysis performed on all identified trans-eQTLs? Or just the lead variant? Was there any additional information suggesting which trans-eQTLs were in regulatory elements and thus more likely to bind a TF? Why was this only done on trans-eQTLs and not cis-eQTLs, where likewise many variants could be disrupting TF binding?
For the transcription factor (TF) binding analysis, we did not restrict to the lead variants but considered the full set of *trans*-eQTL variants. As per the reviewer's suggestion, to point to *trans*-variants more likely to affect TF binding we have now further restricted this analysis to the ones overlapping regulatory regions and also considered transcription factor motifs only from transcription factor genes expressed within each respective tissue (using the minimum CPM expression threshold as indicated the previous response). Using this approach, we have now identified 17 unique *trans*-eQTLs among tissues predicted to affect transcription factors motifs (updated Table S9).

To extend this analysis to the *cis*-eQTLs as per reviewer's suggestion, we have now also performed TF binding motif analysis on the colocalizing *cis*-eQTLs (i.e., those also associated with disease traits). For each variant in the colocalizing credible set we have now used the same approach as in *trans*-eQTLs (i.e., evaluated the regulatory potential and transcription factor genes expressed within each respective tissue). We find 19 unique credible set variants overlapping regulatory promoter or enhancer regions that affect the binding of 38 unique TFs. We now provide

a mechanistic hypothesis for several of these variants in the manuscript Results section (see Table 1).

See updated Results paragraphs:
Trans-eQTLs is osteoarthritis primary tissues (Lines: 188-228)
Integration with functional data reveals mechanistic insights for osteoarthritis risk loci (Lines: 360-448).

See updated Method paragraphs:
Overlap between variants in the 95% credible set of the colocalization with functional data (Hi-C, ATAC-seq, SCREEN) (Lines: 888-909),
Transcription factor binding motif analysis (Lines: 911-922).

Addition to the Methods section:
"To prioritize colocalized variants from our analysis we integrated the 95% credible set variants from coloc.abf() results as well as all lead signals colocalizing in coloc.susie() with data capturing putative regulatory relationships. In particular, for low-and high-grade cartilage we used 3-dimensional spatial organization data of chondrocytes generated in an earlier study[30], ATAC-seq[42] and Histone mark data (H3K4me1, H3K4me3 and H3K27ac)[47] to identify variants which reside in active enhancers and promoters in chondrocytes that show physical interaction. For synovium and fat pad, given the lack of publicly available matching datasets, we restricted this step to variants overlapping promoters, as annotated in the SCREEN database[43] .
Enhancers and promoters were selected based on annotations from the latest ENCODE SCREEN data release (V4)[43] . First, all regions labelled as enhancers (pELS for proximal enhancers and dELS for distal enhancers) and promoters (pELS) were extracted and overlaid with ATAC-seq regions for low-grade (outer region of lateral tibial plateau - oLT region) or high-grade cartilage (inner region of medial tibial plateau - imT region). Valid ATAC-seq peaks used in our analysis were defined as peak regions identified in six out of eight patient samples. These regions in an open chromatin state were further overlaid with regions binding to a combination of H3K4me1 and H3K27ac for enhancers and H3K4me3 and H3K27ac for promoters respectively. Only regions found in two out of three replicates with these histone marks were labelled as active. For promoters with colocalized variants the gene which is annotated in the SCREEN[43] database was assigned as the target gene. For variants in active enhancer regions, those genes with active promoters in the loop anchor region being in contact with the loop anchor region of the active enhancer were annotated as target genes."

9) In the Variant Annotation of eQTL methods section, it mentions Fig S20 which has "distributions of eQTL annotations among both cis- and trans-eQTLs". This is not mentioned anywhere else and its purpose is not explained. Nor is it explained what "evaluation" was done.
We thank the reviewer for pointing out the lack of clarity. This figure (currently Figure S3) was included to summarize the functional annotation of both *cis*- and *trans*-eQTLs, showing the relative distribution of their overlaps with different genomic categories (e.g., coding regions, UTRs, introns, intergenic, regulatory elements). The "evaluation" referred to in the methods denotes this descriptive comparison (overlap) of annotation frequencies between *cis*- and *trans*-eQTLs, rather

than an additional statistical test. We have now revised the text to explicitly describe the purpose of Figure S3 as providing context on the genomic landscape of the eQTLs.

See Methods paragraph:
eQTL variant annotation (Lines: 841-855)

See updated Results paragraphs:
Osteoarthritis primary tissues cis-eQTL discovery (107-120)
Trans-eQTLs is osteoarthritis primary tissues (188-228)

See updated Results text:
"The majority of eQTLs overlap non-coding intronic and intergenic regions in agreement with other studies[17] (Figure S3)."
See updated Methods text:
"Figure S3 shows the distribution of functional annotations for cis and trans-eQTL variants, summarizing their overlap with different genomic categories."
"To explore potential mechanisms underlying these distal regulatory effects, we next asked whether *trans*-eQTL variants overlap regulatory regions. We find 84 *trans*-eQTLs in synovium, 35 in low-grade cartilage and 16 in high-grade cartilage overlapping regulatory regions (Table S9, Figure S3)."

Interpretation concerns/Text suggestions.
1) eQTL provide evidence that genetic variation affects the transcriptional regulation of the target gene. They do not "reveal the function of disease associated variants", but rather provide hypotheses for how GWAS variants may contribute to disease. Simply being colocalized with a GWAS signal does not confirm causality.
We have now revised the text to explicitly frame colocalization results as hypothesis-generating rather than causal.

Addition to the Introduction section (lines: 79-80)
"The regulatory relationships captured by eQTLs can generate hypotheses for the function of disease-associated variants identified through GWAS[12]"

2) It's not clear why the authors are treating their prior study (Steinberg, et al) as a seemingly independent study in the Introduction when the current study is a direct extension of that study with overlapping patient samples.
We have now revised the Introduction to make the continuity explicit.

Addition to the Introduction section (lines: 95-102):
"Here, we have enhanced the sample size of our previous study[14] and generated *cis*- and *trans*-eQTL maps from knee joint primary tissues of 320 osteoarthritis patients of European ancestry, including macroscopically intact (low-grade) and degraded (high-grade) cartilage, synovium, and fat pad."

Addition to the Results section (lines: 108-109) :

"We performed *cis*-eQTL analyses in 320 knee osteoarthritis patients, representing a > 3-fold increase in sample size compared to our previous study[14] (Figure 1, Figure S1, Table S1)."

3) eQTL being found only in a single tissue does suggest that regulation of that gene in that tissue is different from the other tissues, but that is all. It does not provide evidence that the genetic variation shown to affect this regulation is related to disease, unless there is more direct evidence for this, i.e. through GWAS. It is not clear why, then, unique eQTL in tissues for genes previously associated with osteoarthritis are that significant other than it supports that the gene is differentially regulated in that tissue. Do you also see significant differential expression? If yes, then this differential regulation could point to potential regulatory changes that lead to this differential expression. Similarly, identifying common eQTL across tissues simply shows that the regulatory mechanism affected by genetic variation is common across tissues. It likewise does not support a role for that genetic variation in disease, so the point of this analysis needs to be clarified.

We agree with the reviewer that detecting an eQTL in only one tissue on its own does not provide evidence that the underlying variant is related to osteoarthritis. The aim of our unique versus shared eQTL analysis was to characterize how regulatory effects differ across osteoarthritis tissues and to help prioritize tissues for downstream functional follow up, rather than to imply disease association beyond the fact that these are disease tissues. We have now revised the manuscript accordingly. In particular, we have removed examples and phrasing that could be interpreted as linking single-tissue eQTLs to disease, and we now restrict any discussion of disease relevance to analyses that provide more direct support, i.e, GWAS based evidence. We have also clarified terminology throughout, avoiding "disease progression" in this context and describing stage related signals explicitly in terms of cartilage degeneration, low-grade versus high-grade cartilage.

In line with the reviewer's suggestions, and to better contextualize the cartilage-grade specific signals, we have added two complementary analyses. First, we have now examined differential expression for genes with cartilage-grade specific eQTLs, focusing on the low-grade versus high-grade cartilage comparison, and we now report the extent to which genetic regulation coincides with stage dependent expression changes.
Second, we have now cross checked genes with unique eQTLs against our GWAS colocalization results, so that we only highlight overlap with disease-associated genetic signals when supported by colocalization. Together, these additions clarify the point of the analysis, unique and shared eQTLs describe regulatory architecture across tissues, while disease relevance is only inferred where independent GWAS based evidence is present.

Addition to the Results section (lines: 151-176):

Cartilage-grade dependent eQTLs reveal stage-specific genetic regulation

“In order to understand if the differential regulation between high- and low-grade cartilage is associated with expression changes related to progression of cartilage degeneration we further queried the differentially regulated eGenes against differentially expressed genes between high- and low-grade cartilage, defining differentially expressed eGenes (DE-eGenes)[19]. We find that 70 out of the 143 differentially regulated eGenes are also differentially expressed between high- and low-grade cartilage, indicating coordinated changes in both genetic regulation and transcriptional output as osteoarthritis progresses. Despite no significant enrichment of these genes for a specific pathway top DE-eGenes for low-grade cartilage include RNA-processing and splicing regulation components (*YJU2*, *GPATCH1*) and several lncRNAs (*MCPH1-AS1*, *LINC01198*, *CAPN10-DT*) while for high-grade cartilage we find genes involved in mitochondrial and metabolic processes (*ACO2*, *PGAM5*, *ENOPH1*, *PEX16*), extracellular matrix remodelling (*MATN1*, *MMP10*) and cell-cycle regulation (*TK1*, *ASXL1*).”

4) Related to the above, the idea of a “disease progression eQTL” is not well-defined. Again, eQTL found in one of low-grade vs high-grade samples simply suggests that the regulation of that gene changes. If the gene is also differentially expressed between the two, then this points to potential regulatory changes driving this change in expression. The fact that this regulation is affected by genetic variation does not, in itself, suggest that the genetic variation is related to disease progression. The significance of these findings need to be better explained if there is something I am missing.

We thank the reviewer for this comment. In the revised manuscript, we have now replaced the phrasing (“disease progression”) and now describe these signals as genetically regulated differences associated with cartilage degeneration within osteoarthritis tissue, which more accurately reflects what is being measured (namely differences between low-grade and high-grade cartilage).

We also agree that observing an eQTL in only one of the two cartilage grades, by itself, indicates a difference in genetic regulation between grades, but does not imply that the underlying variant is related to disease progression. To better contextualize these findings, we have now added differential expression analyses for genes with cartilage grade specific eQTLs. This allows us to highlight cases where grade-dependent genetic regulation coincides with observed expression differences between low grade and high grade cartilage, which is consistent with a regulatory contribution to stage-related transcriptional changes.

Among the genes showing differential genetic regulation between cartilage grades, we have now identified 70 genes that also show differential expression between low-grade and high-grade cartilage, and we now report these results in the revised Results section and corresponding supplementary material.

See updated Results paragraph: Cartilage-grade dependent eQTLs reveal stage-specific genetic regulation) (Lines: 151-176).

See Methods Paragraph (lines: 831-839)

“Identification of cartilage-grade specific eQTLs
To identify cartilage grade specific eQTLs, we applied the same MashR[18] based framework as for tissue sharing, but restricted to genes expressed in both low- and high-grade cartilage (N = 15,476), and defined an eQTL as grade-specific if it met LFSR < 0.05 in one grade, LFSR ≥ 0.1 in the other grade, had at least a two fold larger absolute effect size in the specific grade, and an absolute effect size of at least 0.1. To relate grade-specific genetic regulation to cartilage degeneration, we additionally queried the eGenes that were differentially regulated against genes differentially expressed[19] between low and high-grade cartilage, defining differentially expressed eGenes (DE eGenes).”

See Discussion Paragraph (lines: 540-547):
“We identify genetic regulatory effects that vary with cartilage degeneration and nominate genes for which these grade specific eQTLs are likely to contribute to expression differences between disease stages. These dynamic, context dependent effects, which would not have been captured by stage agnostic analyses, indicate that genetic regulation of transcription can shift as cartilage pathology progresses and may therefore point to mechanisms relevant to disease progression. However, inferring true disease progression eQTLs remains challenging, given the complex, heterogeneous nature of osteoarthritis and the fact that disease progression is also driven by a combination of environmental and genetic factors.”

5) Hi-C data showing links between enhancers that contain an eQTL and the promoter of a target gene provide support for specific variant in the enhancer being the casual variant of the eQTL effect. It also gives a hypothesis for the mechanism of the variant. Why do you assume that this “alters enhancer-promoter interactions”? This may be true, but more specifically, is there evidence for altering transcription factor binding in the enhancer? This would provide evidence for the variant altering TF binding which then changes how the enhancer regulates that target gene. In theory, this could lead to altered enhancer/promoter interactions, but that would be better seen in Hi-C data. Similarly, why not also look for TF disruption for the variants in promoters (related to the question of why TF disruption only done on trans-eQTLs)?
We thank the reviewer for this insightful comment. We have now revised the text to remove statements implying “altered enhancer promoter interactions” and instead describe the Hi-C data as supporting putative enhancer to promoter connections that can help nominate target genes and mechanisms.

To strengthen the mechanistic interpretation, we have now also refined our functional annotation strategy and now focus on variants supported by GWAS colocalization, specifically variants in the 95% colocalizing credible sets. For these variants, we have assessed overlap with active regulatory regions, enhancers and promoters, and have now tested whether they are predicted to disrupt transcription factor binding motifs, providing a more direct hypothesis for how allelic variation could alter gene regulation.

For low-grade and high-grade cartilage, we have now integrated chromatin interaction and regulatory annotation resources, including Hi-C, ATCA-seq, Chip-seq from cartilage-specific

publicly available data and the SCREEN database. For each credible set variant, we have evaluated whether it overlaps an active enhancer or promoter and, where Hi-C support is available, whether an enhancer harboring the variant connects to the promoter of the corresponding eGene. We similarly have now assessed promoter overlapping variants and whether the genes linked by HiC/SCREEN agree with the eGenes from our eQTL analysis. For osteoarthritis synovium and infrapatellar fat pad, where tissue-specific Hi-C is not available, we have evaluated whether credible set variants overlap SCREEN defined promoters and whether the SCREEN gene assignments are consistent with the corresponding eQTL eGenes.

To address the reviewer's point regarding whether variants may affect transcription factor binding in regulatory regions, we have now applied motif disruption analysis uniformly to credible set variants overlapping either enhancers or promoters, rather than limiting this analysis to *trans*-eQTLs. We have now further restricted the analysis to motifs corresponding to transcription factors genes that are expressed in the respective tissue. We have highlighted representative loci in Table 1, where integrating regulatory overlap, chromatin linking, and predicted motif disruption supports specific, testable mechanistic hypotheses.

We note that TF motif disruption predictions provide useful, testable mechanistic hypotheses, but tissue-specific interpretation is currently limited by the lack of well-powered, tissue-specific ChIP seq datasets. Follow-up studies, such as allele-specific chromatin accessibility assays, reporter or MPRA assays, and targeted CRISPR-based perturbation or base editing, will help validate and refine the proposed mechanisms. We have added this caveat to the Discussion.

Addition to the Results section: see paragraphs:
Integration with functional data reveals mechanistic insights for osteoarthritis risk loci (Lines: 360-448) *Trans*-eQTLs in osteoarthritis primary tissues (Lines: 188-228)

Addition to the Discussion section (Lines: 572-576):
"Future work complemented by time-course or perturbation experiments in relevant joint cell models, will help resolve these effects. In addition, the prioritised effector genes with their associated risk loci provide testable hypotheses that should be refined through targeted functional assays such as CRISPRi or CRISPRa and allele specific reporter experiments."

6) In the initial GWAS colocalization discussion, it would be helpful to indicate the different knee phenotypes that were studied with GWAS and make up the 211 loci. This would help when in the prioritization section, these specific phenotypes are mentioned.
We thank the reviewer for this suggestion. We have now revised the GWAS colocalization section to list the knee phenotypes contributing to the loci, so that the later prioritization results can be more easily contextualized.

Addition to the Results section (Lines: 229-240):
"We conducted statistical colocalization analysis between primary knee osteoarthritis tissue cis-eQTLs and GWAS risk loci from three knee-related osteoarthritis phenotypes: osteoarthritis at

any site (ALLOA), knee osteoarthritis (KNEE) and total knee replacement (TKR) (Tables S10-S11)."

7) The spatial transcriptomics data is only for cartilage. What is the significance of a prioritized effector gene that was not colocalized in cartilage being expressed in a cartilage layer (i.e. for HLA-DPB1, KCNN3, LTBP1, etc)? What is the significance of these being found in the superficial layer? More generally, is there other evidence pointing to the increased importance of this layer in arthritis that would support the importance of most of these genes being expressed in that layer?

We agree with the reviewer that the spatial transcriptomics data is only relevant for cartilage genes. We have now excluded from this analysis genes that colocalize with osteoarthritis only in synovium to avoid overinterpretation.

Addition to the Results section (lines: 263-276):

"To further understand the spatial context of the prioritized genes colocalizing with osteoarthritis, we evaluated the expression patterns of the genes using spatial transcriptomics data from degraded osteoarthritis cartilage (see Methods). We find that all 27 prioritized genes colocalizing with osteoarthritis in cartilage are expressed in at least one of the analyzed cartilage zones (Table S15), with all being detected in the superficial layer of osteoarthritis cartilage. Seven genes (*ALDH1A2, FBN2, LMX1B*,*NDNF*, *NMB*,, *SMAD7* and *TMEM129*) are also expressed in the middle zone, while two genes (*ALDH1A2* and *RBPJ*) are expressed in the deep cartilage zone. This shows that the majority of prioritized genes are active in the superficial layer of osteoarthritis cartilage. This layer is the first point of mechanical loading in the joint and exhibits the earliest pathological changes in osteoarthritis. It contains specialised superficial chondrocytes that produce key homeostatic proteins, including lubricin and tenascin, which are essential for cartilage lubrication and surface integrity. The observed enrichment therefore highlights the central contribution of superficial-zone dysfunction to early disease pathogenesis[37]."

8) Since most of the prioritized genes were eQTL in cartilage, is it really surprising that pathway enrichment points to chondrocyte related pathways? This seems more like an analysis confirming the function of the prioritized genes than something novel.

We agree that enrichment of chondrocyte-related pathways is expected given the cartilage-derived eQTLs. However, to our knowledge, previous eQTL studies have not explicitly highlighted these pathways, and we view it as an important confirmation that our dataset recapitulates known biology while also extending it by identifying additional genetically regulated genes within these pathways (see amended Results section). In addition to capturing known osteoarthritis biology, our analyses also highlight further pathways and processes, which we have now emphasized as potentially novel biology. In line with the comments of reviewer 2, we have now conducted additional pathway analyses on genes that colocalize with osteoarthritis and are either upregulated or downregulated in respect to the risk-increasing osteoarthritis signal.

Addition to the Results section (Lines: 304-322):

"To refine the biological interpretation of the colocalizing loci, we further examined the direction of allelic effects for each variant in the credible sets. For each colocalization, the osteoarthritis

risk allele showed either consistently concordant effects on gene expression (risk allele increases expression) or consistently discordant effects (risk allele decreases expression). This enabled us to classify colocalizing genes into putative "risk-increasing when up-regulated" and "risk-increasing when down-regulated" groups. Only one gene (*LMX1B*) showed opposite classifications in high-grade cartilage and synovium relative to knee osteoarthritis risk. When evaluating pathways separately for the two groups, the enrichment patterns were modest (Table S15), reflecting that osteoarthritis genetic risk perturbs both activating and inhibitory components of the same regulatory pathways (Figure 5A).

Consistent with this, the most strongly enriched processes are represented by a connected network of TGF-β/BMP signaling components (*SMAD3*, *SMAD6*, *SMAD7*, *GDF5*), FGFR signaling (*FGFR3*), matrix modulators (*FBN2*, *CHRDL2*), and lineage regulators (*MUSTN1*, *PBXIP1*, *CEBPB*, *PAX9*, *NFATC1*). In this network, "risk-increasing when up-regulated" genes largely correspond to pro-chondrogenic and pro-hypertrophic drivers, whereas "risk-increasing when down-regulated" genes capture feedback inhibitors and ECM/growth-factor modulators, illustrating that osteoarthritis risk putatively acts by both amplifying chondrogenic drive and weakening its restraint (Figure 5B)"

9) Druggability search – while it is interesting that some of the genes identified already have drugs that target them specifically for use in arthritis (confirmation of role of that gene in disease), the more interesting are the ones whose drugs were not developed for arthritis. I would have thought more of this section would be focused on discussing these and their potential. What are the drugs currently being developed for? Is the action of the drug in the right direction (i.e inhibitory/excititory) given the direction of effect of the eQTL compared to the GWAS association? Is the drug suitable for long-term use, given this is a chronic condition? I feel a lot more should be explored here.
We thank the reviewer for raising this point. In the updated version of our manuscript, we have now further developed the druggability analysis section. Firstly, we have focused our discussion on drugs targeting the colocalizing genes that are approved for different indications as these constitute potential drug repurposing opportunities for osteoarthritis. Secondly, we have now incorporated the causal direction of effect identified in our MR analysis into the discussion for specific genes targeted by approved drugs. We find several examples for which the MR direction of causal effect of gene expression on osteoarthritis risk is concordant with the mechanism of action of the approved drugs. For instance, we highlight approved drugs targeting *SMAD7*, indicated for inflammatory diseases such as Chron's disease, and *LGALS3*, indicated for fibrotic diseases. Both indications are chronic conditions, denoting long-term use safety.

See updated Results paragraph:
Translational drug repurposing opportunities for the colocalizing genes (Lines: 458-510)

10) MUSTN1 was highlighted in the abstract, but it's not clear why. While there was a paragraph devoted to it in the Prioritized Genes results section, the discussion of this gene was similar to many other genes highlighted in the paper. It was not mentioned in the Introduction nor was it a

further focus within the Discussion section. Thus why it was chosen for special recognition in the abstract should be made more clear.
We have updated the Abstract according to the reviewer's suggestion. We have now highlighted genes targeted by approved drugs that are not currently indicated for osteoarthritis, but for which we present evidence of directional concordance between the inferred causal effect of gene expression on osteoarthritis risk and the known therapeutic mechanism of action.

Addition to the Abstract:
"Finally, we provide compelling directional evidence highlighting drugs targeting *LGALS3* and *SMAD7* as repurposing opportunities for osteoarthritis treatment."

Reviewer #2 (Remarks to the Author)

This manuscript interesting and very relevant by Katsoula and colleagues, addresses a significant gap for osteoarthritis genomics research, the identification of OA genes and pathways. Specifically, the strengths of this manuscript are in the use large data sets for eQTL analysis and the investigation of multiple OA relevant tissues, this is cartilage (high- grade, and low-grade), synovium and fat pad. Although, the data itself is highly relevant for the OA field, the manuscript itself lacks depth for the data itself to truly contribute to the field of OA genomics and possible future drug development.
We thank the reviewer for their time in reviewing our manuscript and providing comments that substantially helped increase the impact of our work.

There are three OA relevant tissues examined for eQTLs (High grade/low grade Cartilage, synovium and fat pad), as OA is a disease of the whole joint. Some of these eQTLs are deemed to be tissue specific. How truly tissue specific are these eQTLs? Or how OA specific are they truly? Considering that only 3/4 tissues were examined, with also differences in detection power. Fat pad has a vastly lower sample size then the other examined tissues. It is of course impossible to examine all possible tissues (time/data availability/scope), but there are some already existing eQTL data sets which the authors could use to bring more validity and robustness to their claims of tissue specificity; There have been a few publications from the GeFos consortium (PMID: 37579195, PMID: 36416764) where bone (osteoclast) eQTL's have been investigated, also in the context of OA specific eQTLs. Or, using the GTEX database to validate that the found "tissue specific"QTLs are indeed tissue specific to the OA tissues.
We agree with the reviewer that it is not possible to comprehensively test tissue specificity with the available data. In particular, comparison with GTEx is limited because healthy cartilage, synovium and fat pad are not represented, and thus we cannot claim "cartilage/synovium/fat-pad-specific" regulation in a strict sense. What our analysis provides is a comparison across the three osteoarthritis primary tissues, framed as differences in regulatory patterns rather than absolute specificity. We have now amended the phrasing in the manuscript to convey this.

We thank the reviewer for pointing us to the GeFOS consortium studies. We have evaluated whether these datasets could serve as an external benchmark for our tissue comparisons. The

osteoclast eQTL study (PMID: 37579195) was generated from differentiated cells derived from PBMCs rather than primary joint tissues, and therefore does not provide a direct reference for assessing tissue-restricted regulation in primary osteoarthritis joint tissues. The second study (PMID: 36416764) is a bone mineral density GWAS that leverages GTEx eQTLs for colocalization and TWAS, but does not provide an eQTL resource in osteoarthritis-relevant joint tissues that could be used to compare the primary tissue eQTLs in our setting. We have now clarified limitations and the challenges of defining tissue-specificity in the Discussion.

Finally, we acknowledge the reduced sample size for infrapatellar fat pad. To mitigate differences in detection power across tissues, we have used mashR (Urbut et al., 2019), which directly addresses this issue and models effect size sharing across tissues while explicitly accounting for unequal power and measurement noise. This is particularly well-suited to our study design because all tissues were collected from the same individuals, allowing mashR to learn the cross-tissue covariance structure directly from the data. This framework helps distinguish effects that are genuinely tissue enriched from effects that may appear tissue restricted primarily due to limited power in one tissue. We have now clarified this in the manuscript.

Addition to Discussion (Lines 566-569):
“We also note substantial differences in sample size across tissues, particularly for fat pad, which limits direct comparability of discovery rates across tissues, a limitation we partially mitigated using multi tissue methods such as mashR[18] that models shared effects across tissues.”

For each tissue examined one or two “tissue specific” eQTLs are described in the results with their relation to OA biology/pathology based from literature. The selection of the gene to described seem very arbitrary, also as their functions in OA pathology are then somewhat extrapolated to the full set of eQTL’s identified in those tissues. However, multiple tissue eQTL’s have been were found (597 unique eGenes in cartilage, 70 in synovium, and 14 in fat pad). Could the authors not perform a formal pathway analysis to describe the identified genes and link them to OA biology in a more structured way? This is also present in the section on differences between high grade and low grade cartilage.
We thank the reviewer for this suggestion. In the main text, we had highlighted only the top eQTLs per tissue as illustrative examples of regulatory patterns, but we agree that a more systematic overview strengthens the analysis. Since eQTLs capture general regulatory variation and not direct links to osteoarthritis, we have now focused pathway enrichment on the subset of eGenes colocalizing with GWAS signals in each tissue, thereby linking regulatory patterns more directly to osteoarthritis biology. We have systematically performed pathway enrichment analysis for all colocalizing genes, and separately for the subset of genes colocalizing within each tissue. We have now updated the Results section with the new insights.

Addition to Results Section: Biological pathways and direction of effect at colocalising loci (Lines 392-322):

“To glean biological insights from the 136 colocalizing genes, we evaluated their enrichment among biological pathways using all eGenes across tissues as background (Table S15). We find

that colocalizing genes are enriched for chondrocyte proliferation (q-value=3.2×10^{-3}), cartilage development (q-value=0.021), ossification (q-value=0.028) as well as signaling through transforming growth factor beta (TGF-β) (q-value=0.042) (Figure 5A). These processes have been previously implicated in osteoarthritis[34–36].We also performed enrichment analyses separately for each tissue colocalizing genes implicating processes such as amino sugar and nucleotide sugar metabolism (q-value=0.005) and osteoblast differentiation (q-value=0.048) in low-grade cartilage, osteoarthritic chondrocyte hypertrophy (q-value=0.012) and ossification (q.value = 0.045) in high-grade cartilage and chondrocyte proliferation (q.value = 0.002) in synovium (Table S15)."

The strengths and important elements of eQTL research for GWAS effector gene identification, is not only the identification of a possible effector gene, but equally so the direction of effect. What is the effect of the OA risk allele on which gene, and the direction part is under developed in this manuscript, a part from the very interesting section on discordant eQTLs. For drug development purposes (or experimental validation) it is vital to understand if the OA risk allele cause for an increase or decrease of the gene expression (i.e. does a OA drug need to increase of decrease the gene expression or pathway). Within the tables the effect/modelled allele is either not stated or not clearly stated – Examples table 2 has the Allele Frequency (AF) but does not state the alleles this AF belongs to or if it represents the effect allele frequency. Table 2: does report the alternate (minor) allele frequency and the allele. However, it is unclear if this is then also the effect or modelled allele. Also the figures do not clearly report or indicate what the effect allele is for the eQTL variant. (figure 3, figure 4)
We agree with the reviewer that generating insights into the direction of effect of effector genes on disease risk is important. To address this, we have now revised the text to emphasize the directionality of eQTL effects more explicitly, beyond the section on discordant eQTLs.

We have updated all supplementary tables (Tables S2-S9) and have added Table 1 to clearly indicate the effect (modeled) allele, along with its frequency, so that it is unambiguous which allele increases or decreases expression of the effector gene. We have also clarified in the table legends and figure captions whether the reported frequency corresponds to the effect allele frequency.

We have also run Mendelian randomization analysis to find evidence of directional causal relationships between variants associated with gene expression and osteoarthritis risk. Following the reviewer's suggestion, we have now explored the overlap between the MR causal direction of effect of the prioritized effector genes and and the effect of approved drugs targeting the prioritized genes.

Addition to Results Section: "Biological pathways and direction of effect at colocalising loci (Lines 304-313):

"To refine the biological interpretation of the colocalizing loci, we further examined the direction of allelic effects for each variant in the credible sets. For each colocalization, the osteoarthritis risk allele showed either consistently concordant effects on gene expression (risk allele increases

expression) or consistently discordant effects (risk allele decreases expression). This enabled us to classify colocalizing genes into putative “risk-increasing when up-regulated” and “risk-increasing when down-regulated” groups. Only one gene (*LMX1B*) showed opposite classifications in high-grade cartilage and synovium relative to knee osteoarthritis risk. When evaluating pathways separately for the two groups, the enrichment patterns were modest (Table S15), reflecting that osteoarthritis genetic risk perturbs both activating and inhibitory components of the same regulatory pathways (Figure 5A).”

In addition, to the direction of effect of the OA risk alleles, this new eQTL information can also be used in the pathway analysis, i.e. are the found pathways up or down regulated for OA risk? It would be very valuable to the field to not just have another pathway analysis with more genes, but to have one which also takes into account whether or not according to the GWAS findings the enriched pathways/cellular processes are up or down regulated.
We thank the reviewer for their suggestion. We have now classified the colocalizing genes into upregulated and downregulated genes and have now run additional pathway analysis for each group. Upregulated colocalizing genes are those for which the genetic variants constituting the colocalizing signal have an increasing effect on gene expression levels regarding the osteoarthritis risk-increasing allele. Downregulated colocalizing genes are those for which the genetic variants constituting the colocalizing signal have a decreasing effect on gene expression levels regarding the osteoarthritis risk-increasing allele.

Updated Figure 5:

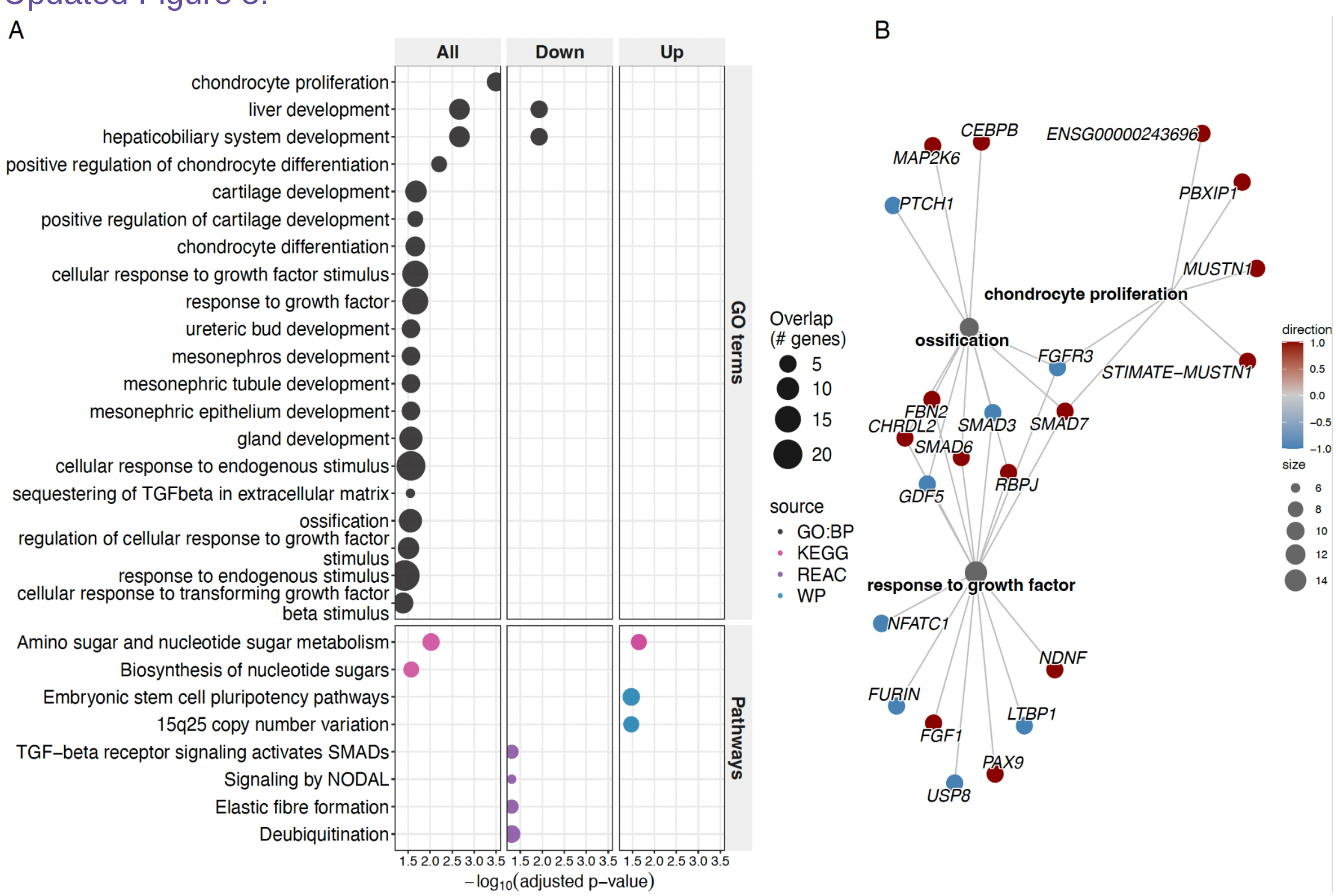

Figure 5: Pathway enrichment analysis of the colocalizing genes. (A) Results (q-value<0.05) of pathway-based over-representation analysis on the 136 genes colocalizing with knee-related osteoarthritis phenotypes. All eGenes across all four primary osteoarthritis tissues studied here were used as the background. Down refers to genes that are down-regulated relative to knee osteoarthritis' risk allele, whereas up refers to genes that are up-regulated. (GO:BP = Gene Ontology biological processes; REAC = Reactome; WP = WikiPathways) (B) Network of genes involved in the most strongly enriched processes of the pathway-based over-representation analysis. Red circles denote genes with a knee osteoarthritis risk-increasing effect when up-regulated, whereas blue circles denote genes largely risk-increasing when down-regulated. The grey circle size denotes the number of genes in the specific pathways.

Addition to Results Section (Lines: 315-322):
"Consistent with this, the most strongly enriched processes are represented by a connected network of TGF-β/BMP signaling components (*SMAD3*, *SMAD6*, *SMAD7*, *GDF5*), FGFR signaling (FGFR3), matrix modulators (*FBN2*, *CHRDL2*), and lineage regulators (*MUSTN1*, *PBXIP1*, *CEBPB*, *PAX9*, *NFATC1*). In this network, "risk-increasing when up-regulated" genes largely correspond to pro-chondrogenic and pro-hypertrophic drivers, whereas "risk-increasing when down-regulated" genes capture feedback inhibitors and ECM/growth-factor modulators, illustrating that osteoarthritis risk putatively acts by both amplifying chondrogenic drive and weakening its restraint (Figure 5B)."

Multiple trans eQTLs were identified, and the authors report that most of the trans-eGenes were also cis-regulated. However, it would be more interesting to know which trans eQTL variant also is a cis eQTL variant. This as most trans eQTLs are thought to represent downstream targets/pathways. Example: snp1 regulates cis-gene Y, snp1 also has trans-eQTL gene z. Suggesting gene y may regulate gene z (or gene z might be downstream of gene Y in the biological pathway). This could greatly strengthen and inform the pathway analysis or even indicate "novel" pathways or downstream targets for OA genes.

We thank the reviewer for this comment. As per the reviewer's suggestion, we have now examined whether *trans*-eQTL variants are also *cis*-eQTLs for other genes within each tissue. Using the full lists of significant trans eQTLs, we have now identified 129 such variants in low-grade cartilage, 93 in synovium, and 47 in high-grade cartilage.

These signals involve only a small number of genes (*trans*-eGenes: *UQCRHL*, *CBX3*, and *FHAD1*, and cis-eGenes: *UQCRH*, *FAAH*, *CCDC32*, *TSPAN1*, *LURAP1*, and *ZNF100*), which precludes a meaningful formal pathway enrichment analysis. Nevertheless, we have now highlighted representative *cis*- plus *trans*- relationships in the revised manuscript text.

Addition to the Results - *Trans*-eQTLs in osteoarthritis primary tissues (Lines: 196-212)
"To identify putative regulatory relationships between *trans*-eGenes and *cis*-eGenes we assessed whether *trans*-eQTLs also exert *cis* effects for additional genes. We identify 129 such trans-eQTLs also acting in cis cases in low-grade cartilage, 93 in synovium and 47 in high-grade cartilage. In

all three tissues, *trans*-eQTL signals for *UQCRHL*, acted in cis for *UQCRH*, pointing to a shared mitochondrial regulatory axis. In synovium and high-grade cartilage, additional cis targets of *UQCRHL* (*trans*-eGene) include *FAAH* and *LURAP1* (synovium) and *TSPAN1* (high-grade cartilage) respectively (Table S8).
In low-grade cartilage we uniquely find that trans-eQTLs for *CBX3* act in cis for *CCDC32*. These two genes have been previously found in a gene fusion in healthy blood and bone marrow tissues20. In synovium we also uniquely identify that *FHAD1* trans-eQTLs are also cis-eQTLs for *ZNF100*, suggesting a potential mediation relationship. Notably, *ZNF100* has been reported as a candidate effector gene for synoviopathy in Open Targets[21]. Complementarily, we also evaluated whether trans-eGenes were themselves also cis-regulated. We find that 11/13 in low-grade cartilage, 3/4 in high-grade cartilage, and 2/4 in synovium are also cis-eGenes in the same tissues. Out of these, notable examples include *CRHR1* and *KANSL1-AS1*, both located in the osteoarthritis-associated 17q21.31 locus, which show elevated expression in knee osteoarthritis cartilage compared to non-osteoarthritis controls[22]."

The limitations section in the discussion misses several important limitations of this study and it's findings; notably the strong difference in samples size, i.e., statistical power between the tissues investigated particularly for fat pad. The possibility tissue specific eQTLs may not be present in other not investigated tissues and that Transcriptomics is highly dynamic and that always eQTLs may be missed. (minor: an acknowledgement that all the identified effector genes, would still need (wet-lab) validation).
We have now expanded the limitations in the amended Discussion of the manuscript as per the reviewer's suggestion. In particular, we have explicitly acknowledged the substantial differences in sample size for fat pad tissue and the way that this sample size difference has been partially mitigated by using appropriate statistical frameworks (mashR, Urbut et al.2019) that enhance discovery and minimize noise by modeling shared patterns across tissues. Moreover, we have amended the manuscript text to clarify the lack of tissue-specificity for the reasons delineated in previous responses.

Addition to the Discussion section (lines: 566-576):
"We also note substantial differences in sample size across tissues, particularly for fat pad, which limits direct comparability of discovery rates across tissues, a limitation we partially mitigated using multi tissue methods such as mashR that model shared effects across tissues. In addition, our tissue panel does not exhaust all osteoarthritis relevant tissues, so regulatory effects operating in other joint tissues may be missed. As transcriptomes are dynamic, some regulatory effects may be context or time specific and therefore not captured in the tissues and conditions analysed here. Future work complemented by time course or perturbation experiments in relevant joint cell models, will help resolve these effects. In addition, the prioritised effector genes with their associated risk loci provide testable hypotheses that should be refined through targeted functional assays such as CRISPRi or CRISPRa and allele specific reporter experiments"

Minor comments:

Figure 3 and figure 4 seem to depict very similar results, as the high grade and low grade cartilage are both also depicted in figure 3. Could these two figures not be combined into one? Or at least an A and B panel?

We thank the reviewer for their suggestion. In the updated manuscript we have now combined Figures 3 and 4 into a single figure.

Updated Figure 3:

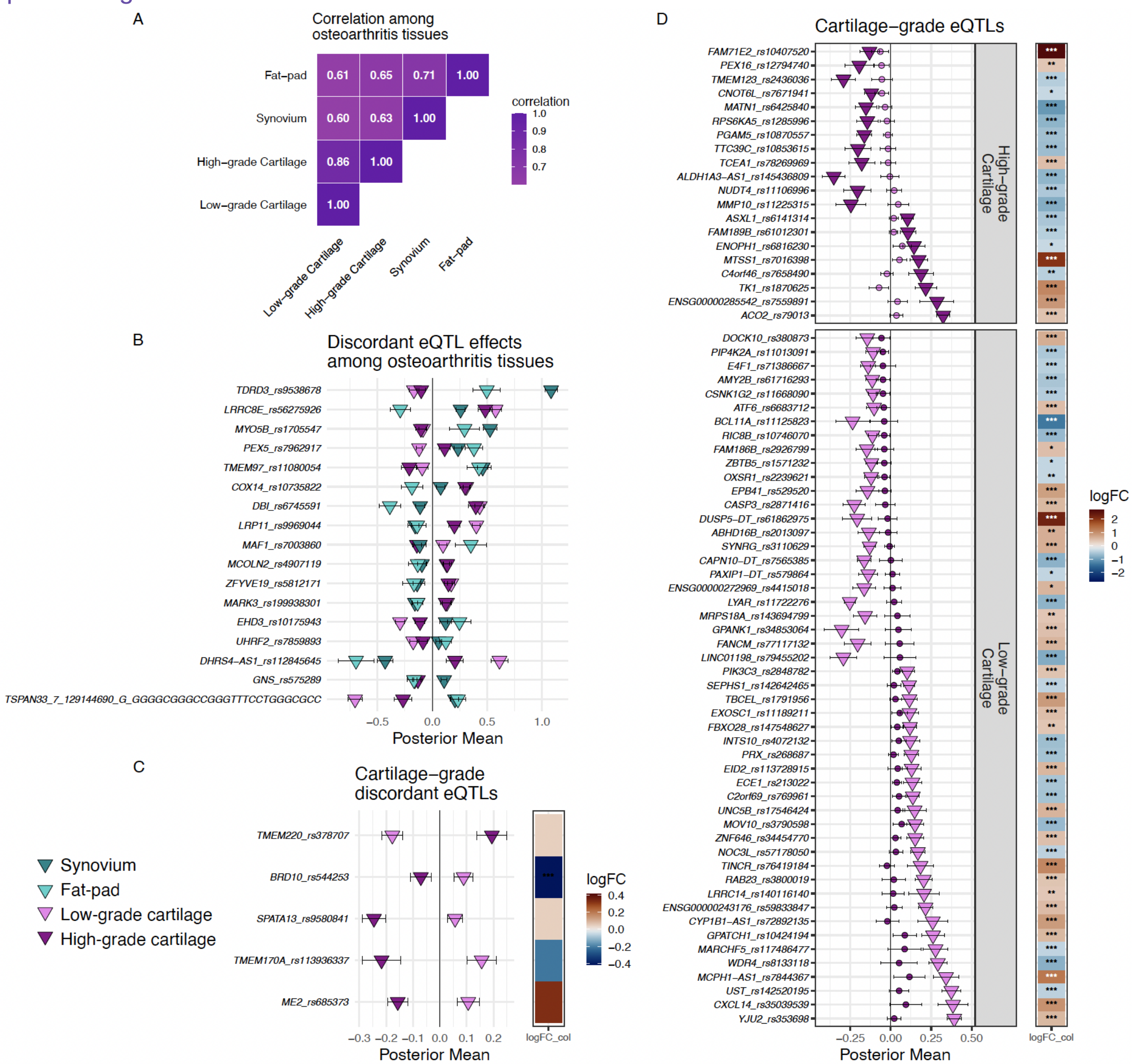

Figure 3: Patterns of shared and discordant eQTL effects among osteoarthritis primary tissues. (A) Heatmap showing pairwise sharing by magnitude of cis-eQTLs among tissues. (B) 17 eQTL–eGene pairs showing opposite directions of effect between osteoarthritis tissues. (C) Five eQTL–eGene pairs exhibit opposite directions of effect between low- and high-grade osteoarthritis cartilage, indicating stage-specific regulatory changes. (D) Cartilage-grade specific eQTLs. EQTL–eGene pairs unique to each cartilage grade influencing differentially expressed (DE) genes between low- and high-grade cartilage. Heatmap corresponds to differential gene

expression effect size (logFC) and significance to FDR adjusted p-values (DE reference: low-grade cartilage).

Identification of trans eQTLs – The reporting of the numbers of trans eQTLs is confusing and somewhat misleading, example: “363 transeQTLs (12 independent signals)”. As similar with GWAS signals many genetic variants are in high LD, leading to a high absolute number of genetic variants in association but due to the LD they just represent one GWAS signal. Thus here the more appropriate reporting is we identified 12 independent trans eQTLs. This is also how earlier in the text the cis eQTLs are presented. And it is actually already quite amazing that with this sample size 12 tras eQTLs were found, as the robust identification of them needs a huge sample size. So no need to make the number sound bigger.
We thank the reviewer for their comment. We **have now** reported the independent *trans*-eQTLs in the main Results section.

See updated Results paragraph:
Trans-eQTLs is osteoarthritis primary tissues (lines: 188-228):
“We investigated distal gene regulation (≥5 Mb from transcription start sites) in osteoarthritis primary tissues. In low-grade cartilage, we identify 13 independent signals regulating 13 *trans*-eGenes; in high-grade cartilage, 4 independent *trans*-eQTLs associated with 4 genes; in synovium, 4 independent *trans*-eQTLs associated with 4 genes; and no trans-eQTLs in fat pad. Four *trans*-eGenes showed cross-tissue overlap. *UQCRHL* was shared across all tissues. *ORC6*, *MYLK3*, and *KANSL1-AS1* were common to low- and high-grade cartilage, while *CDK10* was shared between high-grade cartilage and synovium (Table S8).”

Figure 7 – would be more informative if also the corresponding OA GWAS lead variant is/OA signal is added, Next, to what the variant is to which the direction symbol is referring to in the coloc columns.
We agree with the reviewer on the importance of directional evidence and have added these to the druggability section and to the pathway analysis results. In Figure 7 (now updated Figure 4), the direction symbol in the colocalization columns represent the direction of causal effect identified in our MR analysis. In short, the causal effects are derived through a weighted sum of the effect of genetic variants on the gene expression divided by the effect of the same variants on osteoarthritis risk. The genetic variants are selected based on their association with gene expression levels and not with osteoarthritis, hence they do not necessarily constitute an osteoarthritis signal.

REVIEWERS' COMMENTS

Reviewer #1 (Remarks to the Author):

The authors have done well to address my previous concerns. I do not have any additional concerns to raise. This study provides a robust analysis of eQTL in three primary tissues, one at two distinct stages, relevant for osteoarthritis. I believe it significantly adds to the interpretation of the effects of variants with GWAS loci for OA.

We thank the reviewer for their comment and the kind words about the study.

Reviewer #3 (Remarks to the Author):

The authors have generally addressed the major issues raised in the original review. I still have the remaining minor concerns that the authors should address:

We thank the reviewer for their comments.

1. The potential mechanisms of underlying trans-eQTLs (e.g, using motifBreakR) are not convincing; disruption of a transition factor binding site does not explain the mechanism of action of trans-eQTLs. If anything, such an analysis should be added for the cis-eQTLs.

We have amended the manuscript text and removed claims on the mechanism of action based on predicted disruption of a transition factor binding sites for the trans-eQTLs. We have added such an analysis for the colocalizing cis-eQTLs that overlap regulatory regions. See manuscript lines: 364-409.

2. It is unfortunate that the authors were not able to overlap their results with tissue-specific data from GTEx or GeFOS leaving the issue of tissue-specific eQTLs unresolved. If there truly is no such possible analysis, the authors should make sure to strike claims of specificity and note the shortcoming in the discussion.

We thank the reviewer for this point. Neither GTEx (which lacks healthy cartilage, synovium, and infrapatellar fat pad) nor the GeFOS datasets (osteoclasts derived from PBMCs rather than primary joint tissues) provide a suitable reference for benchmarking tissue-restricted regulation in primary osteoarthritis joint tissues.

In line with the reviewer's suggestion, we have amended the manuscript to remove claims of tissue specificity. We now frame our findings as differences in regulatory patterns across the three osteoarthritis primary tissues examined, rather than as evidence of tissue-specific

eQTLs in a strict sense. We have also expanded the Discussion to explicitly acknowledge this limitation, noting that without matched healthy-tissue or independent joint-tissue eQTL resources, true tissue specificity cannot be established from our data alone.
See manuscript lines: 524-527
