# Supplementary material for: Transcriptome analysis in osteoarthritis primary tissues identifies high-confidence effector genes
Source: Nat Commun. 2026 Jun 29;17:8128. doi: 10.1038/s41467-026-74993-y (PMC13458226; doi:10.1038/s41467-026-74993-y)
Supplement: Supplementary file 4 [file 41467_2026_74993_MOESM2_ESM.pdf]

Description of Additional Supplementary Files

Supplementary Data 1: Aggregate demographic data of individuals included in the study and summary technical covariate distributions across study samples.

Supplementary Data 2: Permuted significant eQTL results and conditional independent *cis*-eQTLs per osteoarthritis primary tissue. *Cis*-eQTLs mapped by linear regression (tensorQTL); gene-level significance by permutation (1,000 permutations) with Storey-Tibshirani *q*-value correction. Conditionally independent signals identified by stepwise regression.

Supplementary Data 3: *Cis*-eQTLs significant in only one osteoarthritis primary tissue, arising from gene expression unique to that tissue (genes passing the minimum counts-per-million (CPM) expression threshold in that tissue only). *C*is-eQTLs mapped by linear regression (tensorQTL) with permutation-based q-value correction; tissue-uniqueness based on the CPM expression threshold (see Methods).

Supplementary Data 4: Summary statistics from multivariate adaptive shrinkage (mashr) analysis across osteoarthritis primary tissues.

Supplementary Data 5: Summary statistics of unique and discordant eQTLs from cross-tissue analysis using mashr.

Supplementary Data 6: Summary statistics from multivariate adaptive shrinkage (mashr) across cartilage grades.

Supplementary Data 7: Summary statistics of disease grade-specific and discordant eQTLs across cartilage grades using mashr.

Supplementary Data 8: Significant *trans*-eQTLs per osteoarthritis primary tissue using MatrixEQTL. *Trans*-eQTLs mapped by linear regression (MatrixEQTL); genome-wide significance by FDR (q-value < 0.05) after correction for the expected number of independent tests (see Methods).

Supplementary Data 9: *Trans*-eQTLs overlapping regulatory regions and predicted to affect transcription factor motif binding. *Trans*-eQTLs as in Supplementary Data 8; transcription-factor motif effects predicted with motifbreakR (information-content method, threshold 1×10^{-5}).

Supplementary Data 10: Significant results of colocalization analysis using the coloc.abf() method between *cis*-eQTL data from osteoarthritis primary tissues and osteoarthritis GWAS. Evidence of colocalization was defined as PP4>0.8 or PP4>0.6 + PP4/PP3>2.

Supplementary Data 11: Significant results of colocalization analysis using the coloc.susie() method between *cis*-eQTL data from osteoarthritis primary tissues and osteoarthritis GWAS. Evidence of colocalization was defined as PP4>0.8 or PP4>0.6 + PP4/PP3>2.

Supplementary Data 12: Results of Mendelian randomization analyses between colocalizing *cis*-eGenes from osteoarthritis primary tissue *cis*-eQTL data and knee-related osteoarthritis phenotypes. Causal effects estimated by Mendelian randomization (inverse-variance weighted, or Wald ratio for single-instrument genes; TwoSampleMR); p-values FDR-adjusted for multiple testing. Sensitivity analyses (MR-Egger, weighted median, Cochran's Q, Steiger filtering) as described in Methods.

Supplementary Data 13: Scoring of colocalizing genes based on orthogonal lines of evidence pointing to involvement in osteoarthritis.

Supplementary Data 14: Nanostring GeoMX probe counts of spatial transcriptomics data from three osteoarthritis patients.

Supplementary Data 15: Results (*q*-value<0.05) of the pathway-based enrichment analysis on the 136 genes that colocalize with knee-related osteoarthritis phenotypes. One-sided over-representation analysis (g:Profiler2) against a custom background of all eGenes, Benjamini-Hochberg FDR correction, significance at FDR < 0.05.

Supplementary Data 16: Regulatory annotation of colocalizing GWAS and *cis*-eQTL signals using functional data.

Supplementary Data 17: Druggability information of colocalizing genes that are targets of drugs with at least a completed phase II trial.
